# Ion Diffusion and (Photo)redox Conductivity in a Covalent Organic Framework

**DOI:** 10.1021/jacs.5c17763

**Published:** 2026-01-15

**Authors:** Bibhuti Bhusan Rath, Bettina V. Lotsch

**Affiliations:** † Nanochemistry Department, 28326 Max Planck Institute for Solid State Research, Heisenbergstraße 1, 70569 Stuttgart, Germany; ‡ Department of Chemistry, Ludwig-Maximilians-Universität (LMU), Butenandtstr. 5-13, 81377 Munich, Germany; § E-Conversion and Center for Nanoscience, Lichtenbergstrasse 4a, 85748 Garching, Germany

## Abstract

Covalent organic frameworks (COFs) have emerged as promising materials for energy-related applications, where precise control over charge and mass transport is critical, such as in electrocatalysis and battery technologies. Despite ongoing debates on the mechanisms of charge transport in COFs—particularly band transport versus electron hopping—experimental evidence for redox conductivity via hopping remains limited. In this work, we investigate redox hopping-mediated charge transport in a naphthalene diimide (NDI)-based redox-active COF (TAPT–NDI COF), examining the influence of ion and solvent environment. We show that electron hopping through ion-coupled self-exchange between oxidized and reduced linkers is strongly affected by ion size, ion pairing, and solvent polarity, as evidenced by variations in the apparent electron diffusion coefficients, *D*_e_^app^, obtained through potential step chronoamperometry. Notably, we report the first observation of a potential-dependent, bell-shaped redox conductivity profile in COFs. Furthermore, the redox states of the NDI units can be systematically modulated by both electrical potential and light (NDI^0/•–^, NDI^•–/2–^ by applied potential and NDI^0/•–^ by light). The conductivity at intermediate redox states is enhanced by up to 4 orders of magnitude, enabling a highly reversible switching from an insulating (∼10^–9^ S cm^–1^) to semiconducting (∼10^–6^ S cm^–1^) regime. These findings offer new insights into redox transport in COFs and lay the groundwork for advancing their use in (photo)­memristive devices, sensors, and (photo)­electrocatalysis.

## Graphical abstract

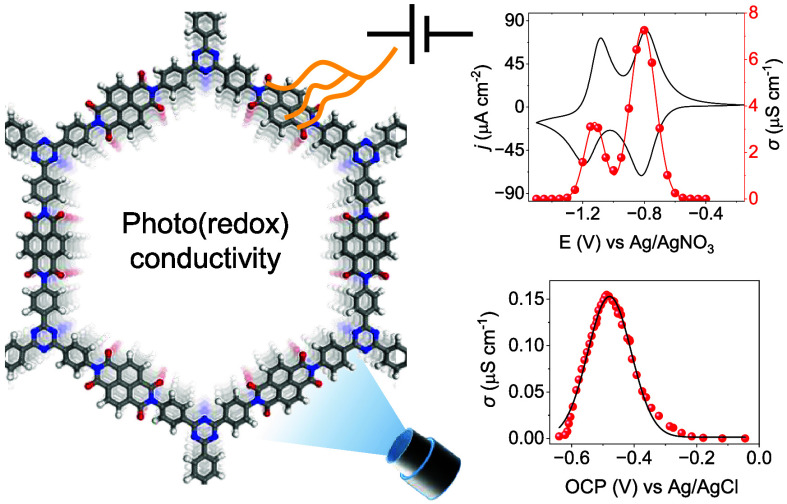

## Introduction

Covalent organic frameworks (COFs), a new class of crystalline porous solids, have emerged as multifunctional materials for energy applications, thanks to their molecular level tunability and large structural diversity.^1–5^ Their ability to incorporate redox active units in the framework with subnanometer spatial precision has been exploited extensively for electrochemical energy storage devices, where the simultaneous transport of electrons and ions is a key prerequisite.^6–9^ Such mixed electronic-ionic conductivity in COFs has been limited by the generally low electronic conductivity of COFs (typically on the order of 10^–9^–10^–5^ S cm^–1^), which has been identified as a major roadblock to applications ranging from batteries to electrocatalysis.^10–13^ Conductivity in COFs is typically rationalized by band-like charge transport or by the redox hopping mechanism.^6^ Band transport is a consequence of effective orbital overlap and in- or out-of-plane charge carrier delocalization through π–π interactions.^14,15^ Recently, π-conjugation has been realized in all three dimensions in a fully conjugated 3D COF, resulting in omnidirectional band-like transport.^16^ In the absence of strong electronic coupling through conjugation—which is the default scenario in most COFs—charge transport by redox-hopping can be realized by incorporation of spatially and electronically discrete redox-active linkers.^6,17^ Here, charge transfer can be understood as a random-walk of charges by bimolecular self-exchange of charge carriers between neighboring redox sites (i.e., the reduced and oxidized forms of the linker). Hence, the hopping probability depends on the availability of a neighboring acceptor site, which is maximized at the formal potential of the redox couple where the concentration of the reduced and oxidized linkers, [red] and [ox], is 50:50, as defined by the Nernst equation.^18,19^ Ott and co-workers have reported a characteristic bell-shaped distribution of the redox conductivity as a function of the applied potential in Zn­(pyrazol-NDI) and Zn­(pyrazol-NDI)_*x*_(pyrazol-PMDI)_*y*_ MOFs, which proceeds through thermally activated redox-hopping showing Arrhenius-type behavior.^18,20^

While redox-active COFs have been widely used as electrode materials in reversible energy storage, especially in secondary ion batteries and supercapacitors, an in-depth understanding of their redox-conductive behavior remains elusive.^21–24^ In addition, little is known about the impact of light on redox conductivity, a knowledge gap potentially vital for the application of COFs in (photo)­electrocatalysis. To obtain a holistic picture of redox conductivity in COFs and the interplay between electronic and ionic partial conductivities, examining the influence of various contributing factors, such as the interplay of ion type, solvent, and electrolyte, is key to the understanding of redox charge transport in COFs.

Herein, we present a comprehensive study analyzing the impact of ions and solvents on the redox hopping mediated charge transport in COFs, and show that it can be modulated by both electrochemical and photochemical stimuli. Using different electrochemical regimes and analytical probes, we determine the apparent electron diffusion coefficients (*D*_e_^app^) and compare them for different solvent-electrolyte combinations in potential-step chronoamperometry experiments. Using electrochemical impedance spectroscopy (EIS), we investigate the evolution of redox conductivity both under an applied bias and illumination, demonstrating bell-shaped redox conductivity and insulator–semiconductor switching behavior in (photo)­redox-active COFs for the first time. Our study thus contributes to the mechanistic understanding of ion-coupled electron transfer in COFs and serves as a framework for the development of (photo)­redox conductive COFs for energy applications and the emerging field of optoionics.

## Result and Discussion

### Synthesis and Characterization

We selected a naphthalene diimide-based redox-active COF (TAPT–NDI COF) where different redox states of the redox-active NDI linker can be accessed at well-defined standard potentials (NDI^0/•–^ and NDI^•–/2–^ by applied potential and NDI^0/•–^ by light), following our recent study on light-induced charge trapping in TAPB-NDI COF.^25^ Imide-linked TAPT-NDI COF was synthesized from 1,3,5-tris­(4-aminophenyl)­triazine (TAPT) and 1,4,5,8-naphthalenetetracarboxylic dianhydride (NTCDA) under solvothermal conditions, according to a modified literature report by Segura^26^ (Figure S1). Fourier transform infrared (FT-IR) spectra of the yellow powdered material shows the disappearance of the N–H or C=O bands of the amino and anhydride monomers, suggesting complete conversion. The formation of a five-membered imide ring was confirmed from the appearance of characteristic bands at 1717 and 1676 cm^–1^ for the asymmetric and symmetric stretching vibrations of the C=O group, respectively, and at 1333 cm^–1^ for the C–N–C stretching vibration (Figure S2). In addition, solid-state ^13^C cross-polarization magic angle spinning NMR (^13^C–CP/MAS NMR) corroborates this finding. A characteristic carbonyl carbon signal at δ = 161.86 ppm and overlapping signals of naphthalenyl and phenyl moieties at δ = 136.61 and 128.09 ppm, respectively correspond to the formation of the five-membered imide ring (Figure S3). The signal at 170.45 ppm was assigned to the triazine unit of TAPT-NDI COF.^27^

Powder X-ray diffraction (PXRD) analysis indicates moderate crystallinity of TAPT-NDI COF with reflections at 2θ = 2.7, 4.8/5.4, and 7.2° indexed as 100, 110/200, and 210 (space group *P*3̅1*m*), and a broad stacking reflection 00*l* at 2θ = 24.2°. Pawley refinement returns a trigonal unit cell (*P*3̅1*m*) with *a* = *b* = 37.246 Å, *c* = 3.651 Å, α = β = 90° and γ = 120°, with refinement results of *R*_wp_ = 1.41% and *R*_p_ = 1.11% (Figure 1a). An eclipsed stacked model of TAPT-NDI COF and the corresponding molecular fragment are presented in Figure 1b. Nitrogen gas sorption isotherms reveal a Brunauer–Emmett–Teller (BET) surface area of 1090 m^2^ g^–1^ and a total pore volume of 0.808 cm^3^ g^–1^ (Figure S4). The pore diameter of 3.1 nm is in line with the calculated pore size distribution (Figures S4–S5). Scanning electron and transmission electron microscopy (SEM/TEM) images show polycrystalline, flaky microcrystals with particle sizes ranging between 50 and 300 nm (Figures S6–S7).

**Figure 1:**
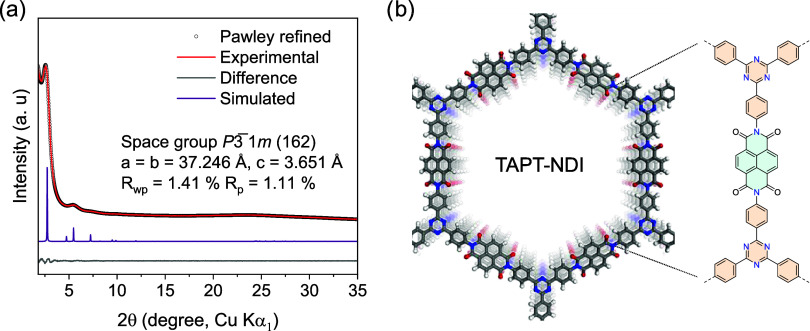
(a) PXRD patterns of TAPT-NDI COF are shown with the experimental data in red, Pawley refined data as black circles, the calculated trigonal structure based on (near eclipsed) AA stacking in violet, and the difference plot (observed minus refined profiles) in gray. (b) Simulated structure of TAPT-NDI COF showing π–π stacking of 2D layers. A close-up of a molecular fragment of the TAPT-NDI unit is shown for clarity.

### Redox Activity of TAPT-NDI COF

To assess the redox activity of TAPT-NDI COF, cyclic voltammetry (CV) was performed in a three-electrode setup with the COF on FTO-coated glass as working electrode, an Ag/AgNO_3_ reference electrode, and a platinum counter electrode in 0.1 M KPF_6_ in dry acetonitrile. The installed NDI linkers are expected to show discrete waves for the NDI^0/•–^ and NDI^•–/2–^ redox couples, assigned to diffusional electron hopping charge transfer. This means that electron propagation through the COF occurs via hopping between neighboring NDI linkers in response to a chemical potential gradient, which is coupled to the diffusion—migration of counterions to maintain charge neutrality. Indeed, two well-distinguished reduction peaks are observed at −0.89 and −1.19 V vs Ag/AgNO_3_ for NDI^0/•–^ and NDI^•–/2–^, respectively (Figure 2a). Altering the time scale of the CV experiments by changing the scan rate reveals subtle differences in the spacing and relative intensity of the redox peaks: The latter decreases from higher to lower scan rates (150 to 1 mV s^–1^). In addition, a transition between two kinetic regimes can be distinguished: The semi-infinite regime at higher scan rates, where the peak current is proportional to the square root of ν (“diffusion wave”), and the finite regime at lower scan rates, where a linear dependence of the peak currents on scan rate ν (“surface wave”) is observed. In line with these regimes, the double-logarithmic analysis of the CV data with a linear fit of log­(*i*_p_) versus log­(ν) plot yields a slope of 0.9 at lower scan rates and 0.5 at faster scan rates (Figure 2b).

**Figure 2:**
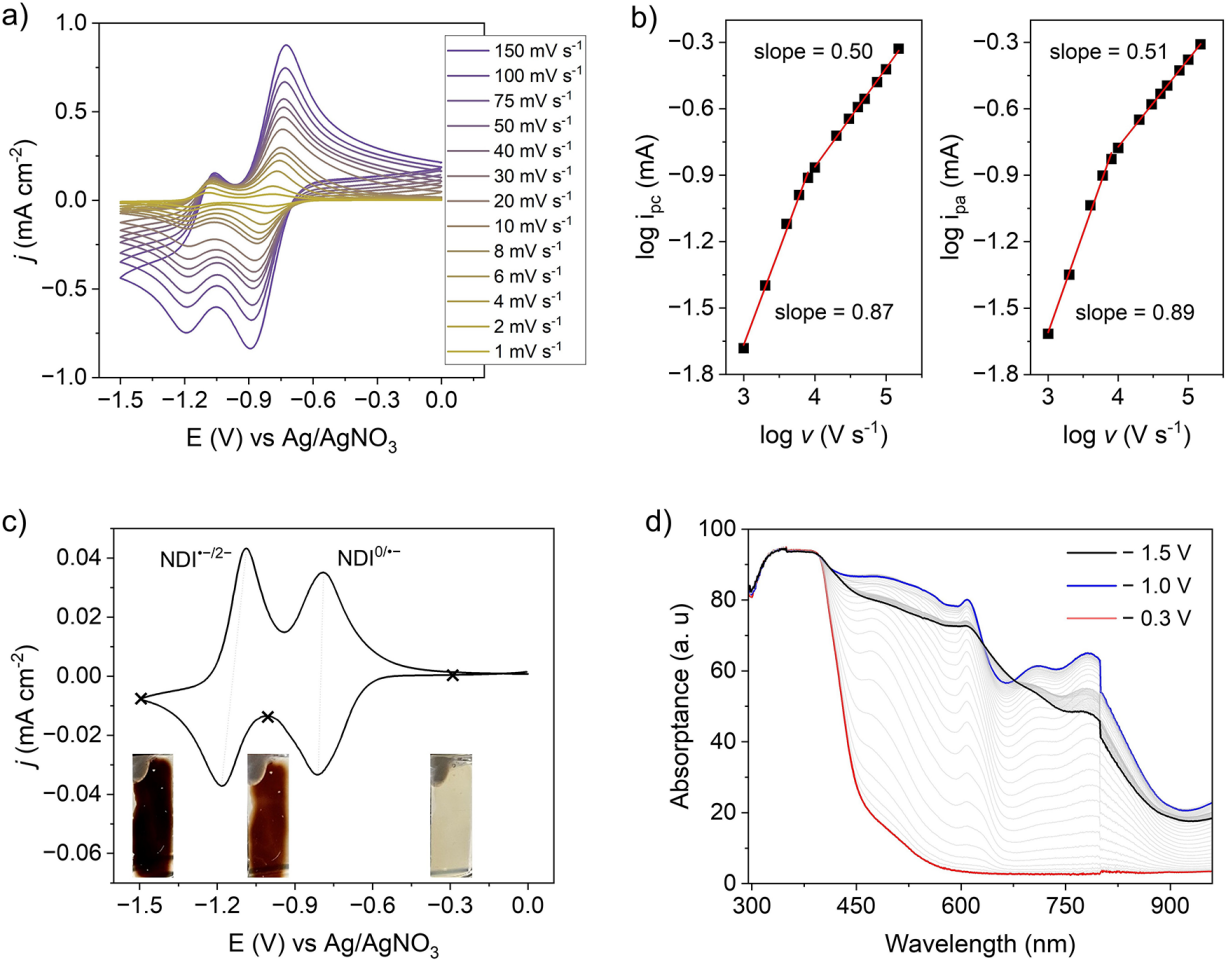
Kinetic analysis of the electrochemical charge storage in TAPT-NDI COF. (a) CV curves of the TAPT-NDI COF on an FTO electrode at various scan rates. (b) Double logarithmic plot of peak current (*i*_pc_ for cathodic and *i*_pa_ for anodic) vs scan rate showing two different limiting regimes. (c) CV curve at a scan rate of 1 mV s^–1^ showing two distinct redox peaks (NDI^0/•–^ and NDI^•–/2–^), and associated color change of the film at marked potentials (−0.3 V for NDI^0^, −1.0 V for NDI^•–^, −1.5 V for NDI^2–^). (d) UV–vis spectroelectrochemical analysis of TAPT-NDI COF, showing the transformation to the NDI^•–^ and NDI^2–^ species.

The crossover between the two regimes signifies different limiting process to be at play, which can be understood based on the different time scales and electron–ion interactions involved: In the semi-infinite regime, the diffusion layer thickness is much smaller than the film thickness and electron hopping through the film is accompanied by the net ingress of ions into the film to maintain charge neutrality (Scheme 1b). As a consequence, ion-coupled electron transfer occurs, which depending on the nature of the ion, solvent and ion-pairing interactions is expected to be highly dependent on the solvent and electrolyte combination.^28^ Therefore, electron transfer diffusion coefficients determined in this regime conflate both electronic and ionic contributions, giving rise to the apparent electron diffusion coefficient *D*_e_^app^.^29^ At lower scan rates, the diffusion layer thickness becomes large enough to experience the finite limit, which is the film thickness. In the steady-state (finite) conditions, the diffusion layer thickness is similar to the film thickness; here, no net ingress of ions from the electrolyte occurs, as ions have infiltrated the film and in- and egress of ions is in equilibrium (Scheme 1c). This situation gives access to the steady-state redox conductivity (σ), which is less affected by long-range ion transport into the film. Note, however that the reorganization and short-range diffusion of ions in the COF channels can still lead to pronounced sensitivity to the ion–solvent combination. The transition between the two limiting regimes is clearly seen in the scan rate-dependent CVs, which can be used to calculate *D*_e_^app^ as will be discussed in more detail below. Similar scan rate dependent switching has previously been observed in MOF materials with varying degrees of redox active linkers.^30,31^

**Scheme 1:**
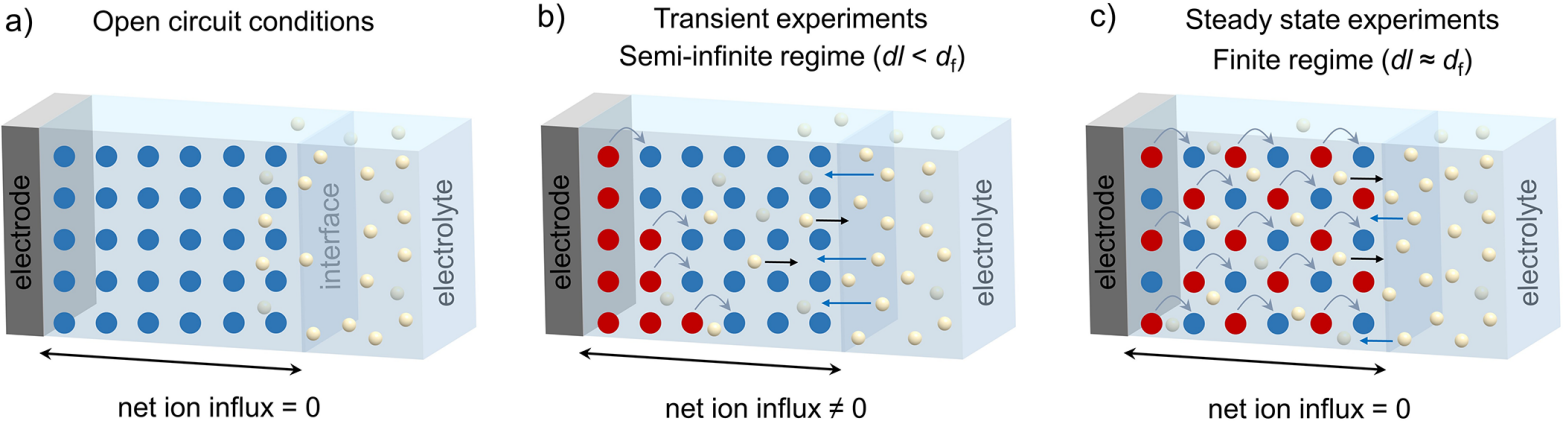
Schematic Representation of TAPT-NDI COF@FTO Electrode and Electrolyte Under Different Electrochemical Conditions.

(a) Under open circuit conditions, without applied bias or light illumination, ions diffuse across the electrolyte-electrode interface, with zero net ion influx. (b) Semi-infinite regime (or large film thicknesses): The diffusion layer thickness (*dl*) is smaller compared to the film thickness, *d*_f_. Electrons diffuse from the electrode/electrolyte interface by bimolecular self-exchange between reduced linker (red) and neutral (oxidized) linker (blue), and the net ion influx is non-zero due to diffusion–migration of counter ions (beige) across the film. The semi-infinite regime is captured by transient electrochemical experiments (potential-step chronoamperometry, chronocoulometry, chronoabsorptometry), which are used for the calculation of apparent diffusion coefficients (*D*_e_^app^). In addition, scan rate-dependent cyclic voltammetry measurements can be used to transition between the semi-infinite regime (faster scan rates), and finite regime (slower scan rates) by altering the time scale of the measurement. (c) Steady-state conditions: An example of 50:50 distribution of reduced (red) and oxidized (blue) NDI units after applying the midpoint potential *E*_0_ for 120 s. Unlike transient experiments, here, the in- and egress of ions is in equilibrium and the net influx of ions is zero. The steady-state situation is used in electrochemical impedance spectroscopy measurements for the calculation of redox conductivity (σ).

As noted from the well-separated redox peaks (NDI^0/•–^ and NDI^•–/2–^), the redox state of the film can be easily modulated electrochemically, which is confirmed by operando UV–Vis spectroelectrochemical measurements (Figure 2c,d). Characteristic π–π* excitations of the NDI core were observed at 341 and 384 nm (down to a potential of −0.3 V vs Ag/AgNO_3_) at the neutral state of the film. During a cathodic scan approaching the NDI^0/•–^ redox couple (−0.81 V vs Ag/AgNO_3_), new absorption bands gradually appeared at 482, 550, 610, 708, and 782 nm, accompanied by a color change from yellow to light brown, which is ascribed to NDI^•–^ radical species.^32–34^ When the potential reaches beyond the NDI^•–/2–^ redox couple (−1.18 V vs Ag/AgNO_3_), the peak intensities at 482, 708, and 782 nm decrease, which is assigned to the formation of NDI^2–^ after the completion of the second reduction, as indicated by a dark brown color. The interconversion between different redox states of NDI units in the cathodic scan can be reversed during the anodic scan (Figure S8), corroborating the reversible two-step electrochemical reduction, accompanied by visible changes in the color of the film.

### Role of Electrolytes and Solvents

As mentioned above, the ion-coupled electron hopping process is highly dependent on the nature of counterions and solvent conditions, which can cause a pronounced shift in the half-wave potentials *E*_1/2_ of the NDI^0/•–^ and NDI^•–/2–^ redox couples and, hence, their peak positions in the CV. In addition to the nature of counterions, solvent properties such as the dielectric constant (ε_r_) and donor ability influence the magnitude of these shifts.^28^ To examine the influence of these parameters in more detail, we compare the CV curves collected in different electrolyte/solvent systems. The differences for different types of ions are most significant when using acetonitrile as the solvent. Two well-defined and distinct redox features for the NDI^0/•–^ and NDI^•–/2–^ couples are observed for both Na^+^ and K^+^ (Figure 3a), characterized by higher current densities and peak-to-peak separations (240 mV for Na^+^, 340 mV for K^+^). While the half-wave potential *E*_1/2_ of the NDI^0/•–^ couple in Li^+^ is similar to that of K^+^, the NDI^•–/2–^ couple shifts positively by 250 mV, i.e., closer to the NDI^0/•–^ couple, resulting in a broad peak at −0.87 V vs Ag/AgNO_3_. This shift likely points to a stronger ion pairing effect of Li^+^ cations with the NDI^•–^ state of the COF film, reminiscent of a fast chemical reaction subsequent to electron transfer. Ion pairing stabilizes the electron accepting orbitals, thereby facilitating the subsequent reductions, and this effect decreases for Na^+^ and K^+^, indicated by the increase in the peak-to-peak separations. In the case of DMF (Figure 3b), the current density as well as the peak-to-peak separations of the redox couples gradually increase in the order Li^+^ (300 mV) < Na^+^ (370 mV) < K^+^ (420 mV). The peak positions and peak-to-peak separation of the redox couples are identical for Li^+^ and Na^+^ in ethanol, despite a higher current density for the latter (Figure 3c). Comparing the CV curves for LiClO_4_ supporting electrolyte in different solvents (Figure 3d), a gradual decrease in the peak-to-peak separations is observed in the order DMF (300 mV) > EtOH (230 mV) > MeCN (50 mV), while that for Na^+^ (Figure 3e) is (370 mV) > MeCN (240 mV) > EtOH (230 mV).

**Figure 3:**
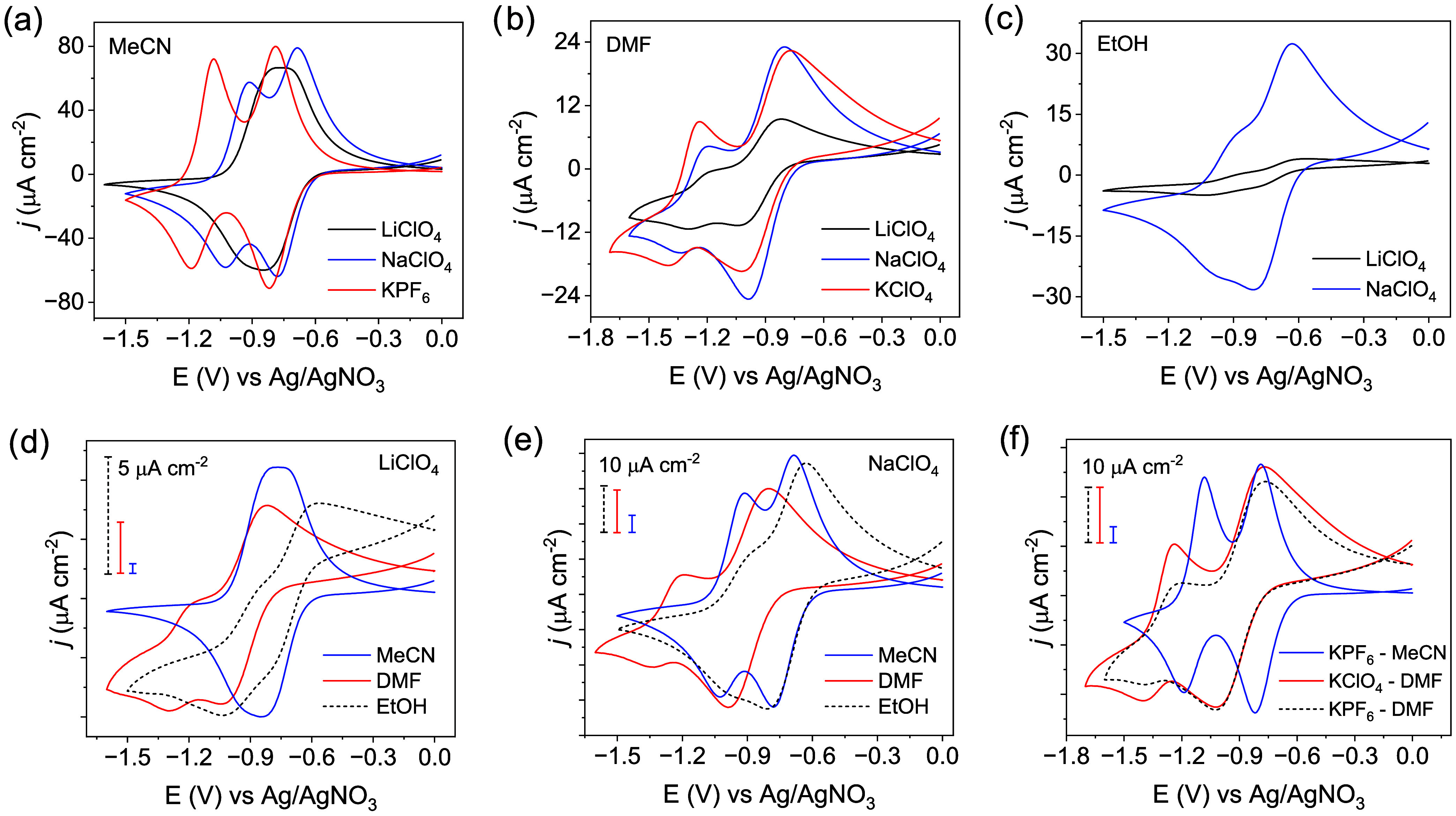
Representative CV curves of a TAPT-NDI COF film under different electrolyte–solvent conditions. Comparison of CV curves measured in (a) MeCN, (b) DMF, (c) EtOH solvents with the supporting electrolytes (LiClO_4_, NaClO_4_, KClO_4_ or KPF_6_). The changes in the redox behavior of the COF for different alkali metal ions (d) Li^+^, (e) Na^+^, (f) K^+^ in different organic solvents (MeCN, DMF, EtOH). CV conditions: 0.1 M supporting electrolyte in the chosen solvent; scan rate: 2 mV s^–1^.

Apart from net cation influx into the film, charge compensation could also occur via net anion efflux upon electron injection, in which anion size might influence current density and peak positions. To probe counteranion effects, we compared CVs in DMF using KPF_6_ and KClO_4_ electrolytes. We observed only a slightly higher current density and a marginally more negative half-wave potential for the NDI^•–/2–^ couple with ClO_4_^–^ (*E*_1/2_ = – 1.32 V vs Ag/AgNO_3_) relative to PF_6_^–^ (−1.30 V). These small differences suggest that charge neutralization of NDI radicals is dominated by countercation influx, with minimal anion-specific effects (Figure 3f). Additionally, in water all alkali metal ions show a broad peak centered at −0.58 V vs Ag/AgCl due to two indiscernible redox peaks.^25^ The key data from the CV measurements for the nonaqueous electrolytes (referenced to Ag/AgNO_3_) are summarized in Table 1. Water is excluded because those measurements were referenced to Ag/AgCl; more details can be found in Figures S8–S31. To summarize, the current response and the peak position of the redox couple is highly dependent on the counterion and solvent. This behavior suggests that the diffusional transport of ions varies due to the interplay between ion pairing, solvent polarity, and donor number (Table S1), which will be detailed in the following.

**Table 1:** *E*_1/2_ for the NDI Redox Couples of TAPT-NDI COF@FTO in Different Electrolyte-Solvent Conditions^a^

	MeCN		DMF		EtOH	
electrolyte	*E*_1/2_^0/•–^ (V)	*E*_1/2_^•–/2–^ (V)	*E*_1/2_^0/•–^ (V)	*E*_1/2_^•–/2–^ (V)	*E*_1/2_^0/•–^ (V)	*E*_1/2_^•–/2–^ (V)
LiClO_4_	–0.83	–0.88	–0.93	–1.23	–0.71	–0.94
NaClO_4_	–0.72	–0.96	–0.90	–1.27	–0.71	–0.94
KPF_6_	–0.79	–1.13	–0.89	–1.30		
KClO_4_			–0.89	–1.32		

a Conditions: 0.1 M supporting electrolyte in solvents; scan rate: 2 mV s^–1^; potentials referenced to Ag/AgNO_3_.

The NDI linkers in the TAPT-NDI COF@FTO are electronically isolated, and electron transport occurs via a hopping mechanism, which is coupled with the movement of charge-balancing counterions (Scheme 1).

### Determination of Apparent Diffusion Coefficient, *D*_e_^app^

Since the cation-coupled electron hopping process is diffusional in nature, the effective rate of charge propagation through the film by electron self-exchange between neighboring redox sites can be quantified by the apparent electron diffusion coefficient, *D*_e_^app^. The annotation “apparent” reflects the coupling between electron motion and counterion transport through the pores, and hence is distinct from the intrinsic electron hopping rate.^20,28,35,36^ To determine *D*_e_^app^, we use potential step chronoamperometry and monitor the initial, transient current response where the diffusion layer is much smaller than the film thicknesses (semi-infinite regime, Scheme 1b). First, to ensure the neutral state of all NDI linkers, a potential is applied in the non-faradaic region for 120 s, followed by stepping the potential to a suitable negative value to reduce the linkers to the NDI^•–^ radical anion. The potential was chosen from the CV curves at a potential higher than the NDI^0/•–^ peak. The target potential was held for 900 s ensuring quantitative radical anion formation, and the time-dependent current response was used for the quantification of electrochemically accessible NDI linkers. Finally, the molar concentration of associated electroactive species, C_0_ (mol cm^–3^), was estimated. All equations and parameters used for the calculation are given in the supporting information, Figures S33–S44, Tables S2–S12.

Cottrell plots were used to calculate the *D*_e_^app^ values in the semi-infinite diffusion regime, where the time-dependent current density *j*(*t*), is linear to √*t* for short transients. The slope in that regime can be used to calculate *D*_e_^app^ (details in Supporting Information). Figure 4 shows a representative Cottrell plot from a chronoamperometry experiment of a COF film (thickness ∼ 1 μm) using 0.1 M KPF_6_ in acetonitrile electrolyte and short transients (0.5–7 s). The average diffusion coefficients *D*_e_^app^ for all the counterions in different solvents are plotted in Figure 4, and other important parameters are tabulated in Tables S2–S12. The molar concentrations of electroactive species are on the same order of magnitude (10^–8^ mol cm^–2^), irrespective of the thickness of the film and counterion/solvent conditions. However, the diffusion coefficients vary by up to an order of magnitude in different solvents and consistently follow the trend Li^+^ < Na^+^ < K^+^ (Figure 4c). In addition, *D*_e_^app^ values were calculated from the transition scan rates in CV experiments and also fall in the range of 10^–10^ to 10^–13^ cm^2^ s^–1^ (Figure S9–S32).

**Figure 4:**
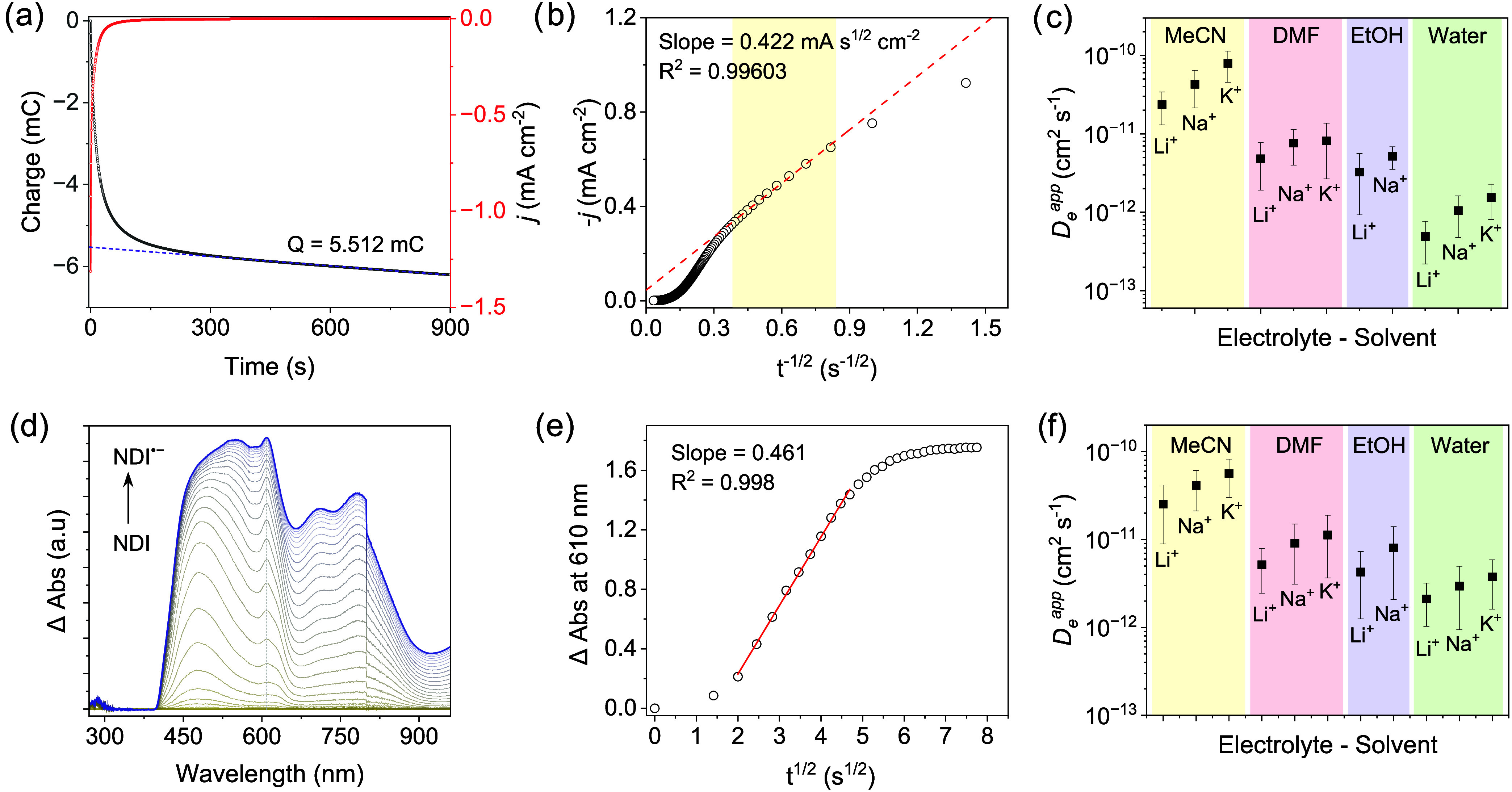
Electrochemical analysis for the estimation of diffusion coefficients. (a) Representative chronoamperometry (red) and chronocoulometry (gray) curves of a COF film measured in 0.1 M KPF_6_ in MeCN after a potential step from −0.3 V → −1 V vs Ag/AgNO_3_ (selected from CV) to convert NDI^0^ to NDI^•–^. The total charge passed after complete reduction was estimated after subtracting a residual background current (blue dashed line). (b) Corresponding Cottrell plot showing the linear fit (selected points in the yellow box) from ∼1.6 to 6.4 s after the potential step used to extract *D*_*e*_^app^ from eq S3. (c) Average *D*_*e*_^app^ estimated under different electrolyte-solvent conditions. (d) Change in absorbance of NDI^•–^ in a step-potential chronoamperometry. (e) Cottrell fit of the kinetic absorbance response at 610 nm used to extract *D*_*e*_^app^ from eq S4. (f) Average *D*_*e*_^app^ estimated under different electrolyte-solvent conditions.

To further verify this trend, *D*_e_^app^ values were independently determined by monitoring the absorbance change of NDI^•–^ at 610 nm in a kinetic potential-step chronoamperometry experiment by stepping the potential from 0 to −1 V vs Ag/AgNO_3_ (Figures 4d, S45–S55). The modified Cottrell equation was utilized to estimate the *D*_e_^app^ values (Figure 4e), which were systematically higher than those obtained by chronoamperometry, with exceptions especially in MeCN (e.g., Na^+^ and K^+^). Overall, however, the trends across electrolyte–solvent systems were similar (Figure 4f, Table S13). It is important to note that the discrepancy in the *D*_e_^app^ values is more severe in thicker films (>5 μm), as the geometrically calculated amount of redox active species accounts for the case that all redox centers are electrochemically addressable, which is unlikely in the semi-infinite diffusional regime, i.e., at high ratios of film thickness to diffusion layer thickness. It can therefore be inferred that only a small % of the film participates in the redox process. Because film thickness strongly affects *D*_e_^app^ accuracy, we optimized the thickness to maximize electrochemical accessibility. We then verified *D*_e_^app^ across four independent electrodes with film thicknesses between 800–1300 nm, where 77–89% of the film was electrochemically addressable (Figures S33–S44; Tables S2–S12).

Now, focusing on the *D*_e_^app^ values, the polarity and donor number of the solvents seem to play a significant role in dictating the diffusion of ions as the diffusivities increase along the series water < ethanol < DMF. Interestingly, the *D*_e_^app^ values are one order of magnitude higher in MeCN. This difference is possibly due to the size of the solvation shell in MeCN, which is smaller than in DMF.^37,38^ A solvent and ion-dependent study in redox active MOFs has shown that in a high dielectric electrolyte where ion pairing is less significant due to more efficient electrostatic screening, the size of the ions has a dominant effect on *D*_e_^app^.^28^ Thus, the interplay between several factors such as ion pairing, solvation effects, effective ionic radius, and dielectric constant of the solvent can influence the diffusional migration of ions. The effective size of solvated cations governs both their ion pairing with the reduced linker and their arrangement within the COF pores, thereby affecting charge transport. In less polar solvents (e.g., MeCN), ion pairing is expected to be stronger: small cations with high charge density (Li^+^) form tighter ion pairs with the reduced linker, which suppresses interlinker electron self-exchange and lowers the apparent diffusion coefficient (*D*_e_^app^) relative to larger cations (Na^+^, K^+^), reflected in the experimental *D*_e_^app^ values. In water, extensive hydration reduces effective charge density and weakens ion pairing; on the other hand, it increases the effective radius of the hydrated ions, thus slowing down cation movement. Therefore, in this regime, charge transport is expected to vary inversely with the cation’s hydrodynamic size (Li^+^ > Na^+^ > K^+^), i.e., *D*_e_^app^, K^+^ > Na^+^ > Li^+^, which is what we observe in our experiments.

### Redox Conductivity and Semiconductor Switching

As discussed above, the transient decay of the current response encodes charge transport through electron hopping between discrete molecular sites, coupled with ion migration to maintain charge neutrality. At this point, two aspects should be emphasized: First, distinguishing between the intrinsic electron diffusion coefficient *D*_e_ and the apparent diffusion coefficient *D*_e_^app^ is crucial to account for the importance of *coupled* electron-ion migration under nonsteady-state conditions. As such, the current response depends both on the intrinsic electron hopping diffusion coefficient and the diffusivities of the counterions, which can be coupled in complex ways. Second, ion diffusion–migration effects should primarily be relevant in the initial stages of applying a potential step, whereas at steady-state (i.e., after the film has been reduced by a given extent defined by the applied equilibrium potential) the net counterion flux into the film should be zero, and the measured current response should therefore be largely independent of the mobile ion diffusivity (see Scheme 1). Measurements of the steady-state redox conductivity of the film should thus more accurately reflect the intrinsic electron hopping, with only limited interfering diffusion–migration effects of the counterions. Note, however, that even though the in- and outflux of ions into and out of the film is net zero, microscopic effects such as ion pairing and size effects can still affect the intrinsic electron diffusion rate.^18,39,40^

To understand the redox conductivity under steady-state conditions, it is important to relate the macroscopically observed conductivity to its microscopic mechanism. In general, redox conductivity should be a function of the redox state of the COF film: As Ott and co-workers have shown, the electron hopping process is facilitated by the availability of neighboring acceptor sites, as electron self-exchange inherently is a bimolecular process. Therefore, a maximum value for the redox-conductivity is expected at an equal ratio of the oxidized and reduced sites, i.e., at the formal potential of the redox couple.^18,20^ To assess the redox conductivity of the COF film, different potentials were applied for 120 s in a cathodic scan to adjust the desired redox state. Under these conditions, the film is expected to be in the finite diffusion regime, where the time scale of the experiment is slower than the diffusional electron hopping charge transport, resulting in the progression of the reduction front across the entire film. Electrochemical impedance spectroscopy (EIS) was used over a frequency range of 0.1–10,000 Hz with a potential modulation of 10 mV to probe the redox conductivity at near steady-state conditions.

Representative Bode plots of experimental impedance data as a function of applied potential map the redox state (*x*-TAPT-NDI, 0.0 ≤ *x* ≤ 2.0, where *x* is the mole fraction of electron reduction, *x* = 0 for NDI^0^, *x* = 1 for NDI^•–^ and *x* = 2 for NDI^2–^; note that the mole fraction refers to the idealized situation where the entire film is electrochemically addressable) of the film (Figures 5a–c, S56–S64). Of note, the progression of the impedance over the applied potentials closely mirrors the respective CVs, with the minimum impedance appearing around the formal potentials of the redox couples involved. Quantification of the redox conductivities was done by constructing an equivalent circuit to fit the experimental data, approximating purely electronic transport (Figures S65–S66, Tables S14–S17). The potential-dependent redox conductivity evolves as either two well-separated bell-shaped curves or a merged bell-shaped distribution depending on the electrolyte/solvent combination. In the neutral state, or after the complete two-electron reduction (applied potential beyond the NDI^•–/2–^ couple) the redox conductivities are in the range of 10^–10^ to 10^–9^ S cm^–1^, suggesting insulating behavior. Between these extremes, i.e., at applied potentials where the COF film adopts mixed redox states, two bell-shaped curves span over the representative potential range, reaching conductivities of up to ∼10^–6^ S cm^–1^ at the respective formal potentials of the NDI^0/•–^ (*x* = 0.5) and NDI^•–/2–^ (*x* = 1.5) redox couples (Figures 5d–f, S67–S72). The redox conductivity of the film is thus by a factor 10^4^ higher than that of the neutral or fully reduced states. A similar redox switching behavior between insulating (∼10^–10^ S cm^–1^) and semiconducting behavior (∼10^–6^ S cm^–1^) has previously been reported for MOFs,^18,20^ but remains elusive for COFs so far. The fact that the redox conductivity is potential dependent and can be varied by orders of magnitude holds promise for the application of COFs in logic and nanoelectronic devices.

**Figure 5:**
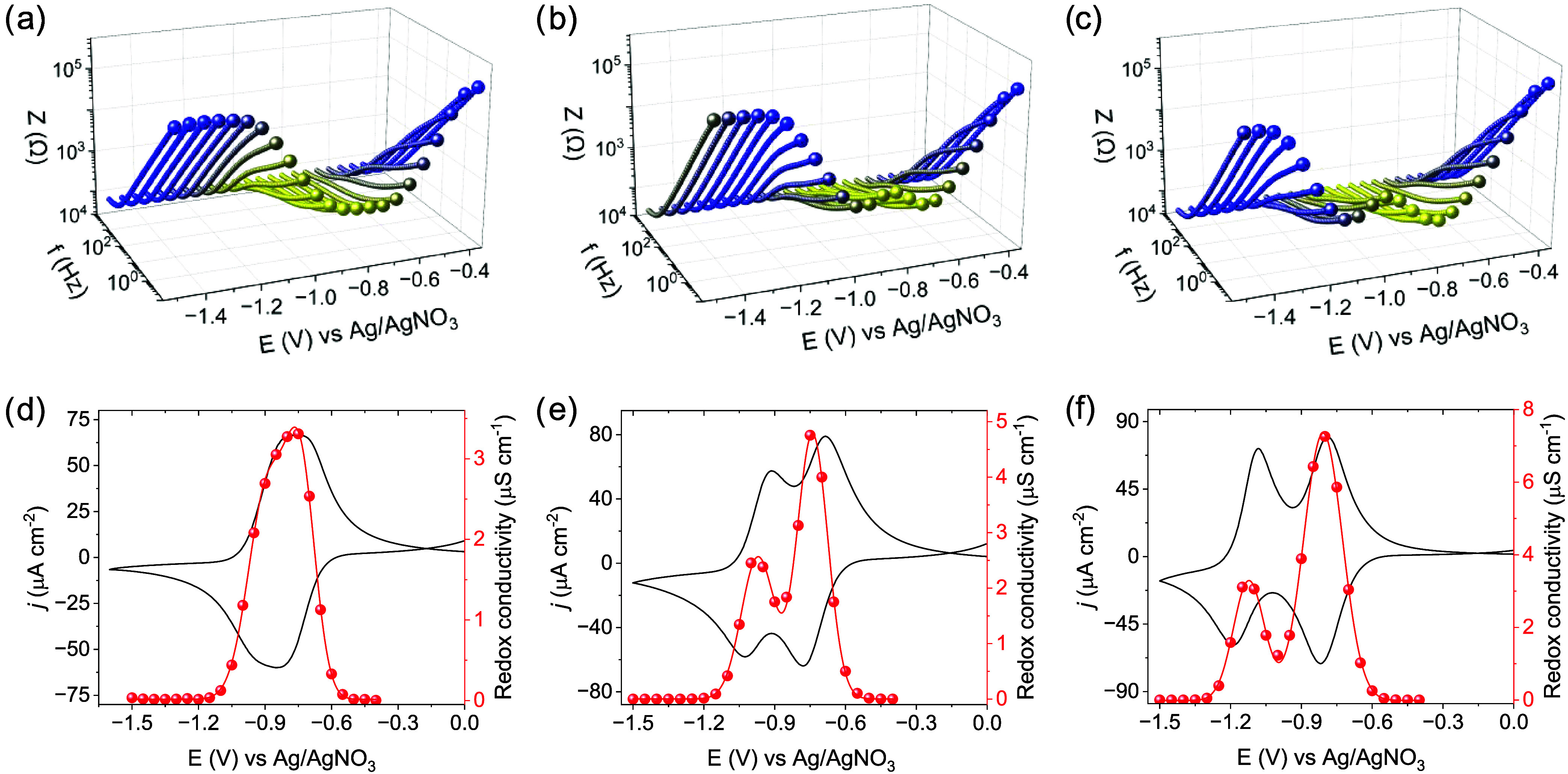
Representative Bode plots of TAPT-NDI COF in MeCN for (a) Li^+^, (b) Na^+^, (c) K^+^ electrolytes at different applied potentials (the impedance data point at the frequency of 0.1 Hz was magnified for each measurement to highlight the effect of the redox state). Evolution of the steady-state thin-film conductivity as a function of the applied electrochemical potential, which determines the mole fraction of electron reduction, *x*-TAPT-NDI, 0.0 ≤ *x* ≤ 2.0 (where *x* = 0 for NDI^0^, *x* = 1 for NDI^•–^ and *x* = 2 for NDI^2–^) in MeCN for (d) Li^+^, (e) Na^+^, (f) K^+^ electrolytes. A Gaussian fit was performed for both NDI^0/•–^ and NDI^•–/2–^ bell-shaped redox conductivities.

Notably, the COF exhibits excellent insulator/semiconductor switchability between *x* = 0.0 and *x* = 0.5 over 100 cycles (Figure 6a), with only minimum changes in the electrochemical features observed after a long-term stability test (Figure S73). The characteristic evolution of a bell-shaped redox conductivity is inherently governed by the bimolecular nature of the electron self-exchange, which requires a donor–acceptor couple in direct vicinity. Although equivalent circuit models accounting for purely electronic contributions result in a satisfactory fit, the fit was further improved by introducing an additional constant phase element (CPE). While not sufficient for proving the role of cations in the redox conductivity, the improved fit is consistent with ion-coupled electron transport to be at play also under steady-state conditions, with electronic and ionic contributions to the total conductivity, as also supported by bell-shaped features of the CPE coefficients (Figure S74). Indeed, the redox conductivity and switching behavior are highly dependent on the electrolyte-solvent combination. By exchanging MeCN as the solvent, the redox conductivity values drop by 1 order of magnitude for DMF, EtOH and H_2_O for all alkali electrolytes. The redox conductivity values follow the trend Li^+^ < Na^+^ < K^+^ in all solvents, except in DMF, where the electrolytes exhibit similar values (Figure 6b; the respective redox conductivity switching experiments can be found in Figures S67–S72). Different degrees of solvation of the ions in the different solvents and, hence, dielectric screening and size effects could modulate electron-ion interactions, leading to more or less strong ion pairing effects, thereby influencing the redox conductivity.

**Figure 6:**
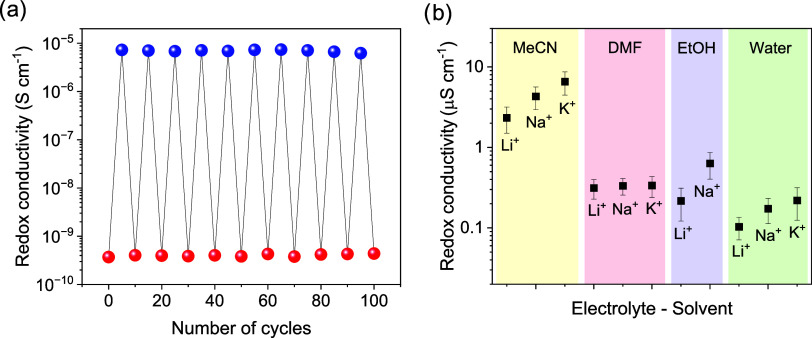
(a) Switching behavior of the redox conductivity between insulating (bottom sphere) and semiconducting (top sphere) over 100 cycles. (b) Maximum steady-state conductivity corresponding to the NDI^0/•–^ redox couple for different electrolyte-solvent conditions.

### Photochemically Induced Redox Conductivity

The redox conductivity described above requires an applied electrochemical potential, which is equivalent to the injection of charge carriers into the film until the NDI units have adopted a certain average (mixed or uniform) valence state. While the electrochemical method is convenient, the optical activity of the NDI units allows for an alternative pathway to modulate redox conductivity: light. Photochemically triggered redox conductivity is based on above-bandgap excitation, creating electron hole-pairs. Upon addition of a donor (acceptor), holes (electrons) are extracted from the film, resulting in a net negative (positive) charge of the film. Note that this process is distinct from photocurrent generation, as the photoredox system involves an irreversible chemical reaction consuming hole (electrons), hence resembling a source-drain system that generates a steady-state current. In a previous report, we have explored the long-term charge stabilization of photoexcited electrons in a similar NDI-COF (TAPB-NDI COF) for solar battery applications, which already points to the feasibility of this approach.^25^

To test whether the redox state of the film can be adjusted photochemically, the TAPT-NDI COF film was irradiated above the bandgap (*E*_g_ = 2.89 eV, Figure S75) in O_2_-free aqueous electrolyte in the presence of 4-methylbenzyl alcohol (4-MBA) as a sacrificial electron donor (SED). Note that 4-MBA is small enough to penetrate the COF pores and diffuse into the film. The charging process was followed by spectroelectrochemistry: The neutral NDI-COF film showed vibronic progression (0–1 band around 337 nm and 0–0 band around 368 nm) as generally observed in monomeric NDI derivatives. Upon 365 nm UV illumination (nominal power *P*_nominal_ ≈ 100 mW cm^–2^), the light-yellow color of the film gradually turned brown, accompanied by the evolution of new excitations at 490, 545, 618, 688, and 760 nm, indicative of NDI^•–^ radical formation (Figure 7a). The radical signal reached a stationary state after 10 min illumination with an isosbestic point at 382 nm and remained stable thereafter. Note that whereas the electrochemical method allows access to both reduced species (NDI^•–^ and NDI^2–^), the photochemical method produces only the one-electron reduced NDI^•–^ radical species. The photogenerated electrons remain trapped in the form of NDI^•–^ radicals after hole quenching by the SED. This behavior is in fact advantageous as it allows us to focus on the NDI/NDI^•–^ couple in an aqueous medium, which is harder to probe electrochemically due to the coalescence of the two redox waves (*vide supra*).

**Figure 7:**
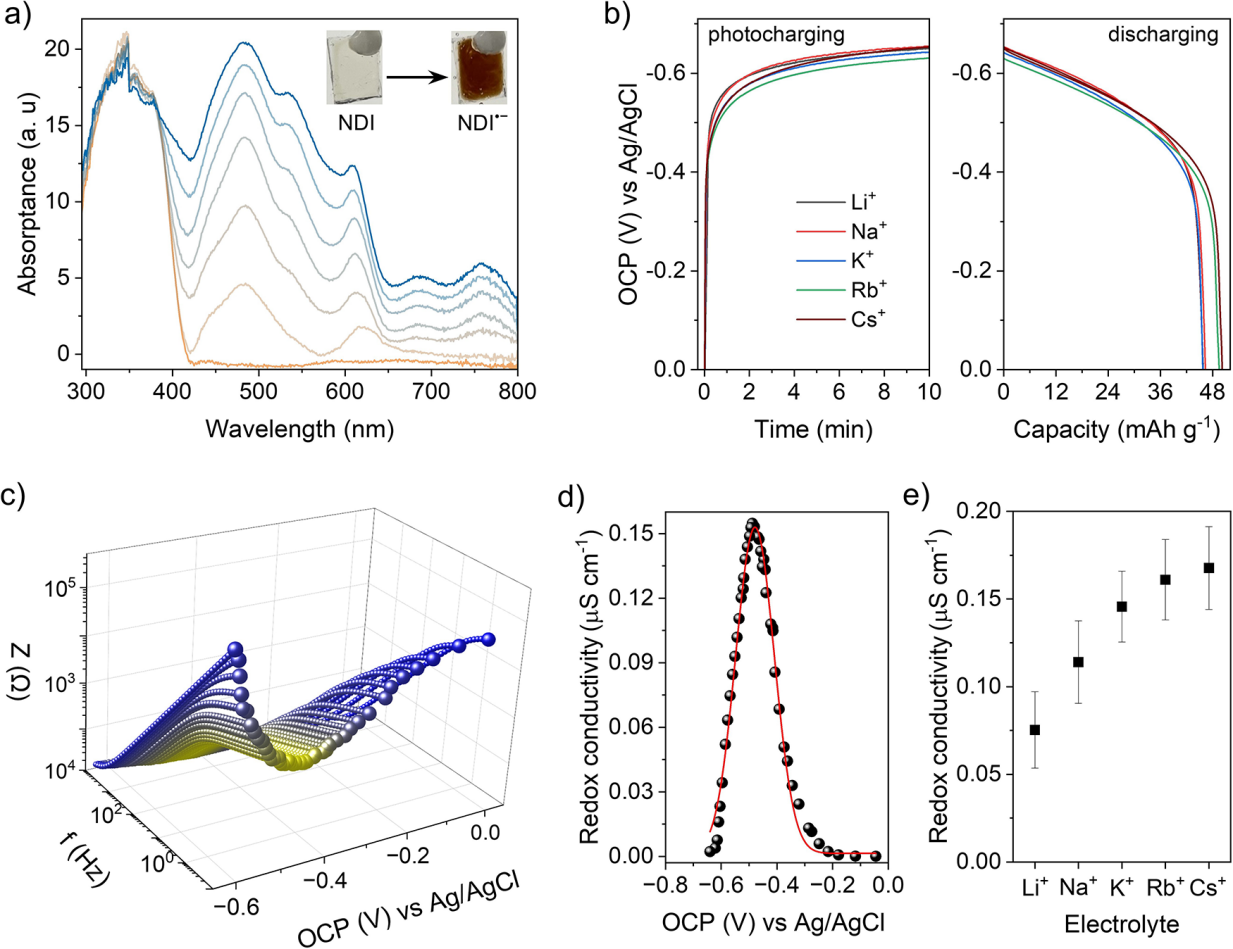
(a) UV–vis absorption spectra of the TAPT-NDI COF film on FTO showing the formation of the NDI^•–^ radical anion upon continuous illumination. (b) Photocharging of the COF photoanode in different electrolytes at 365 nm UV illumination (10 min) and under OCP conditions. Respective electric discharge curves in the dark with a fixed current density of 15 mA g^–1^ (normalized against mass of COF). (c) Representative Bode plot of TAPT-NDI COF in KCl electrolyte at different open circuit potentials (the impedance data point at the frequency of 0.1 Hz was magnified for each measurement to highlight the effect of redox state). (d) Evolution of the steady-state thin-film conductivity in KCl electrolyte as a function of photoinduced open circuit potential. (e) Comparison of photoinduced steady-state conductivity in different supporting electrolytes. All (photo)­electrochemistry measurements were performed in O_2_-free aqueous 4-MBA as SED with supporting electrolytes.

We monitored the accessible open circuit potential in a three-electrode setup by irradiating the FTO electrode from the backside in an O_2_-free electrolyte. Without a dedicated electron donor, the OCP value reaches −0.32 V vs Ag/AgCl. Addition of 4-MBA causes a further rise to −0.63 V vs Ag/AgCl, as the photoelectrons get accumulated in the film after quenching of the photogenerated holes. There were no significant differences in the photo-OCP value or the charge storage capacities in the presence of different counterions (Li^+^, Na^+^, K^+^, Rb^+^, Cs^+^) as depicted in Figure 7b. In all cases, a maximum capacity of 48 mAh g^–1^ (82% of the theoretical capacity) was achieved for 10 min of photoirradiation (finite regime of the film) and subsequent electrical discharging at a current density of 15 mA g^–1^.

Next, to probe the photoinduced redox conductivity in COF, we collected impedance data at numerous irradiation times, which encode different OCP values that in turn translate into different redox states of the film. It must be noted that the redox states (photo-OCP) remain stable during the course of the measurements, thus giving rise to minimal experimental error. With increasing irradiation time, the impedance value gradually decreases until the photo-OCP reaches −0.48 V vs Ag/AgCl, with a slight variation for different counterions (Figures 7c, S76–S79). Prolonged irradiation causes a shift toward more NDI^•–^ species, and the impedance increases again, reaching almost the initial value until the OCP arrives at −0.63 V vs Ag/AgCl, corresponding to *x* = 1.0. The experimental impedance data plotted against the OCP vs Ag/AgCl closely resemble the Bode plot obtained for the electrochemical measurements.

Overall, the redox conductivity values at different OCP vs Ag/AgCl values display the characteristic bell-shaped curve observed also when applying an electrochemical potential, with a maximum in conductivity at −0.48 V vs Ag/AgCl due to the photoreduction of 50% of the film (Figure 7d). Representative Nyquist plots and fitting parameters are shown in Figure S80 and Tables S18–S20. The maximum redox conductivity values range between 0.07 and 0.17 μS cm^–1^ in the presence of different counterions (Figure 7e, S81), which is about an order of magnitude lower compared to the redox conductivity obtained by the electrochemical method, while maintaining the overall trend. We attribute the difference to reduced photochemical accessibility and to limited charge carrier mobility in the film, the latter likely constrained by the bulky electron donor 4-MBA and its oxidation products—factors that are less limiting under electrochemical conditions where charge is injected via the electrode rather than a sacrificial electron donor. Taken together, the above observations point to two novel features of the NDI COF films: (i) Light-induced charge storage, mediated by the optoionic coupling between photogenerated electrons and counterions from solution, which gives rise to distinct and stable redox states of the film. As a consequence, (ii) light-tunable redox conductivity is observed, which opens new perspectives for the use of redox-active COFs as photoresponsive sensors, gates or switches.

## Conclusion

Herein, we have investigated electron hopping diffusion and redox conductivity of TAPT-NDI COF films by installing a suitable redox active linker—NDI—where different redox states can be accessed by electro- and photochemical stimuli. Deriving apparent diffusion coefficients *D*_e_^app^ from potential step chronoamperometry measurements revealed a substantial influence of counterion size, ion pairing and the nature of the solvent on *D*_e_^app^. Steady-state conductivities were probed by electrochemical impedance spectroscopy as a function of applied potential and correlated with distinct redox states of the film. Our measurements reveal a bell-shaped redox conductivity, akin to those observed for redox active MOFs, where the conductivity is highest at the formal potential of the NDI^0^/NDI^•–^ and NDI^•–^/NDI^2–^ redox couples, consistent with the highest probability for bimolecular self-exchange electron hopping between the redox units at these potentials. We further demonstrated tunable redox conductivity using light as stimulus for the first time. Adjusting the redox state of the film by photoredox reactions results in a change in redox conductivities by four orders of magnitude. The remarkably stable switching between insulating (∼10^–10^ S cm^–1^) and semiconducting behavior (∼10^–6^ S cm^–1^) for both photo- and electrochemical stimuli bodes well for applications in sensing or logic elements, such as switchable (photo)­memory devices. Finally, this work opens new perspectives for the use of redox-active COFs in (photo)­electrocatalysis, where the limited conductivity of COFs has been a significant roadblock to their utilization in catalysis so far. Against this backdrop, the findings of this study suggest that the redox conductivity of a COF can be increased by orders of magnitude when operating in a suitable potential regime, entailing potentially dramatic improvements in catalytic activity.

## Supplementary Material



## Notes

Detailed experimental methods, along with corresponding supporting Figures S1–S81 and supporting Tables S1–S20 mentioned in the main text (PDF)
