# Supplementary material for: Ion Diffusion and (Photo)redox Conductivity in a Covalent Organic Framework
Source: J Am Chem Soc. 2026 Jan 15;148(3):3305–15. doi: 10.1021/jacs.5c17763 (PMC12856890; doi:10.1021/jacs.5c17763)
Supplement: Supplementary file 1 [file ja5c17763_si_001.pdf]

Supporting Information

Ion Diffusion and (Photo)redox Conductivity in a Covalent Organic Framework

Bibhuti Bhusan Rath,[1]* and Bettina V. Lotsch[1,2,3]*

[1] Nanochemistry Department, Max Planck Institute for Solid State Research, Heisenbergstraße 1, 70569 Stuttgart, Germany

[2] Department of Chemistry, Ludwig-Maximilians-Universität (LMU), Butenandtstr. 5-13, 81377 Munich, Germany

[3] Cluster or Excellence e-conversion, Lichtenbergstrasse 4a, 85748 Garching, Germany

* Corresponding authors. E-mails: bb.rath@fkf.mpg.de, b.lotsch@fkf.mpg.de

1. Materials and Instrumentation

Materials

Commercially available reagents and solvents were purchased from Sigma-Aldrich, TCI chemicals, Avra chemicals, and used without further purification.

General instrumentations and methods

Supercritical CO_2 ($scCO_2$). After an overnight Soxhlet treatment, Leica EM CPD300 critical point dryer was used for $scCO_2$ rinsing and drying of TAPT-NDI COF. Initially, the sample chamber was half-filled with methanol at 13 °C and the liquid was stirred for 15 min post CO_2 infusion. After around 20 exchange cycles, CO_2 was removed at 40 °C.

Infrared (IR) absorbance spectroscopy. IR absorbance spectra were recorded using PerkinElmer Spectrum Two spectrometer in attenuated total reflection (ATR) geometry equipped with a diamond crystal. Ten measurements were averaged with a resolution of 4 cm^{-1}. Spectral post processing was performed by correcting the ATR effect (contact parameter: 0), subtracting the base line.

Powder X-ray diffraction (PXRD). PXRD patterns were collected using a Stoe Stadi P diffractometer with a Cu $K\alpha_1$ source monochromatized with Ge(111) in a Debye-Scherrer geometry at room temperature (RT). Powder samples sealed in ø1.0 mm glass No. 14 capillaries were measured with spinning.

Solid-state nuclear magnetic resonance (SSNMR) spectroscopy. ^{13}C cross-polarization (CP) magic-angle spinning (MAS) SSNMR spectra were recorded using a Bruker Avance III 400 MHz spectrometer. The measurements were done at a rotation frequency of 20000 Hz, and a contact time (p15) of 3000 μs at a temperature of 293 K. The chemical shifts (δ) were referenced relative to α-glycine (176.5 ppm).

Scanning electron microscopy (SEM). SEM images were recorded on a Zeiss Merlin microscope under the electron high tension voltage of 1.5 kV. The samples were cast on indium-doped tin oxide (ITO) substrates.

Transmission electron microscopy (TEM). TEM images were recorded on a Philips CM 30 ST microscope with a LaB_6 cathode and TVIPS TemCam-F216 CMOS camera operated under 300 kV. The samples were gently ground and contacted with a holey carbon/copper grid.

Gas sorption measurements. Nitrogen sorption measurements were conducted at 77 K using a Quantachrome Instruments Autosorb iQ 3. The pore size distribution (PSD) was determined by employing the N_2 adsorption data at 77 K and applying the QSDFT model (cylindrical pores, adsorption branch) within ASiQwin software version 3.01. Prior to measurement, the TAPT-NDI COF sample was subjected to activation under high vacuum conditions at 120 °C for a duration of 12 hours. For the determination of the BET surface area, a specific pressure range (P/P_0 = 0.14–0.27) was selected.

Ultraviolet–visible (UV–vis) spectroscopy. UV–vis absorptance spectra were recorded on a Cary 60 UV–vis spectrophotometer with a 1 cm cuvette at RT.

UV-vis spectroelectrochemistry was conducted by combining Metrohm Autolab potentiostat (PGSTAT302) with Nova 2.1.4 software electrochemistry setup with the above spectrophotometer. A cuvette was employed as the electrochemical cell, with Pt wire as pseudoreference and all other conditions remained the same as above.

Electrochemical and Photoelectrochemical methods

Electrode preparation. TAPT-NDI COF ink was prepared by vigorously stirring 5 mg of pristine COF in a 9: 3: 1 ratio of DMF: EtOH: Nafion (total volume 10 mL) for 2 h, yielding a dispersion of 0.5 mg/ml. SIGMA ALDRICH FTO coated glass slides (surface resistivity: 7 Ohm/sq) were cut to dimension of 7×6 mm or 11×10 mm, and were thoroughly washed with water and isopropanol followed by O_2 plasma cleaning for 10 minutes. An equivalent of COF ink usually 10 μg was drop cast on the FTO substrates and dried at room temperature. The COF film was also grown on FTO coated glass substrates under solvothermal synthesis conditions for comparison. The COF film was scratched a little at the corner of the substrate and silver paste was used to make contact with isolated copper wire. The contact was properly sealed with epoxy (3 M Scotch-Weld DP410), leaving an exposed electrode area of approximately 5×5 mm^2 or 7×7 mm^2.

Electrochemical measurements. All the electrochemical measurements were performed in a custom-made closed glass reactor equipped with a quartz window for illumination. A conventional three-electrode setup with either $Ag/AgNO_3$ (for organic solvents) or Ag/AgCl (saturated KCl, RE-1CP, for aqueous solvents) as reference electrode, platinum wire as counter electrode was used. Prior to every measurement, the electrolyte was purged with >99% pure Ar through a porous glass frit to remove oxygen and ensure O_2 free environment. Electrochemical measurements were recorded and analyzed using a multichannel potentiostat (Autolab M204, Metrohm) and the NOVA software.

Cyclic voltammetry measurements were performed in a potential window of 0 ~ −1.6 V vs. $Ag/AgNO_3$ in organic solvents and 0 ~ −1 V vs. Ag/AgCl for aqueous solvent.

The redox conductivity of the COF film was carried out using electrochemical impedance spectroscopy (EIS) under the same experimental electrochemistry conditions as stated above. Chronoamperometry was performed for preconditioning the thin films at desirable applied potentials (−0.4 V ~ −1.6 V vs. $Ag/AgNO_3$) for two minutes. EIS measurements were performed with a 10 mV AC potential modulation in a frequency range of 0.1–10,000 Hz.

For photocharging experiments, 365 nm UV LED was used as the irradiation source. The intensity of the illumination was measured by a calibrated THORLABS S310C thermal power meter and further confirmed by a calibrated OCEAN OPTICS USB4000 spectrometer. The intensity was set to $P_{nominal} \approx$ 100 mW cm^{-2} for all the irradiation experiments.

2. Synthesis and Characterizations

Synthesis of TAPT-NDI COF: TAPT-NDI COF was synthesized following a literature with a slight modification. In a 8 mL Schlenk bomb, 1, 4, 5, 8-naphthalenetetracarboxylic dianhydride (NTCDA, 40.2 mg, 0.15 mmol) and 1, 3, 5-tris-(4-aminophenyl)triazine (TAPT, 35.1 mg, 0.1 mmol) were suspended in a mixture of N-methyl-2-pyrrolidone (0.75 mL), and mesitylene (0.15 mL). The mixture was sonicated for 5 minutes in order to get a homogenous dispersion and isoquinoline (0.05 mL) was added followed by sonication for 2 minutes. The mixture was frozen in a liquid N_2 bath, the inner atmosphere was evacuated at < 0.01 mbar and three cycles of freeze–pump–thaw was performed. The mixture was sonicated for 5 minutes, and heated in aluminium heating block for 4 days at 120 °C. After cooling to room temperature, the precipitate was collected by filtration and washed with hot dimethylformamide, ethanol and tetrahydrofuran, while keeping the solids wet. An overnight Soxhlet treatment in tetrahydrofuran was performed, followed by $scCO_2$ drying, which resulted TAPT-NDI COF as a light-yellow solid with a yield of 61.4 mg.

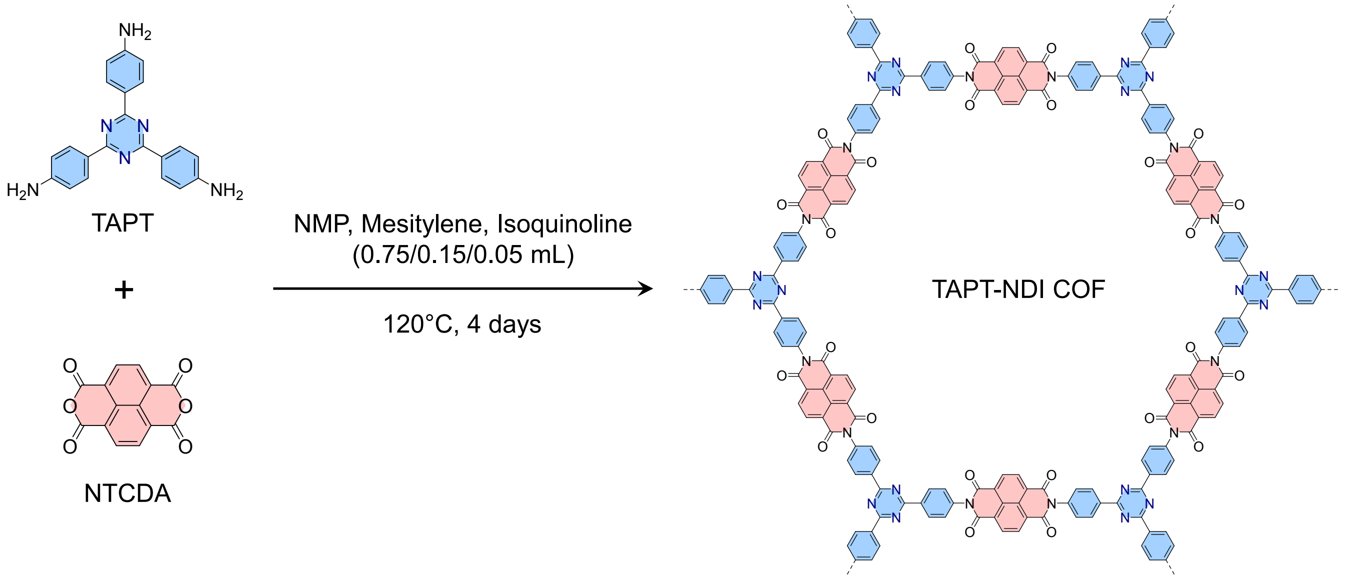

Figure S1. Synthetic procedure and chemical structure of TAPT-NDI COF.

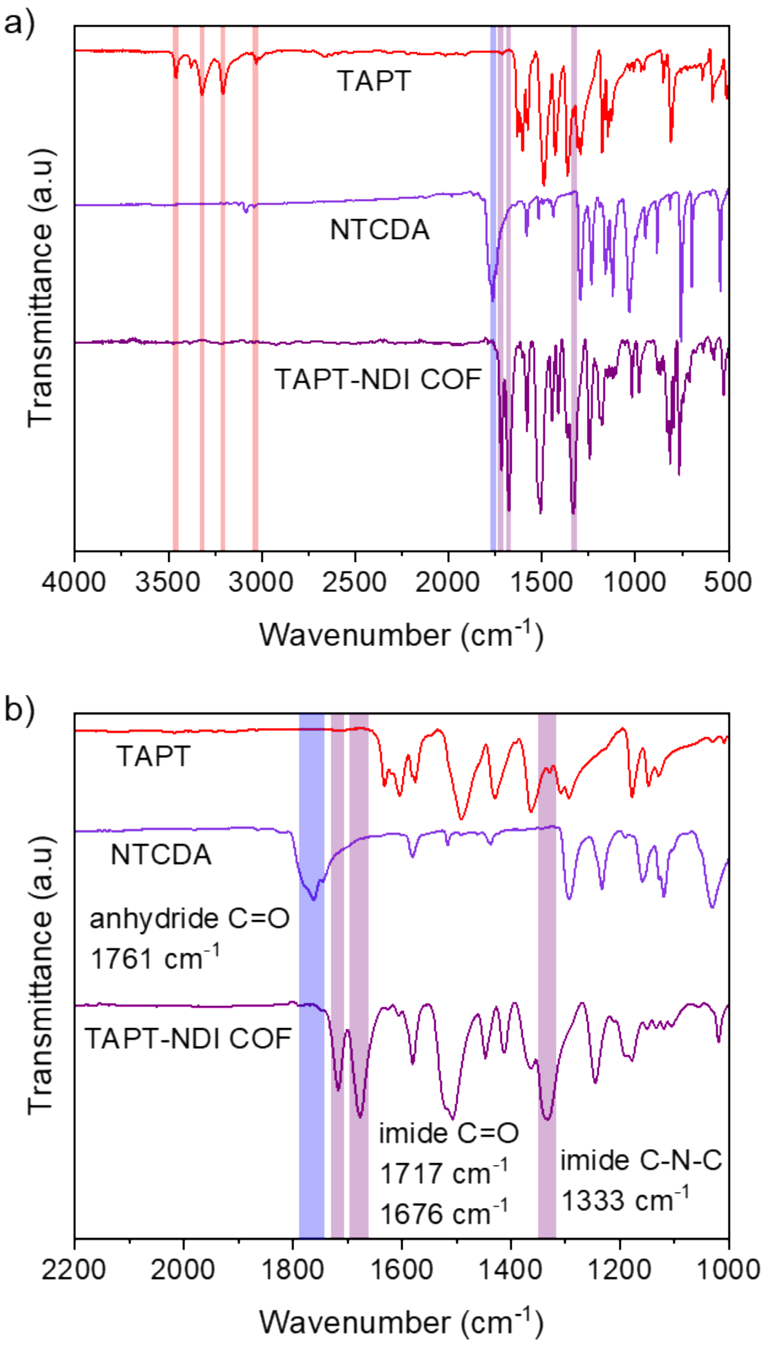

Figure S2. (a) FTIR spectra of TAPT (red), NTCDA (blue), and TAPT-NDI COF (purple) highlighting the N-H bands from TAPT (light red), the C=O band from NTCDA (light blue), and the five-membered imide ring bands from TAPT-NDI COF (light purple). (b) Enlarged section of the FTIR spectra highlighting the C=O band from NTCDA (light blue) and the five-membered imide ring bands from TAPT-NDI COF (light purple).

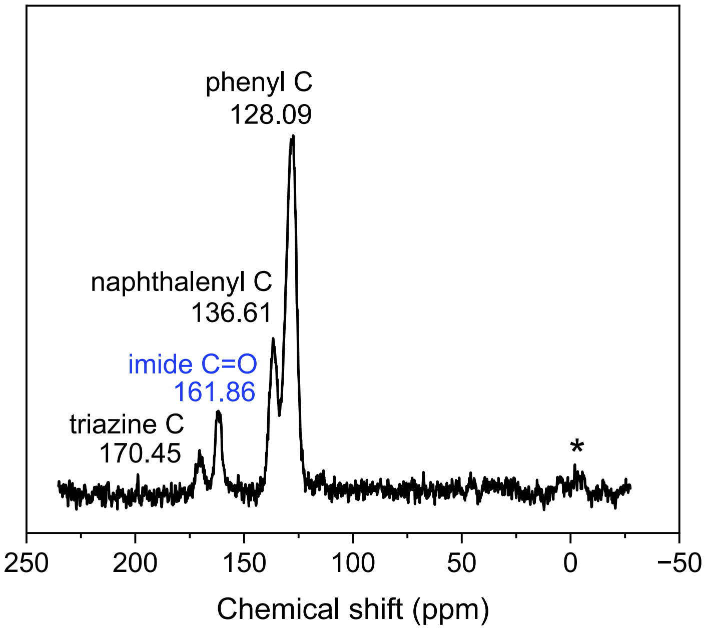

Figure S3. ^{13}C–CP/MAS-NMR spectrum of TAPT-NDI COF showing the characteristic imide feature.

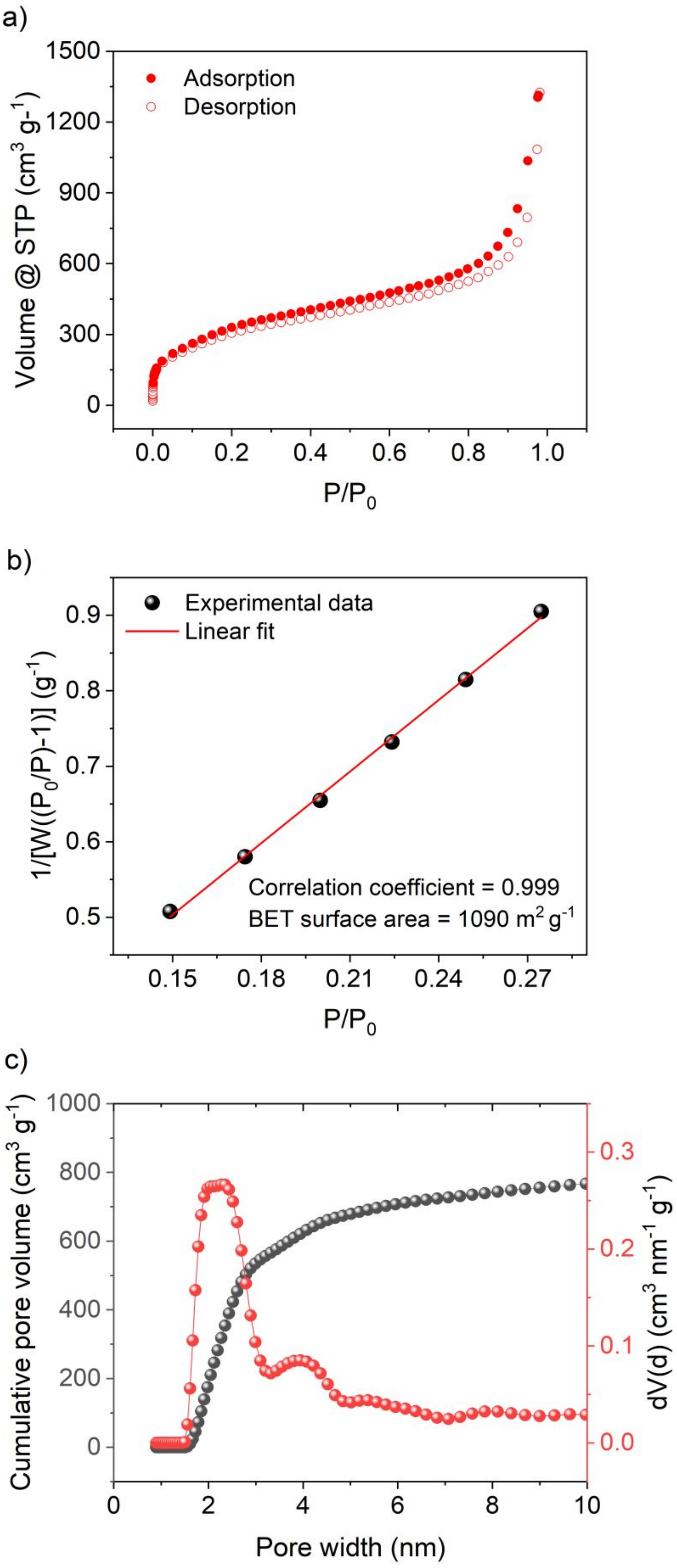

Figure S4. (a) N_2 sorption isotherm, (b) BET plot showing the relative pressure (P/P_0) vs. $1/[W((P_0/P)-1)]$ BET function, and (c) cumulative pore volume and pore size distribution of TAPT-NDI COF.

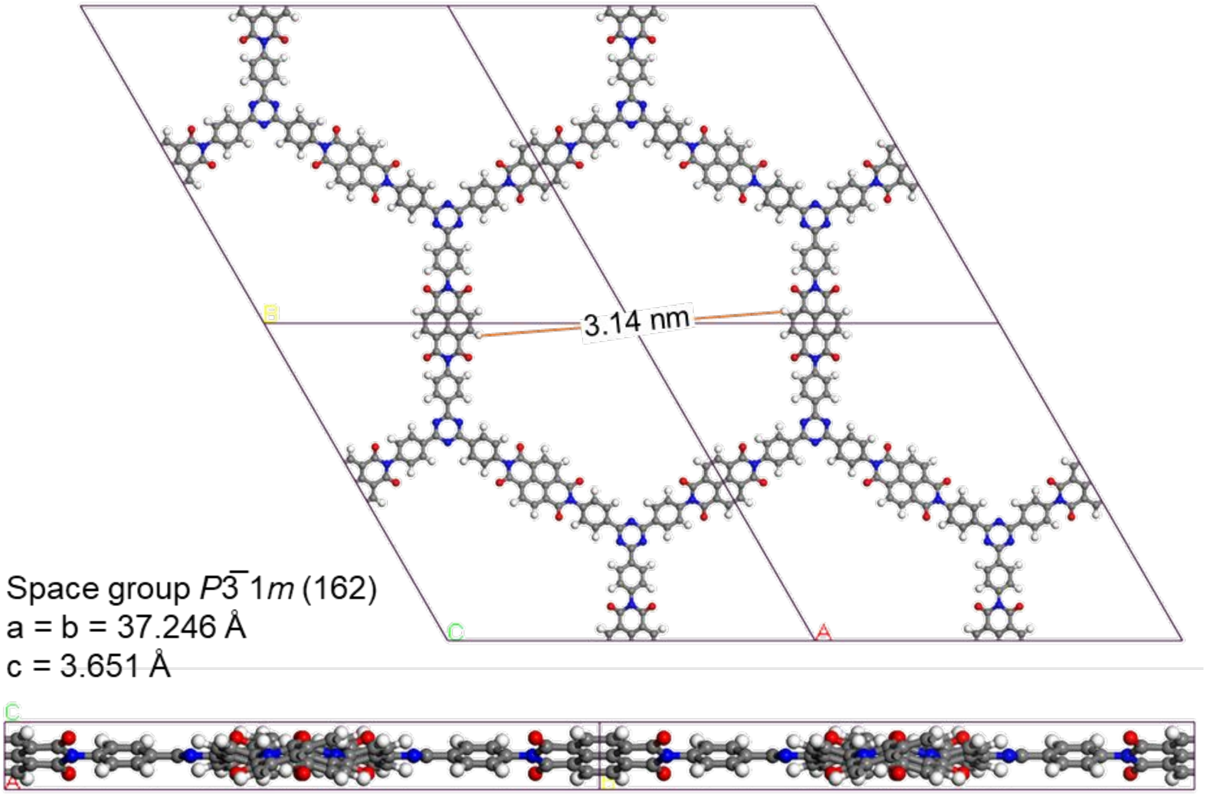

Figure S5. Simulated TAPT-NDI COF structure showing different orientations.

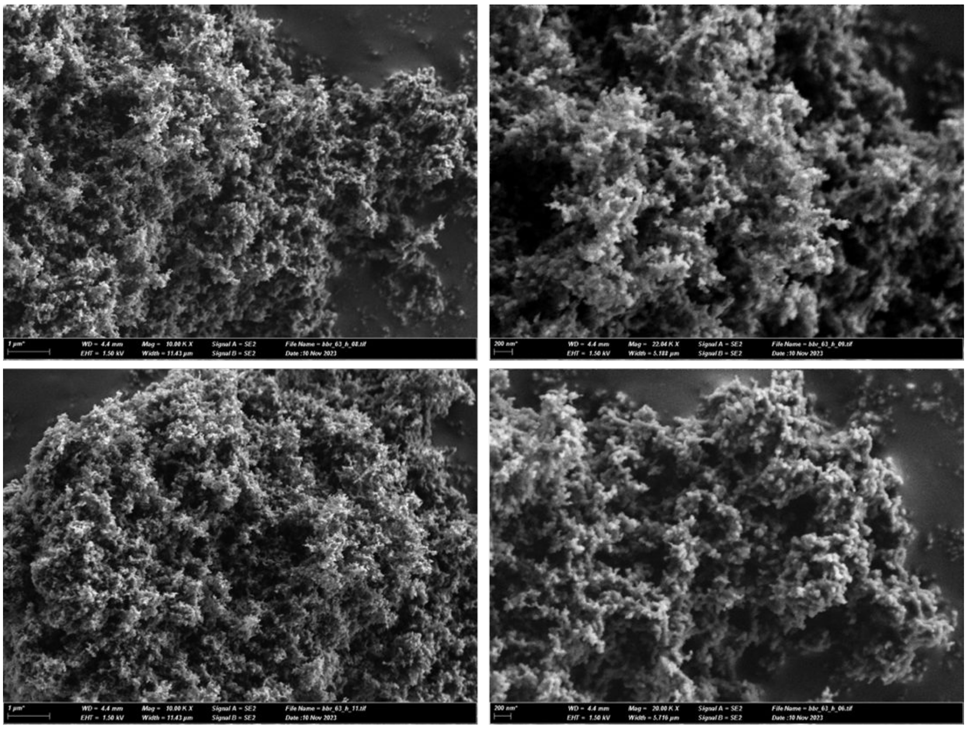

Figure S6. InLens SEM images of TAPT-NDI COF at various magnifications.

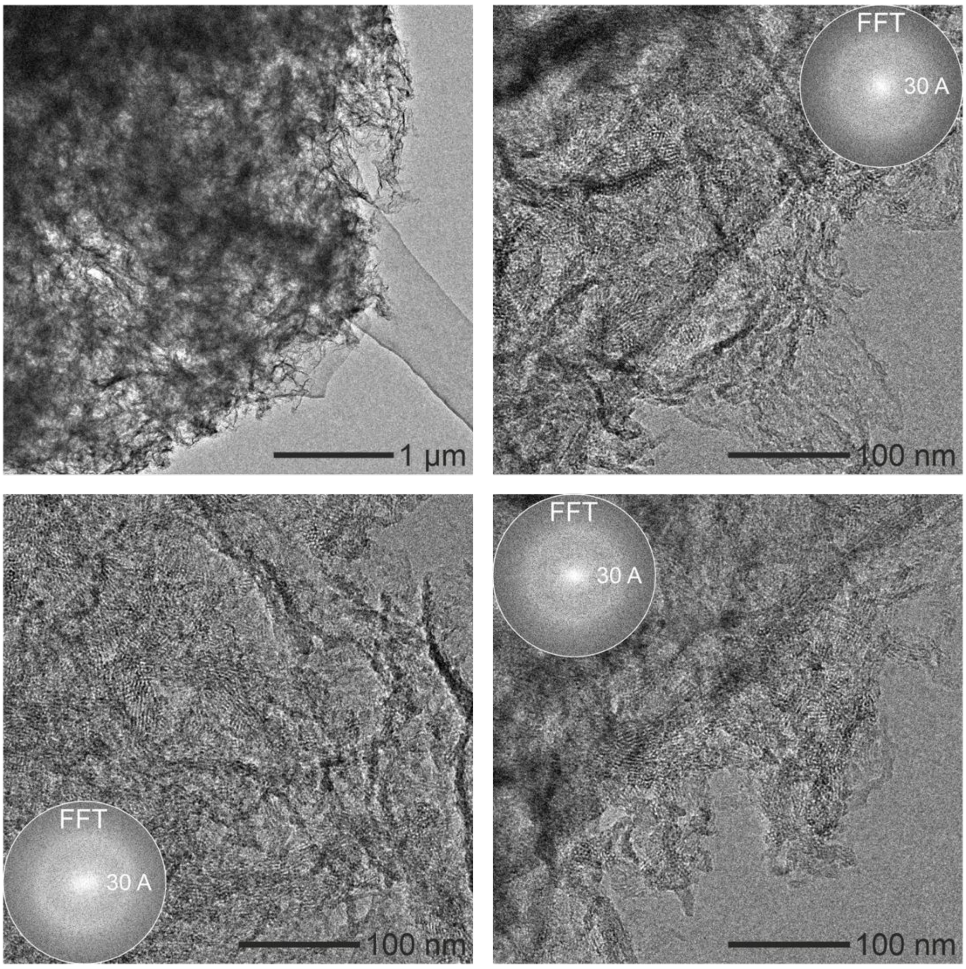

Figure S7. TEM images of TAPT-NDI COF at various magnifications.

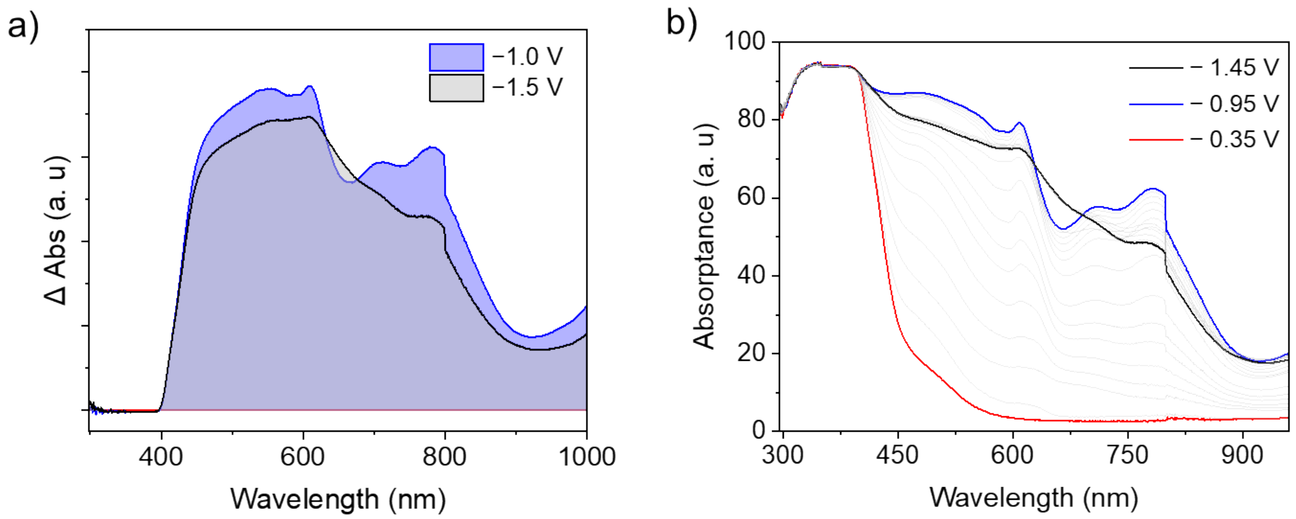

Figure S8. (a) Change in UV-vis absorptance spectra of TAPT-NDI COF during cathodic scans at –1.0 and –1.5 V. (b) UV-vis spectroelectrochemical analysis of TAPT-NDI COF, showing the reversible transformation during an anodic scan (–1.45 V for NDI^{2-}, –0.95 V for $NDI^{\bullet-}$, –0.35 V for NDI^{0}).

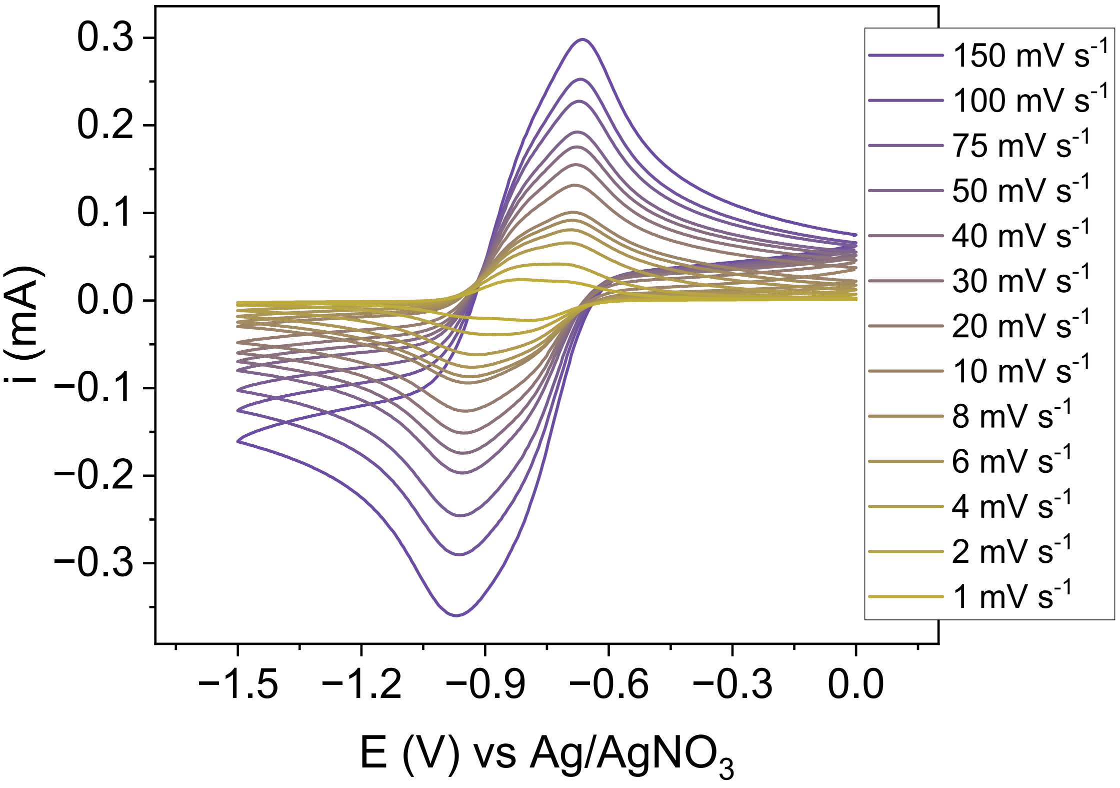

Figure S9. Cyclic voltammograms of TAPT-NDI COF@FTO in Ar-saturated MeCN with 0.1 M $LiClO_4$ as supporting electrolyte at various scan rates from 1 to 150 mV s^{-1}.

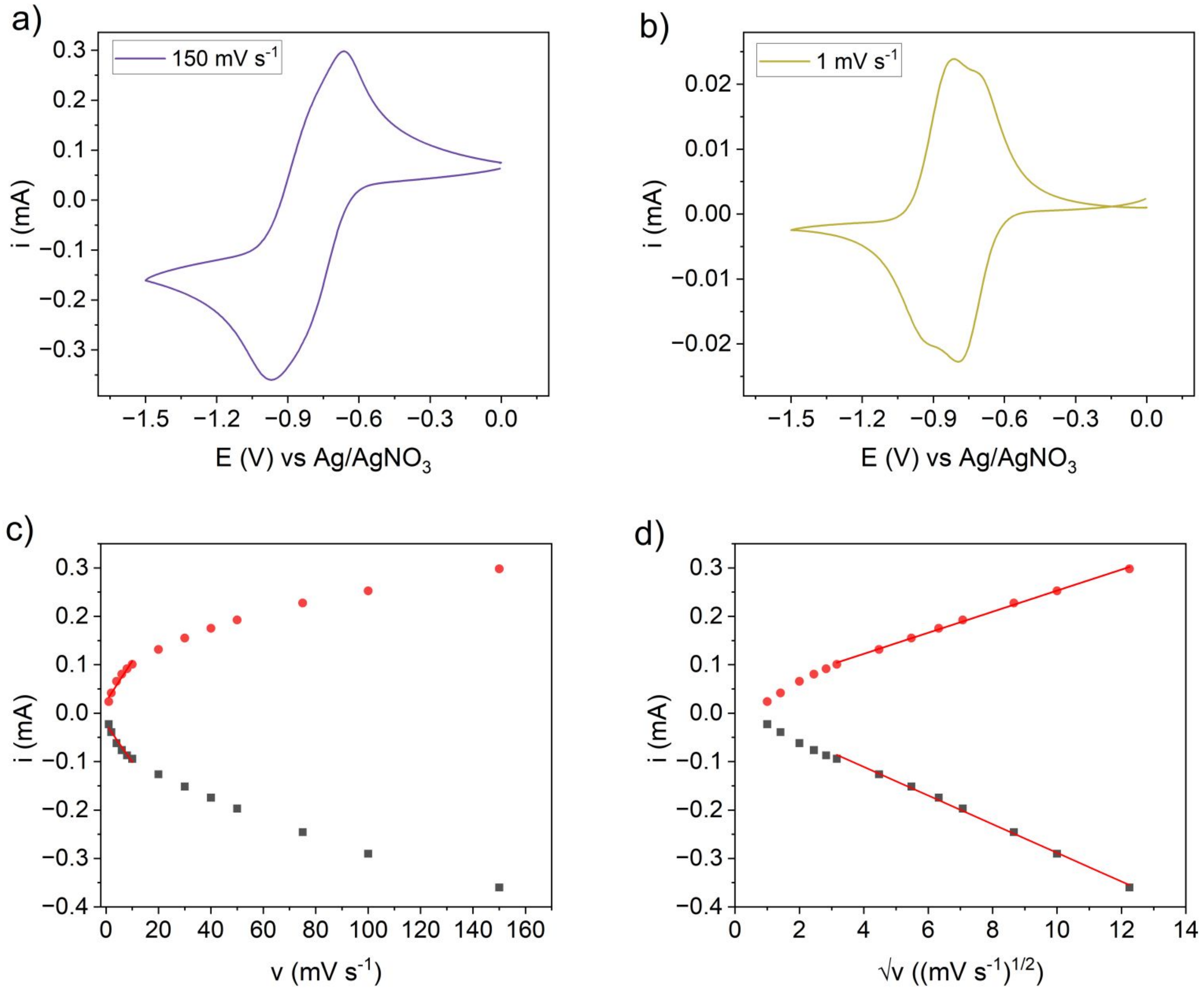

Figure S10. Cyclic voltammograms of TAPT-NDI COF@FTO in Ar-saturated MeCN with 0.1 M $LiClO_4$ as supporting electrolyte at a scan rate of 150 mV s^{-1} (a) and 1 mV s^{-1} (b). The plot of cathodic and anodic peak currents corresponding to the $NDI^{0/•−}$ redox couple, *i* vs. *v* (c) and *i* vs. $v^{-1/2}$ (d), discloses the transition scan rate (10 mV s^{-1}) between the two limiting regimes.

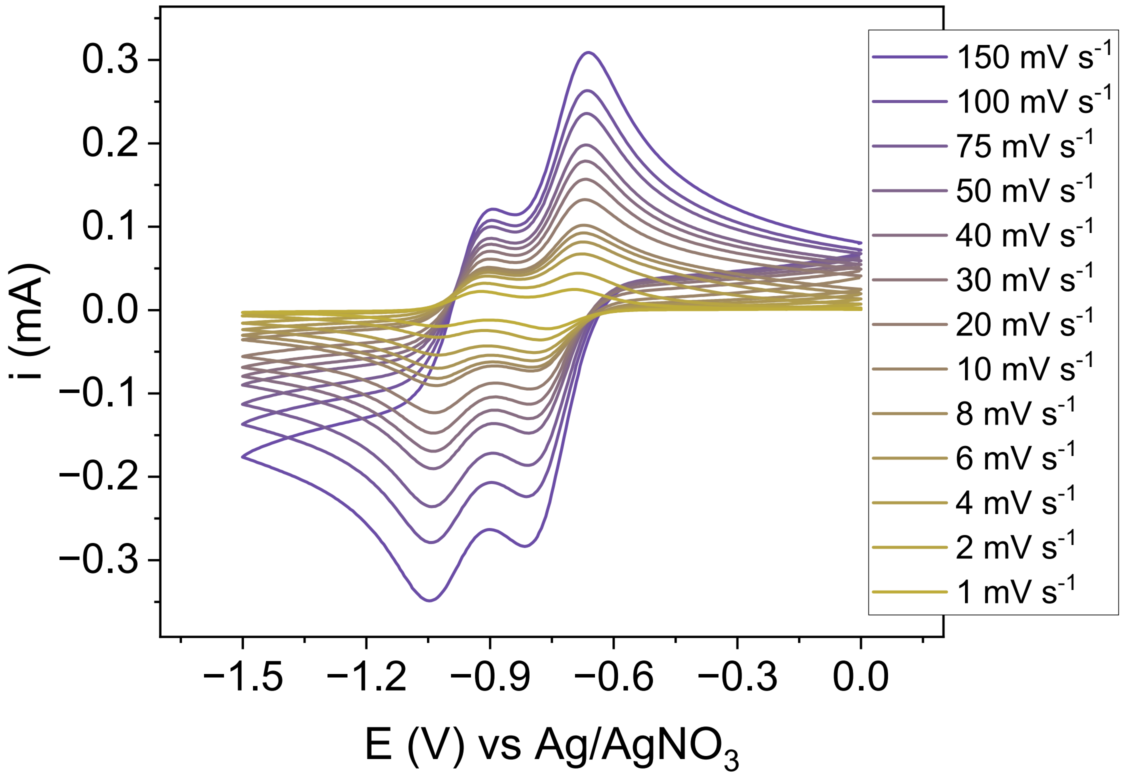

Figure S11. Cyclic voltammograms of TAPT-NDI COF@FTO in Ar-saturated MeCN with 0.1 M $NaClO_4$ as supporting electrolyte at various scan rates from 1 to 150 mV s^{-1}.

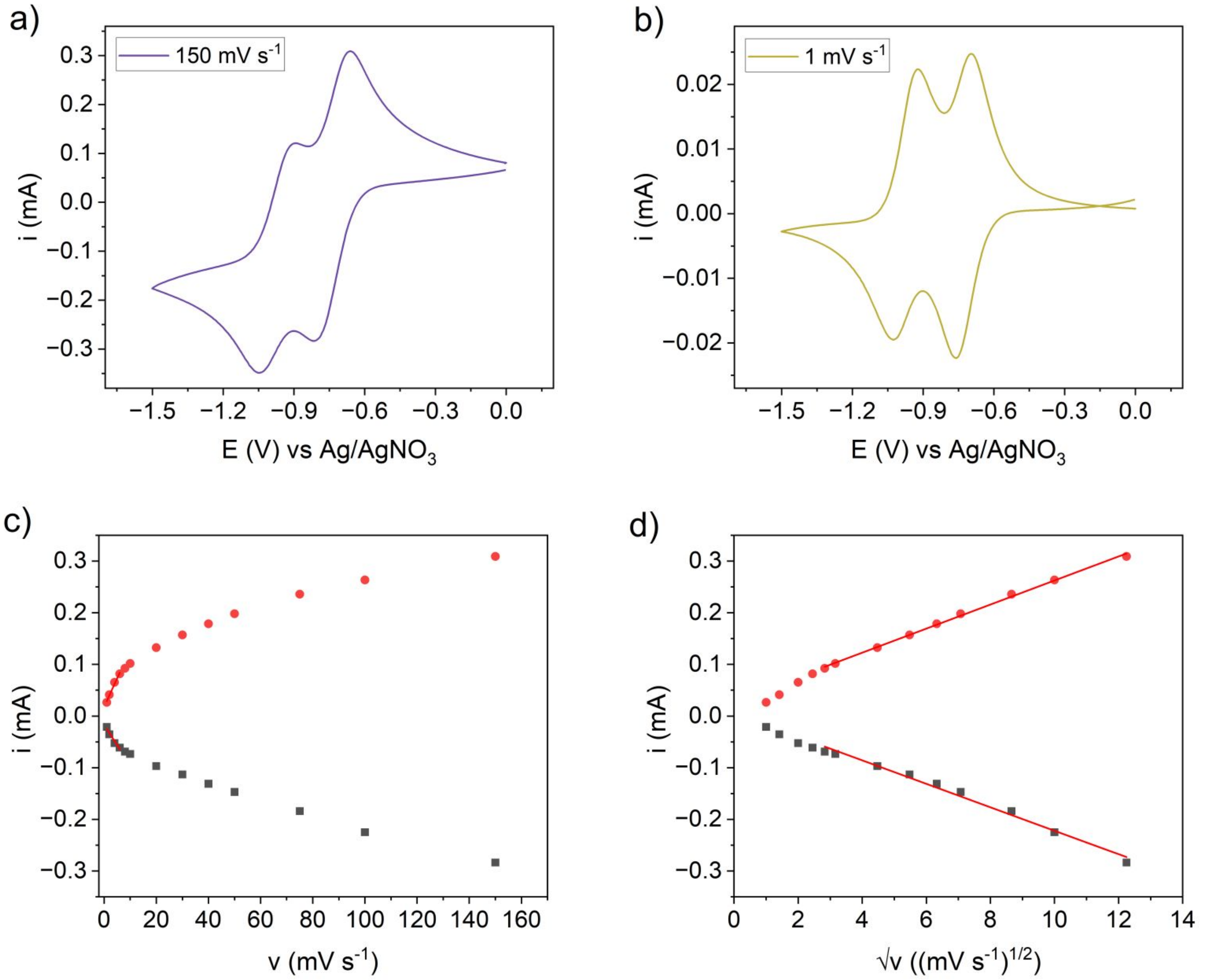

Figure S12. Cyclic voltammograms of TAPT-NDI COF@FTO in Ar-saturated MeCN with 0.1 M $NaClO_4$ as supporting electrolyte at a scan rate of 150 mV s^{-1} (a) and 1 mV s^{-1} (b). The plot of cathodic and anodic peak currents corresponding to the $NDI^{0/\bullet-}$ redox couple, *i* vs. *v* (c) and *i* vs. $v^{-1/2}$ (d), discloses the transition scan rate (10 mV s^{-1}) between the two limiting regimes.

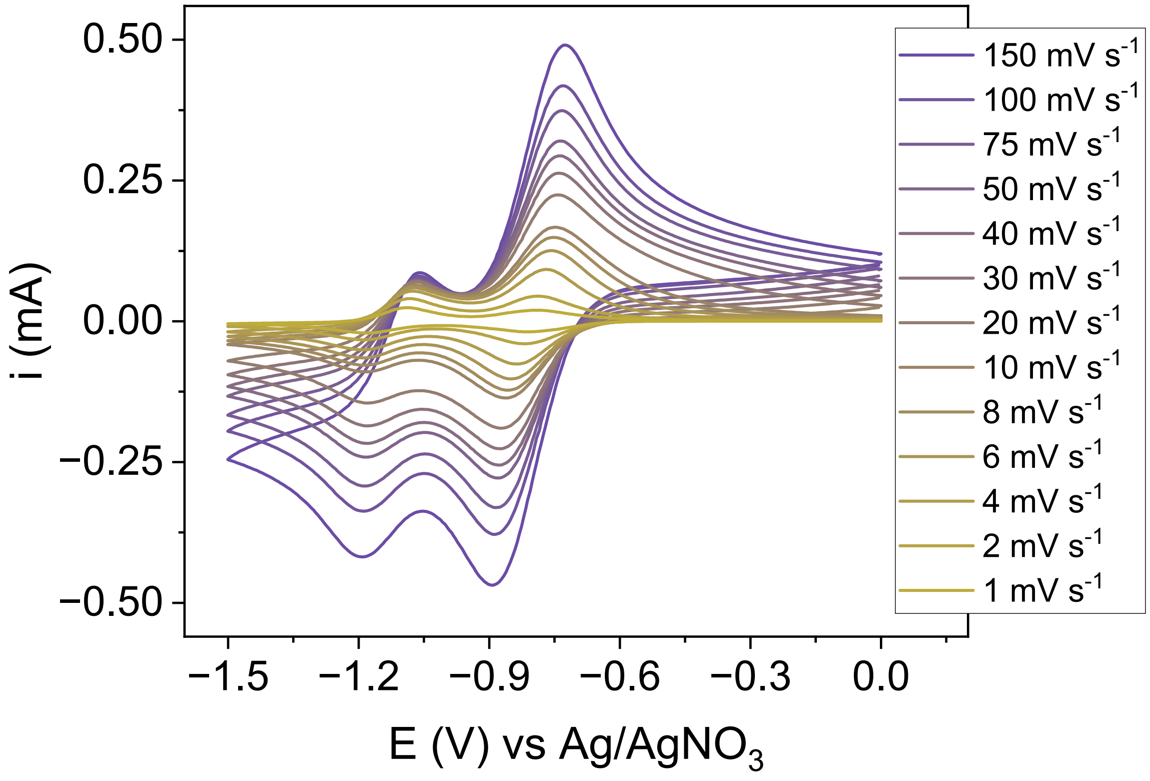

Figure S13. Cyclic voltammograms of TAPT-NDI COF@FTO in Ar-saturated MeCN with 0.1 M KPF_6 as supporting electrolyte at various scan rates from 1 to 150 mV s^{-1}.

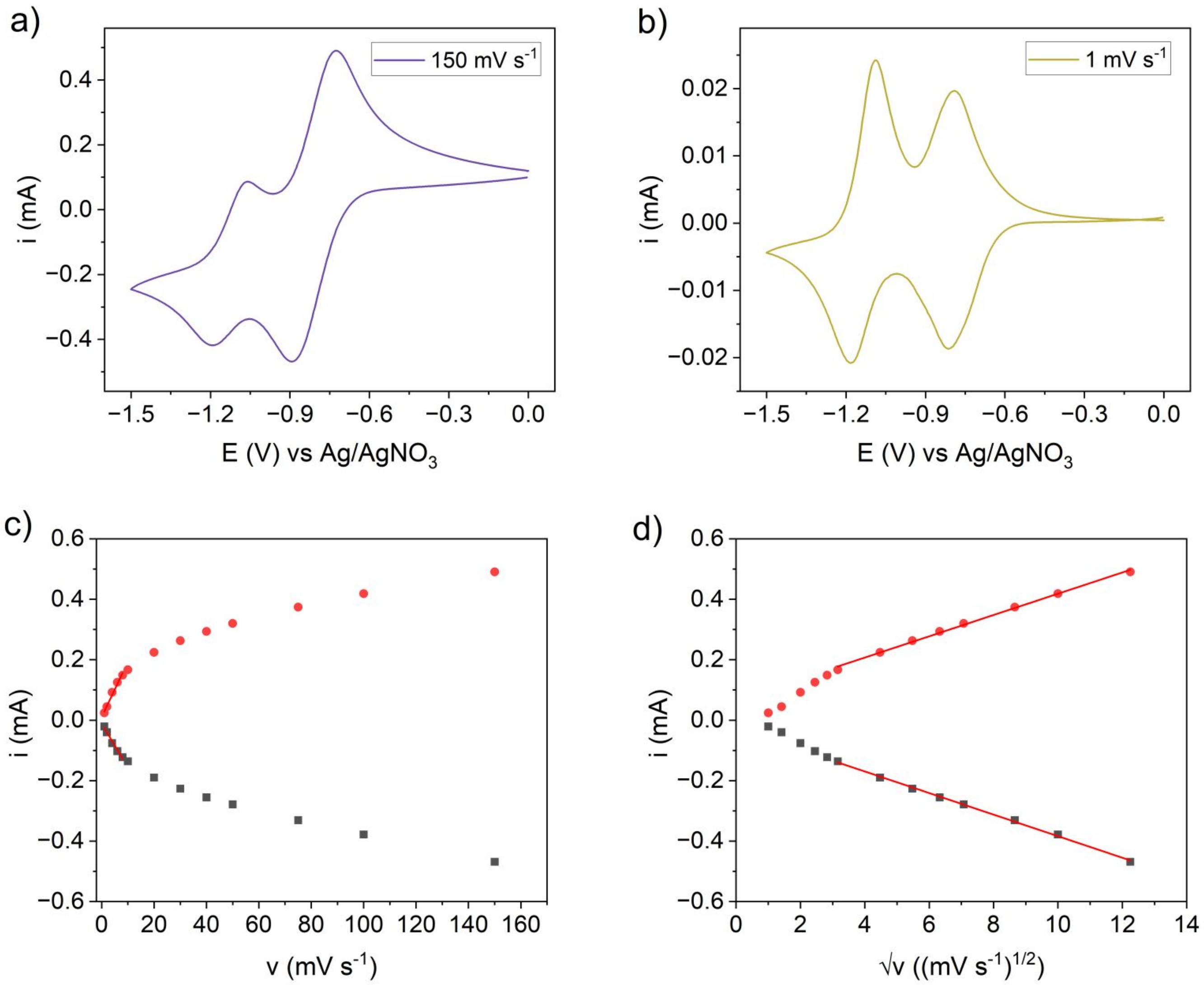

Figure S14. Cyclic voltammograms of TAPT-NDI COF@FTO in Ar-saturated MeCN with 0.1 M KPF_6 as supporting electrolyte at a scan rate of 150 mV s^{-1} (a) and 1 mV s^{-1} (b). The plot of cathodic and anodic peak currents corresponding to the $NDI^{0/\bullet-}$ redox couple, *i* vs. *v* (c) and *i* vs. $v^{-1/2}$ (d), discloses the transition scan rate (10 mV s^{-1}) between the two limiting regimes.

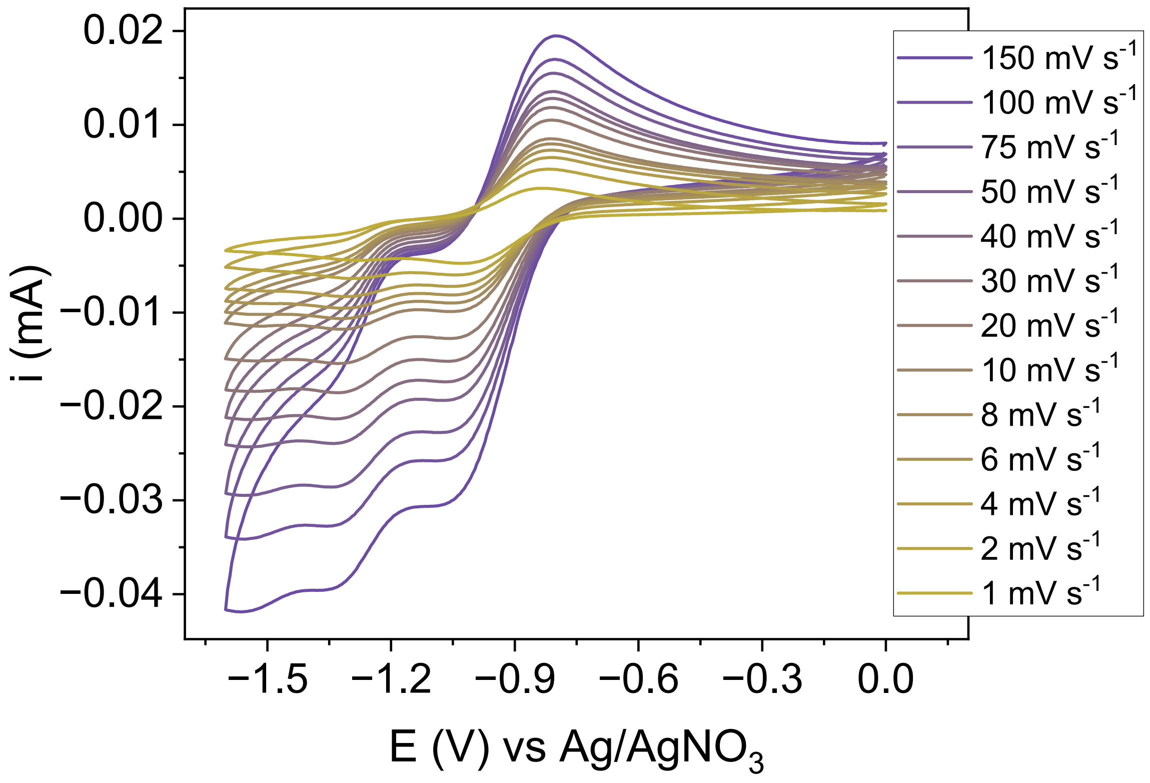

Figure S15. Cyclic voltammograms of TAPT-NDI COF@FTO in Ar-saturated DMF with 0.1 M $LiClO_4$ as supporting electrolyte at various scan rates from 1 to 150 mV s^{-1}.

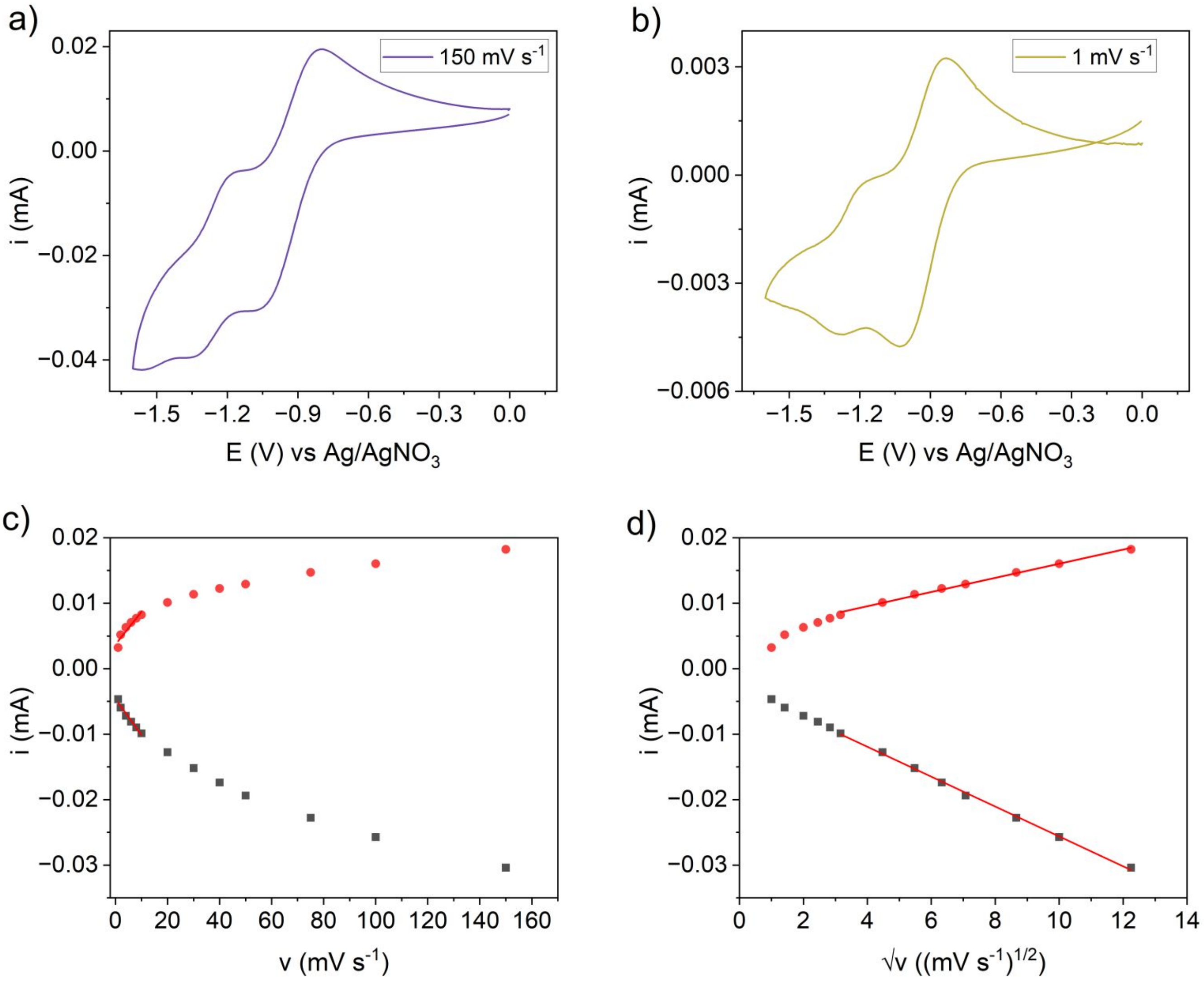

Figure S16. Cyclic voltammograms of TAPT-NDI COF@FTO in Ar-saturated DMF with 0.1 M $LiClO_4$ as supporting electrolyte at a scan rate of 150 mV s^{-1} (a) and 1 mV s^{-1} (b). The plot of cathodic and anodic peak currents corresponding to the $NDI^{0/\bullet-}$ redox couple, *i* vs. *v* (c) and *i* vs. $v^{-1/2}$ (d), discloses the transition scan rate (8 mV s^{-1}) between the two limiting regimes.

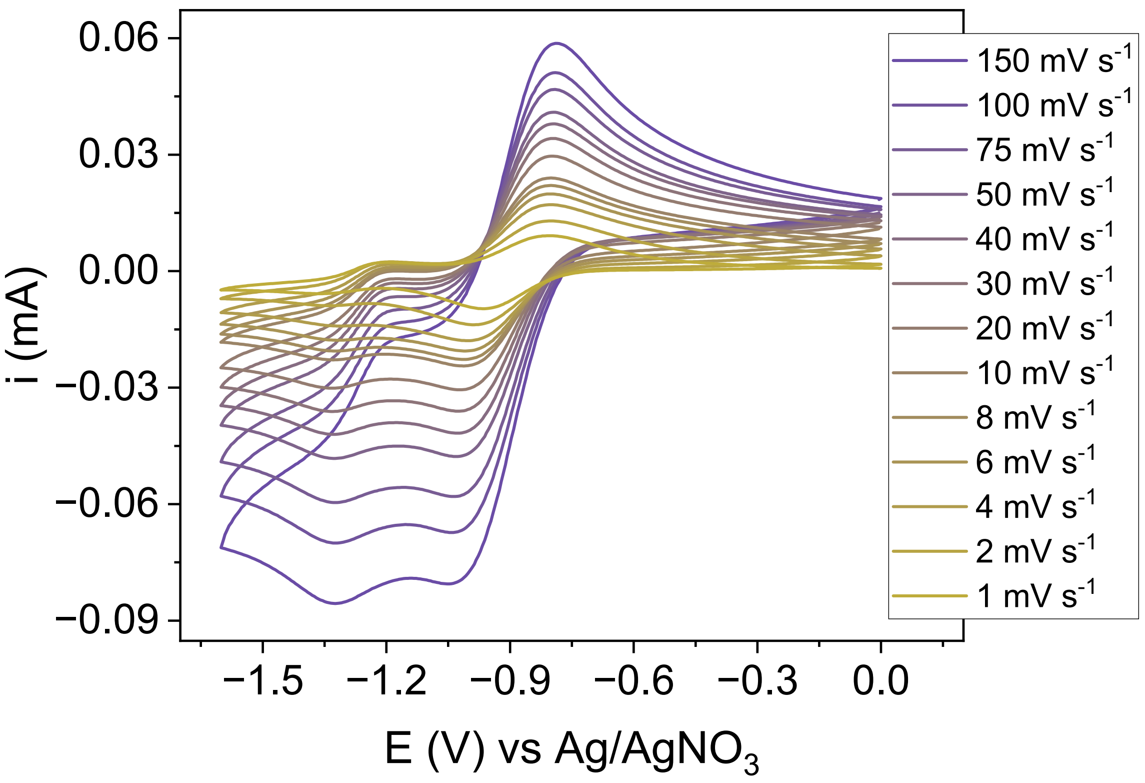

Figure S17. Cyclic voltammograms of TAPT-NDI COF@FTO in Ar-saturated DMF with 0.1 M $NaClO_4$ as supporting electrolyte at various scan rates from 1 to 150 mV s^{-1}.

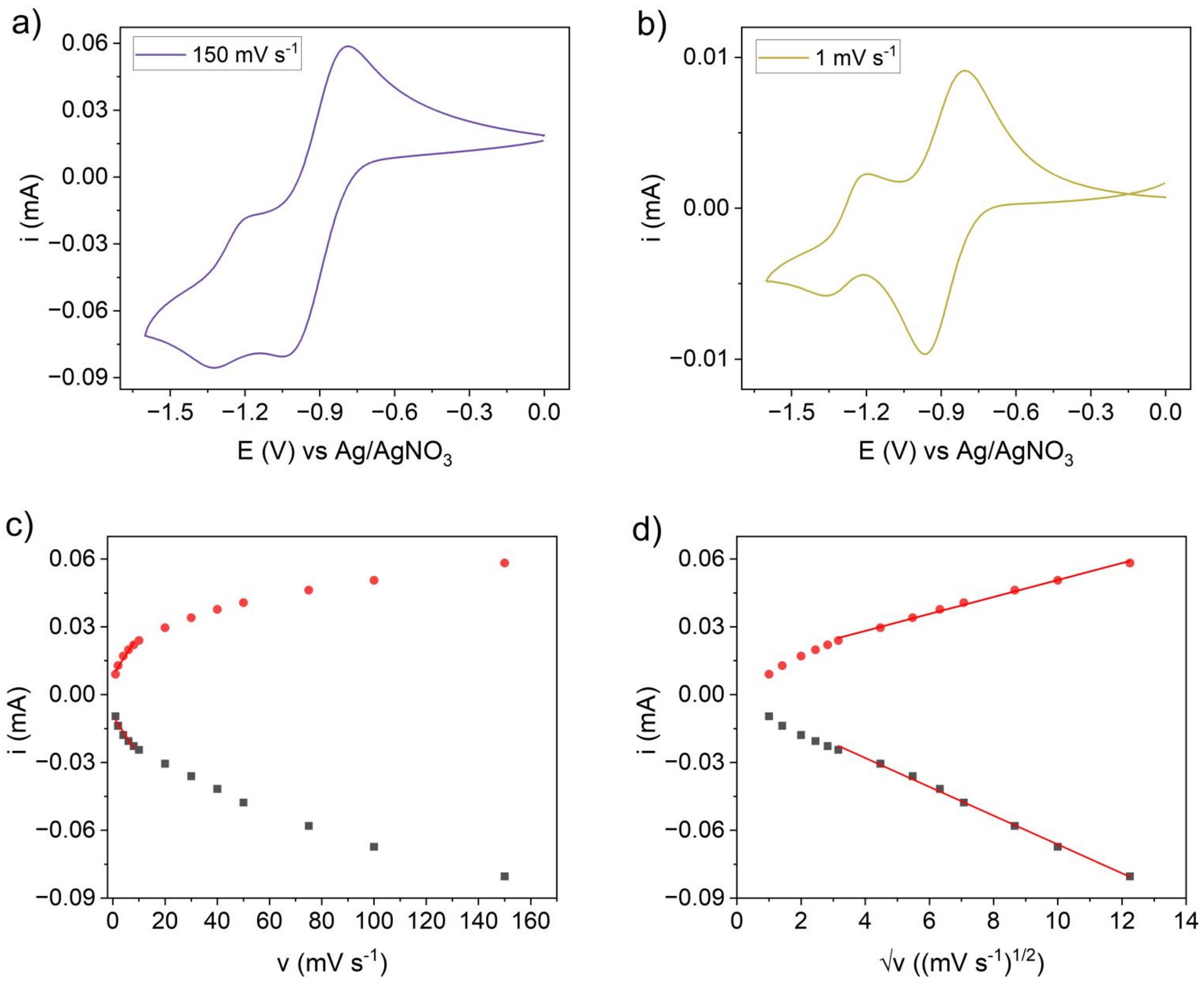

Figure S18. Cyclic voltammograms of TAPT-NDI COF@FTO in Ar-saturated DMF with 0.1 M $NaClO_4$ as supporting electrolyte at a scan rate of 150 mV s^{-1} (a) and 1 mV s^{-1} (b). The plot of cathodic and anodic peak currents corresponding to the $NDI^{0/\bullet-}$ redox couple, *i* vs. *v* (c) and *i* vs. $v^{-1/2}$ (d), discloses the transition scan rate (8 mV s^{-1}) between the two limiting regimes.

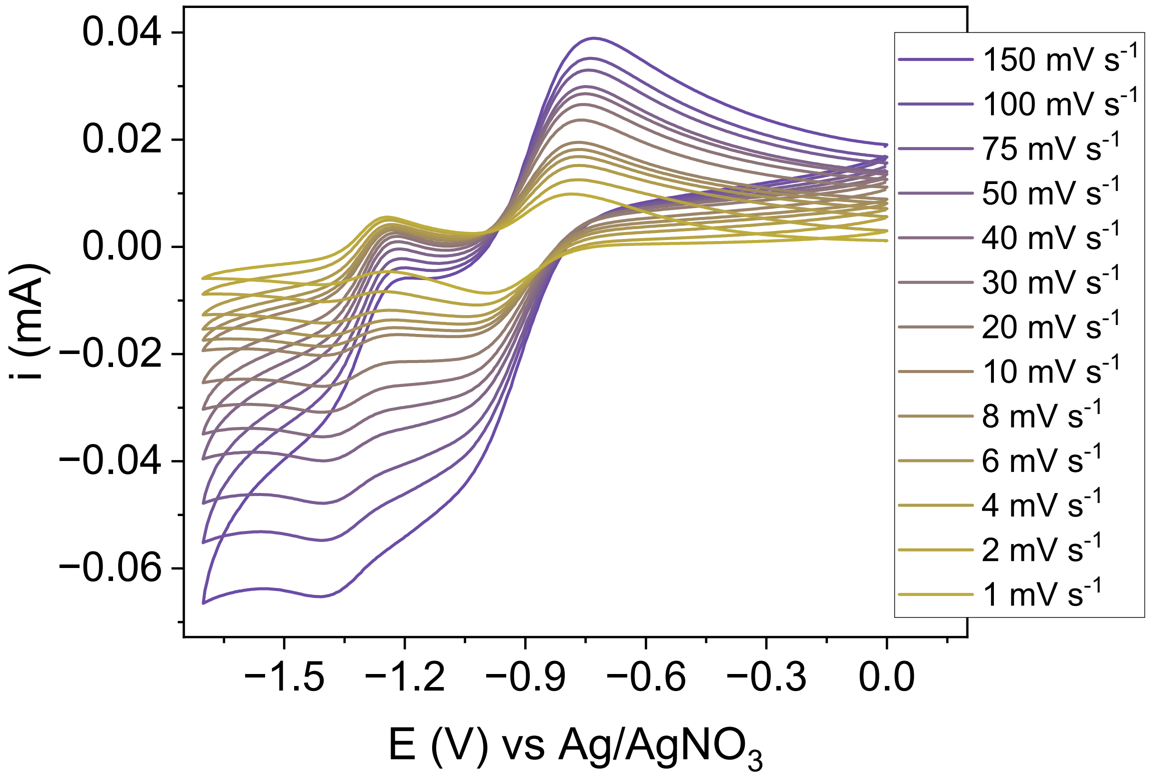

Figure S19. Cyclic voltammograms of TAPT-NDI COF@FTO in Ar-saturated DMF with 0.1 M $KClO_4$ as supporting electrolyte at various scan rates from 1 to 150 mV s^{-1}.

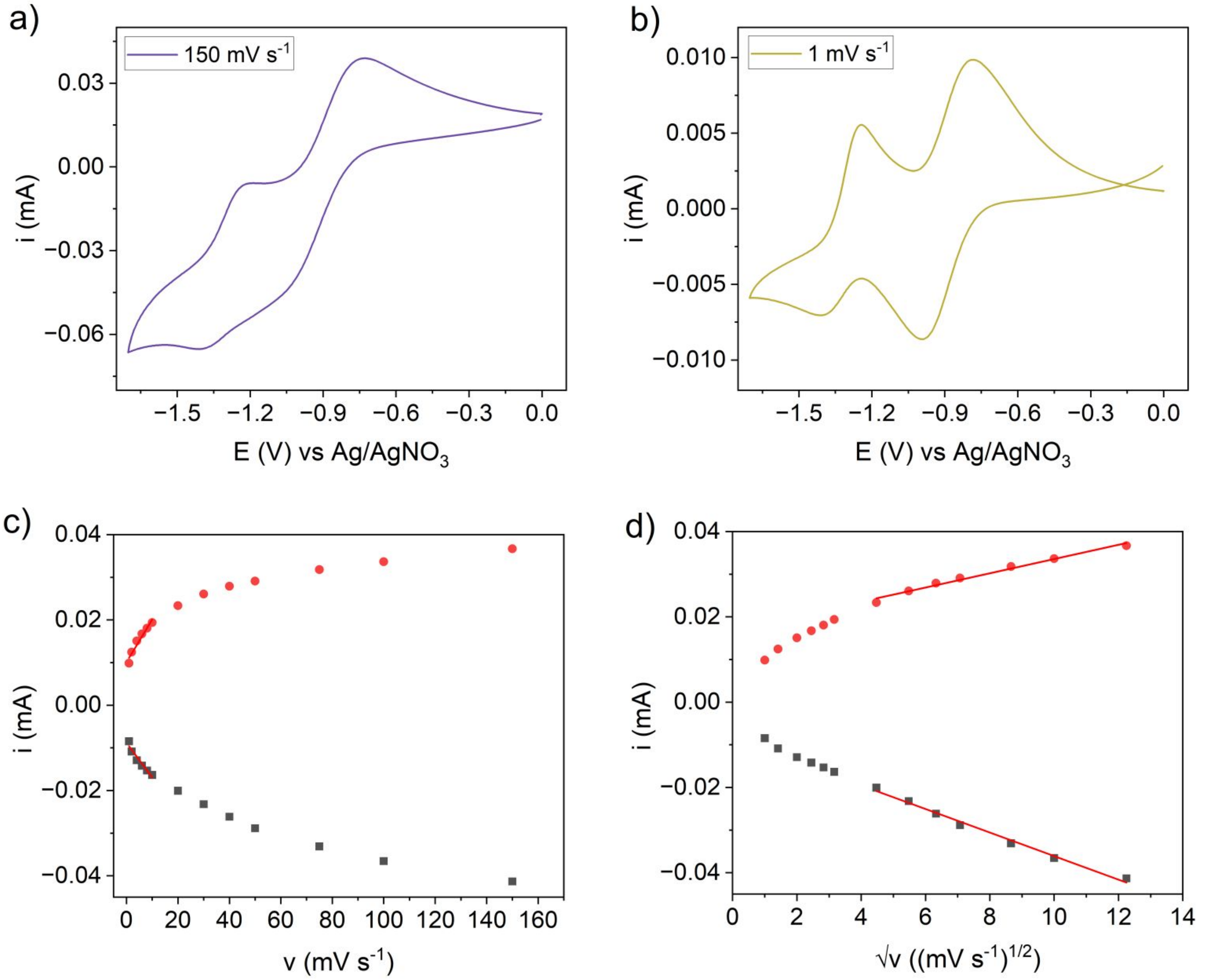

Figure S20. Cyclic voltammograms of TAPT-NDI COF@FTO in Ar-saturated DMF with 0.1 M $KClO_4$ as supporting electrolyte at a scan rate of 150 mV s^{-1} (a) and 1 mV s^{-1} (b). The plot of cathodic and anodic peak currents corresponding to the $NDI^{0/\bullet-}$ redox couple, *i* vs. *v* (c) and *i* vs. $v^{-1/2}$ (d), discloses the transition scan rate (10 mV s^{-1}) between the two limiting regimes.

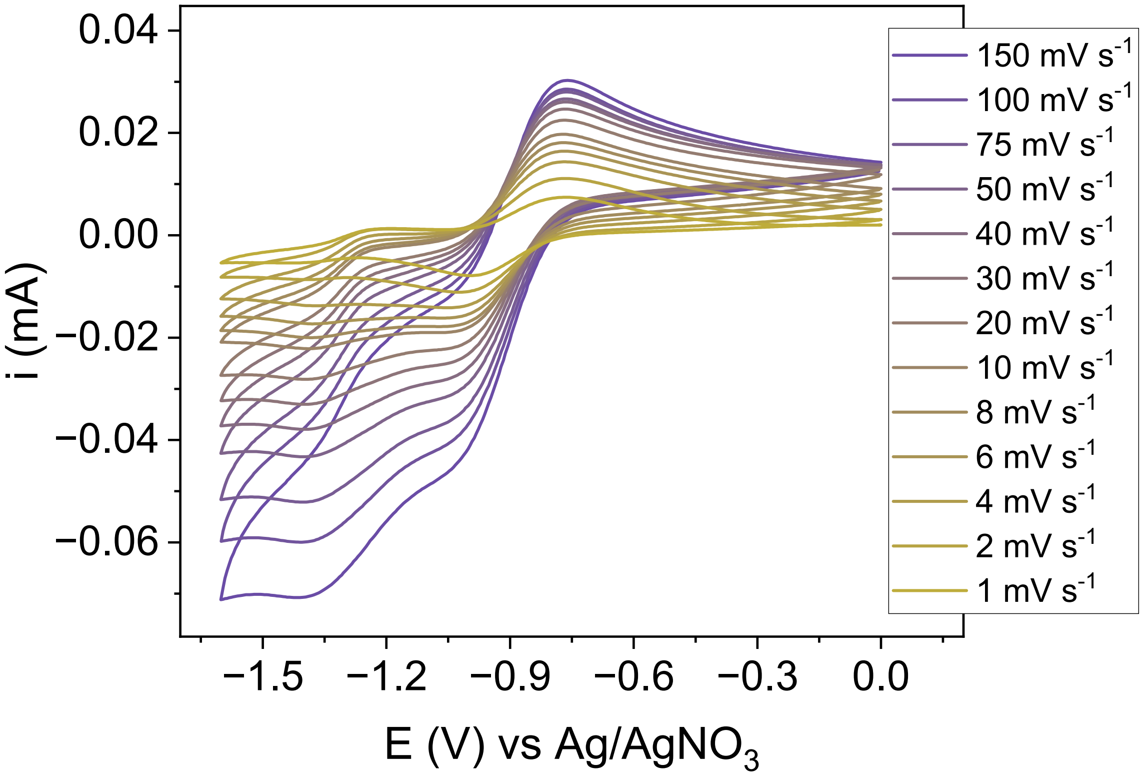

Figure S21. Cyclic voltammograms of TAPT-NDI COF@FTO in Ar-saturated DMF with 0.1 M KPF_6 as supporting electrolyte at various scan rates from 1 to 150 mV s^{-1}.

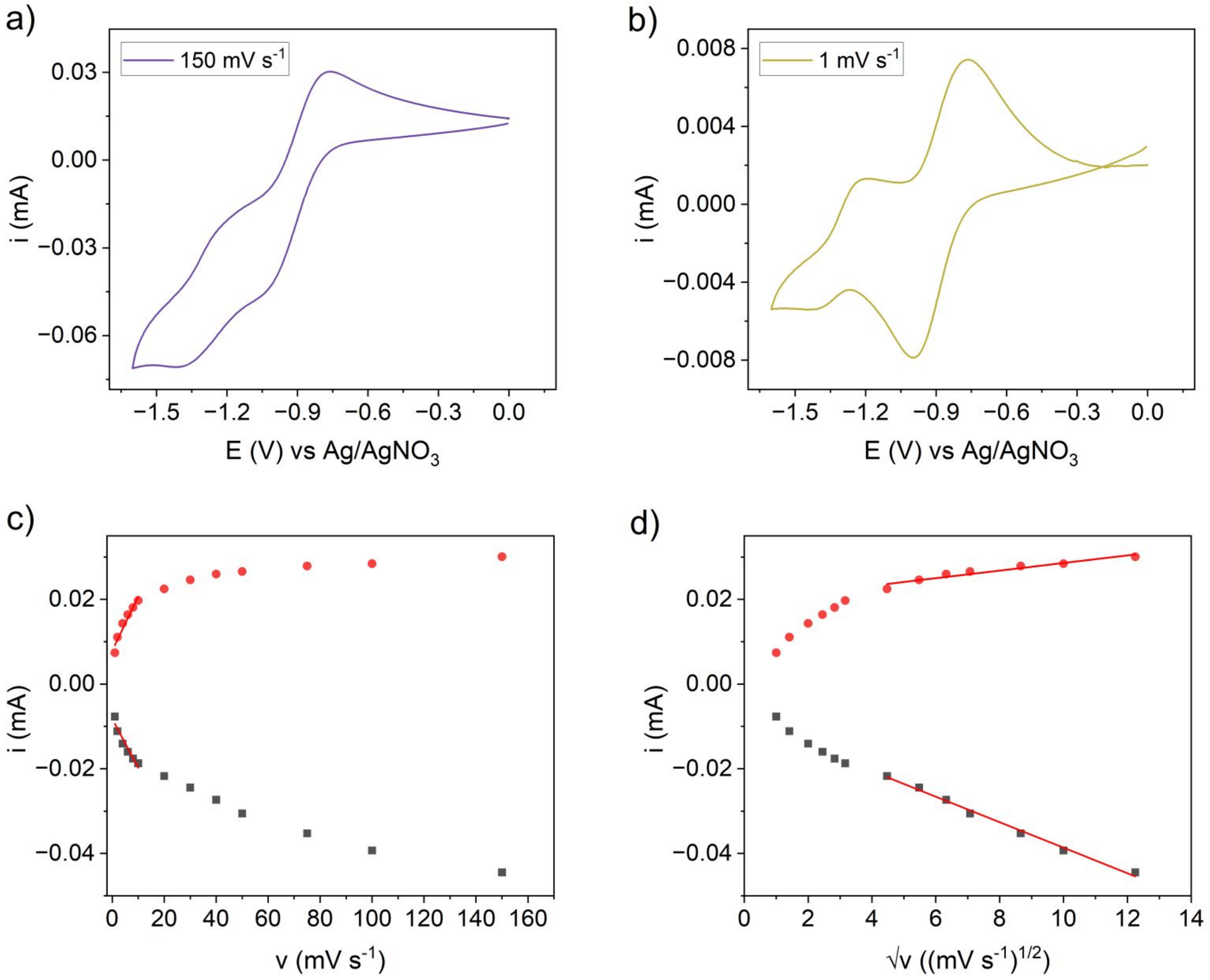

Figure S22. Cyclic voltammograms of TAPT-NDI COF@FTO in Ar-saturated DMF with 0.1 M KPF_6 as supporting electrolyte at a scan rate of 150 mV s^{-1} (a) and 1 mV s^{-1} (b). The plot of cathodic and anodic peak currents corresponding to the $NDI^{0/\bullet-}$ redox couple, *i* vs. *v* (c) and *i* vs. $v^{-1/2}$ (d), discloses the transition scan rate (8 mV s^{-1}) between the two limiting regimes.

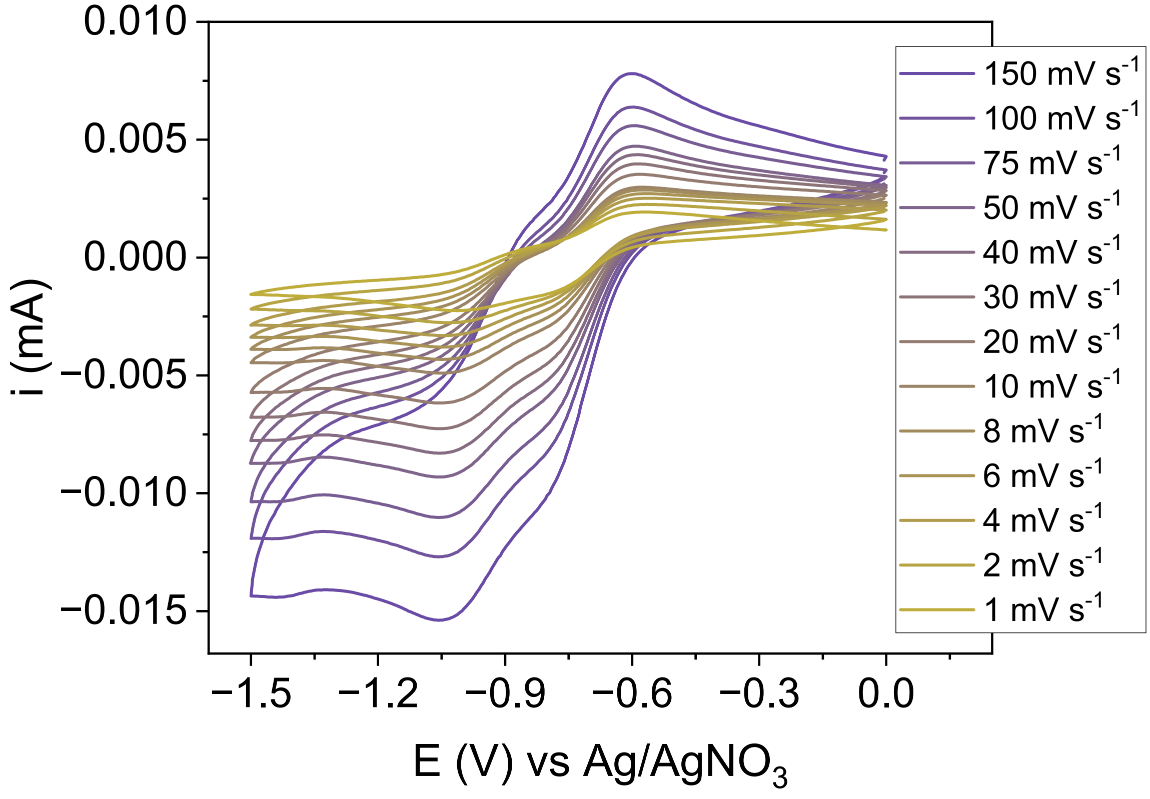

Figure S23. Cyclic voltammograms of TAPT-NDI COF@FTO in Ar-saturated EtOH with 0.1 M $LiClO_4$ as supporting electrolyte at various scan rates from 1 to 150 mV s^{-1}.

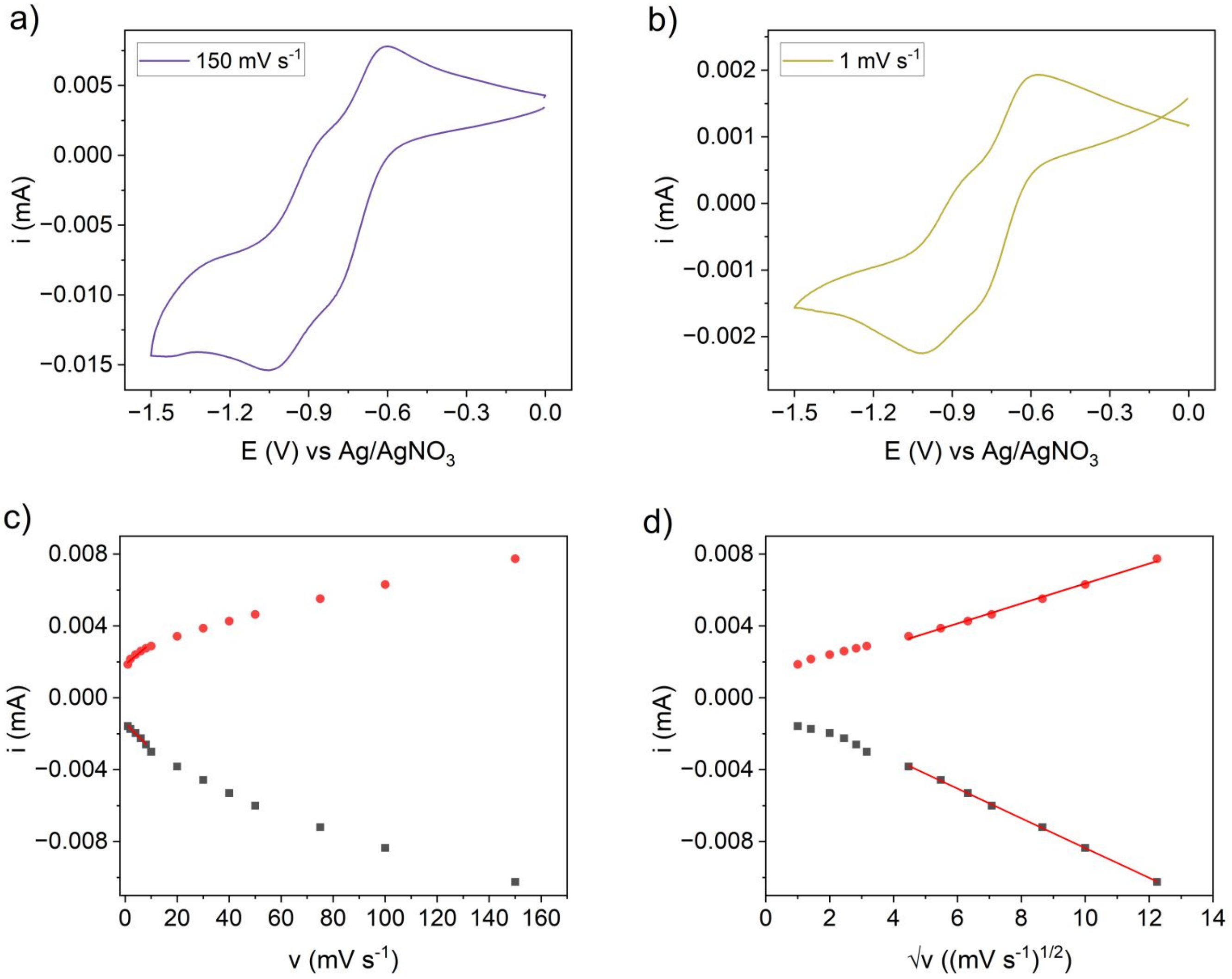

Figure S24. Cyclic voltammograms of TAPT-NDI COF@FTO in Ar-saturated EtOH with 0.1 M $LiClO_4$ as supporting electrolyte at a scan rate of 150 mV s^{-1} (a) and 1 mV s^{-1} (b). The plot of cathodic and anodic peak currents corresponding to the $NDI^{0/\bullet-}$ redox couple, *i* vs. *v* (c) and *i* vs. $v^{-1/2}$ (d), discloses the transition scan rate (10 mV s^{-1}) between the two limiting regimes.

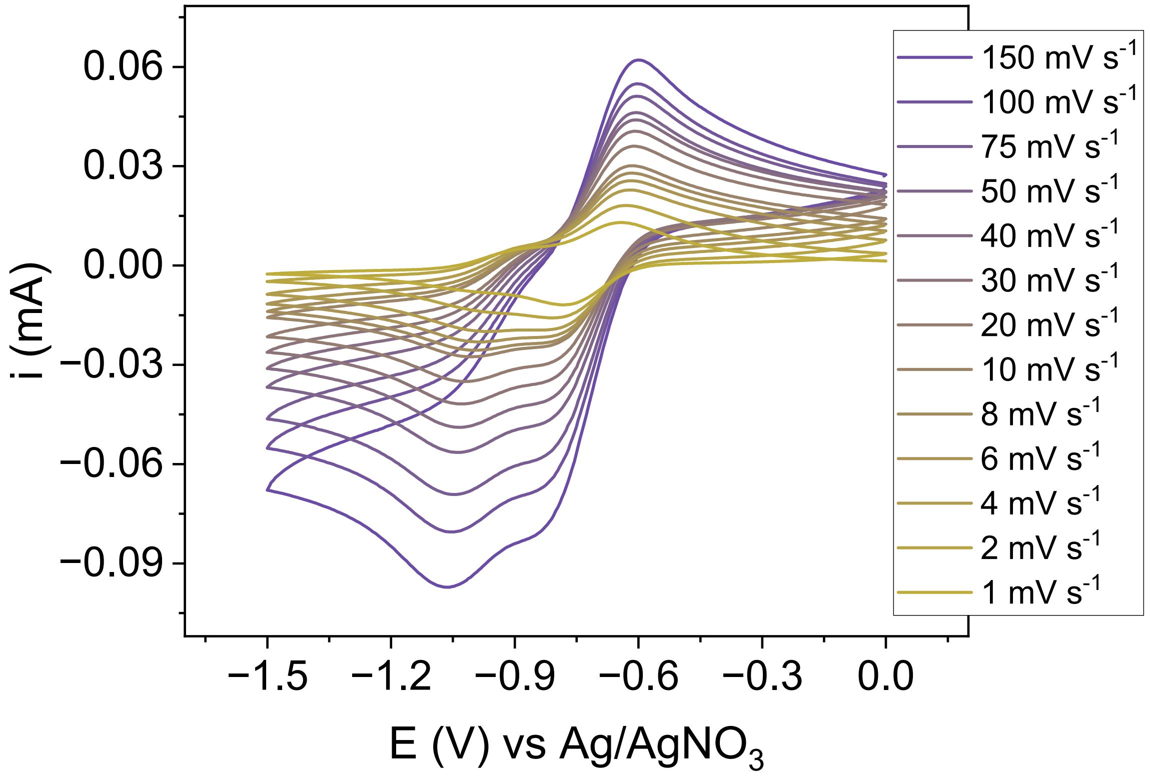

Figure S25. Cyclic voltammograms of TAPT-NDI COF@FTO in Ar-saturated DMF with 0.1 M $NaClO_4$ as supporting electrolyte at various scan rates from 1 to 150 mV s^{-1}.

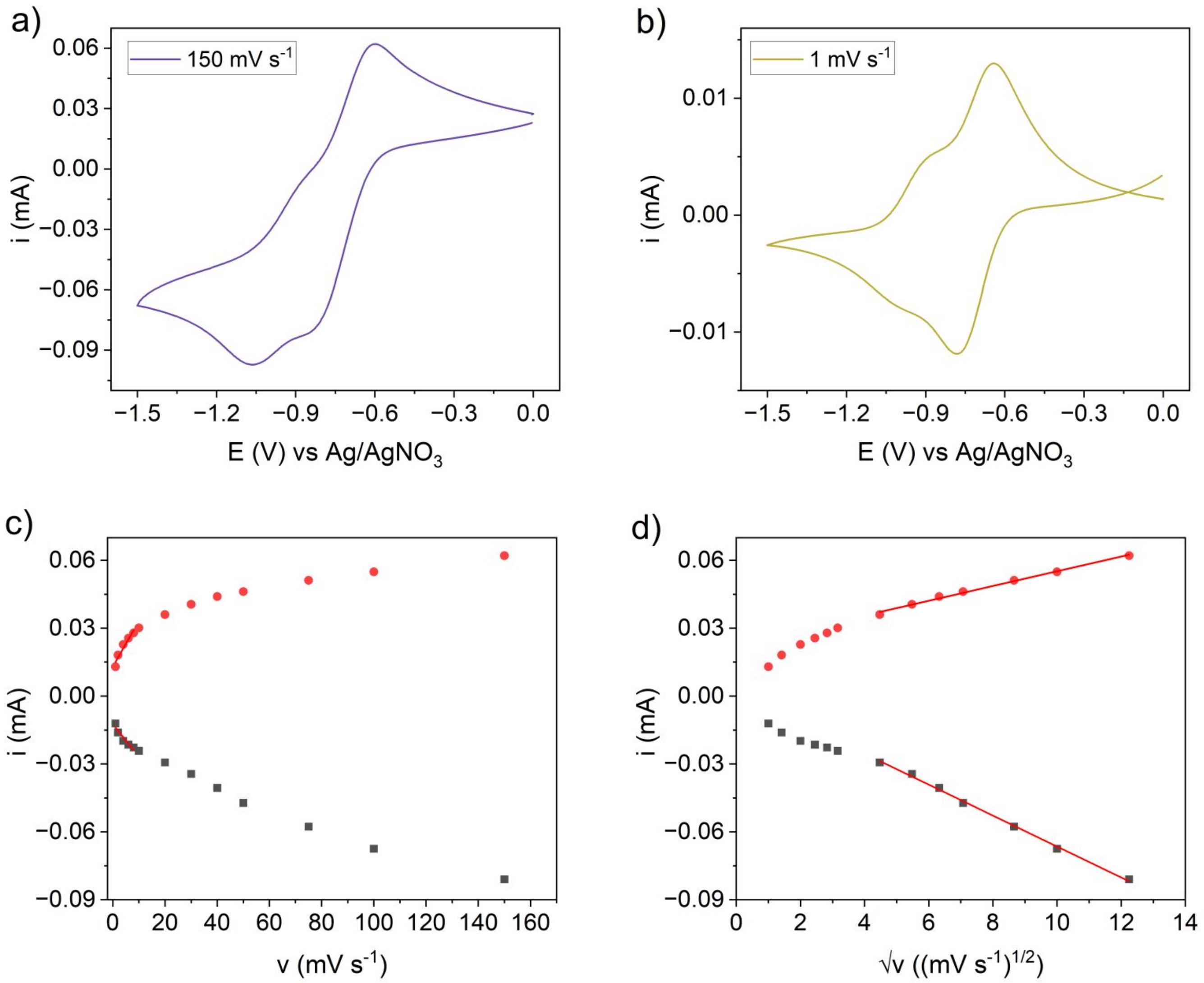

Figure S26. Cyclic voltammograms of TAPT-NDI COF@FTO in Ar-saturated EtOH with 0.1 M $NaClO_4$ as supporting electrolyte at a scan rate of 150 mV s^{-1} (a) and 1 mV s^{-1} (b). The plot of cathodic and anodic peak currents corresponding to the $NDI^{0/\bullet-}$ redox couple, *i* vs. *v* (c) and *i* vs. $v^{-1/2}$ (d), discloses the transition scan rate (10 mV s^{-1}) between the two limiting regimes.

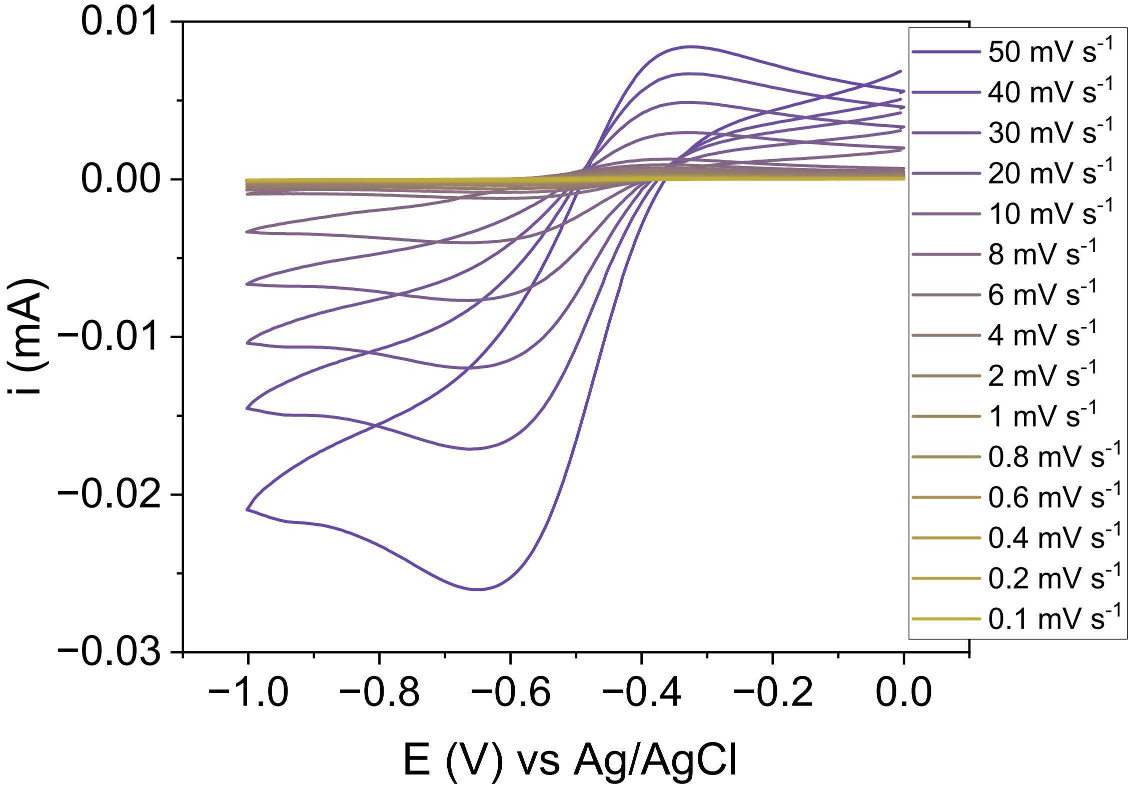

Figure S27. Cyclic voltammograms of TAPT-NDI COF@FTO in Ar-saturated water with 0.1 M LiCl as supporting electrolyte at various scan rates from 0.1 to 50 mV s^{-1}.

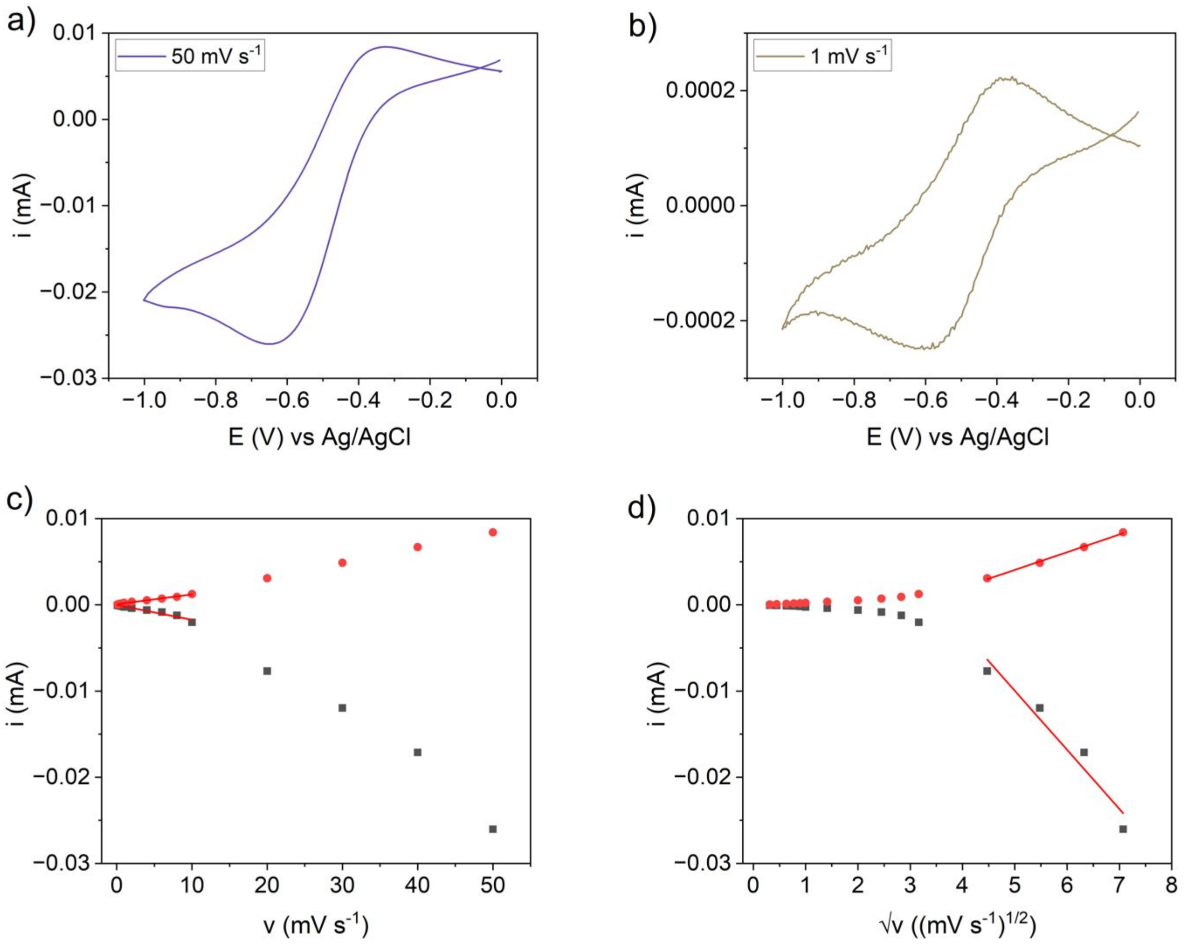

Figure S28. Cyclic voltammograms of TAPT-NDI COF@FTO in Ar-saturated water with 0.1 M LiCl as supporting electrolyte at a scan rate of 50 mV s^{-1} (a) and 1 mV s^{-1} (b). The plot of cathodic and anodic peak currents corresponding to the $NDI^{0/\bullet-}$ redox couple, *i* vs. *v* (c) and *i* vs. $v^{-1/2}$ (d), discloses the transition scan rate (10 mV s^{-1}) between the two limiting regimes.

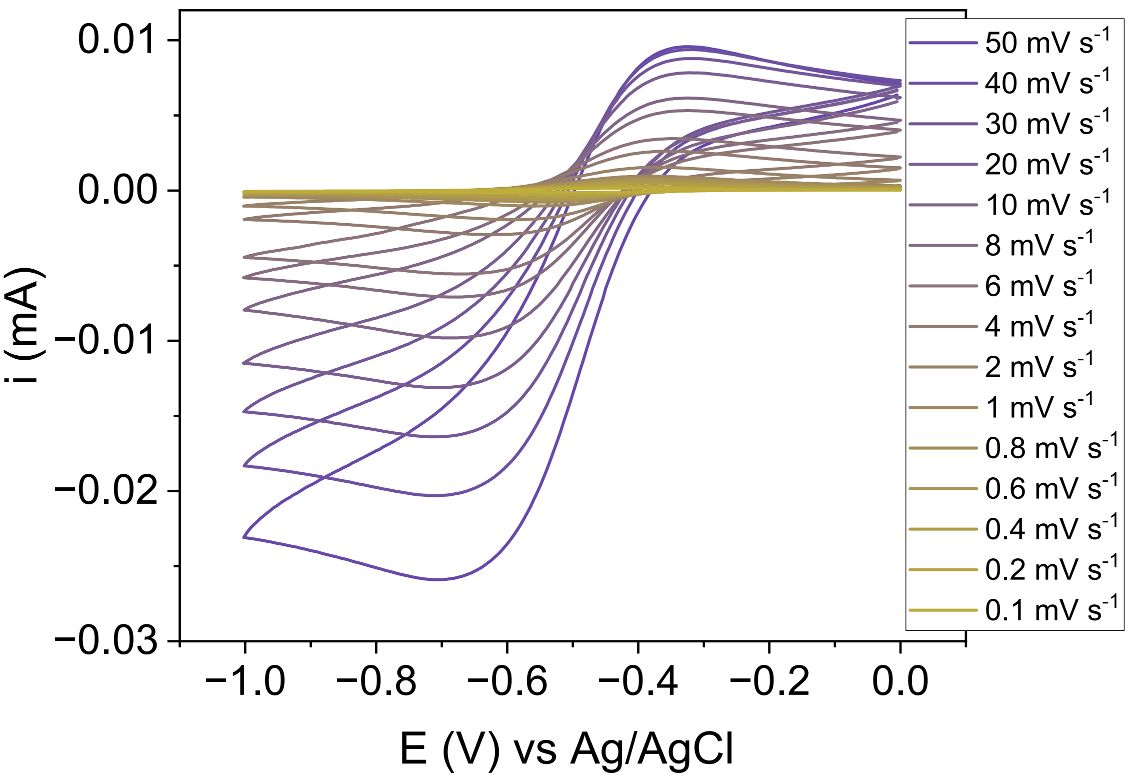

Figure S29. Cyclic voltammograms of TAPT-NDI COF@FTO in Ar-saturated water with 0.1 M NaCl as supporting electrolyte at various scan rates from 0.1 to 50 mV s^{-1}.

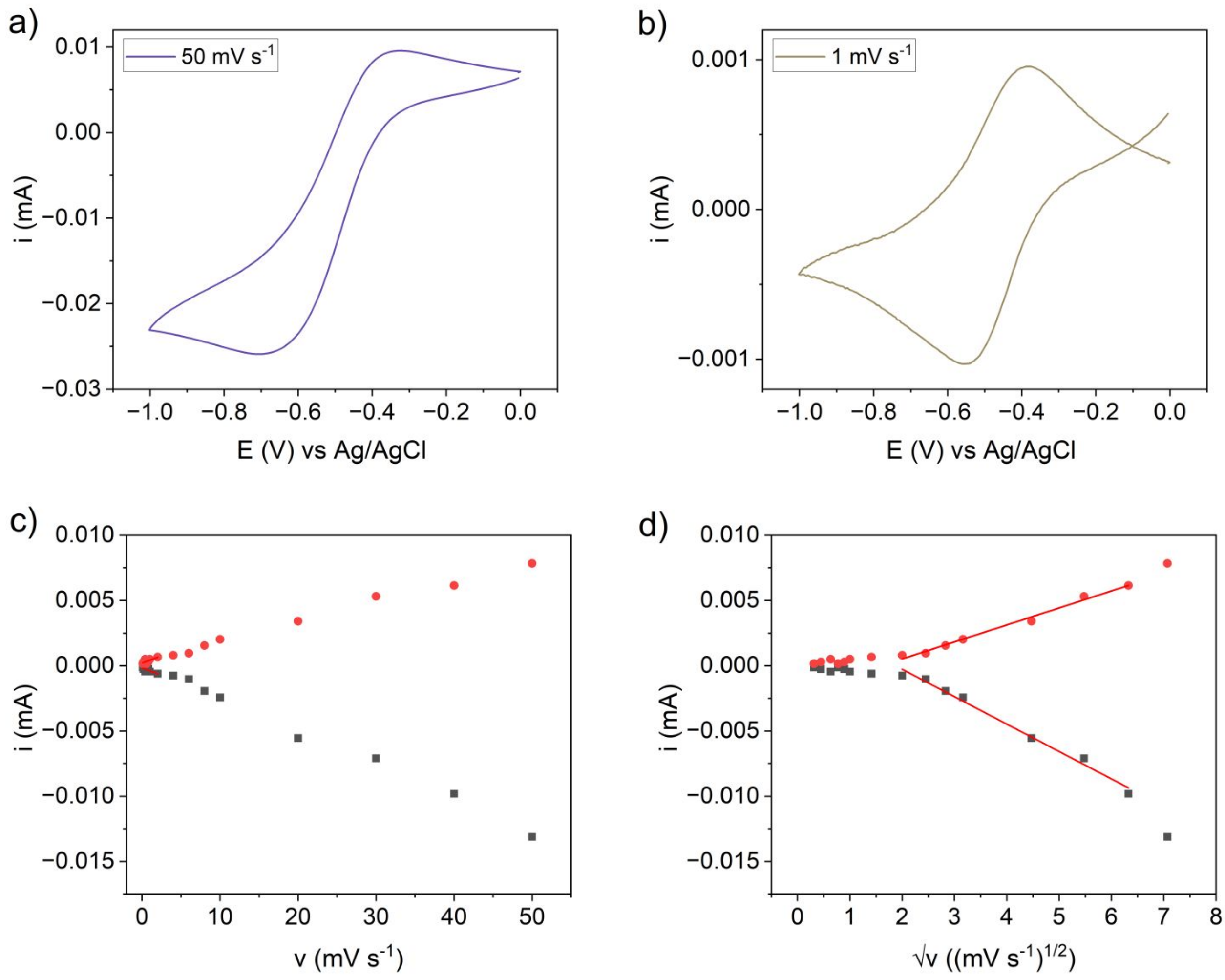

Figure S30. Cyclic voltammograms of TAPT-NDI COF@FTO in Ar-saturated water with 0.1 M NaCl as supporting electrolyte at a scan rate of 50 mV s^{-1} (a) and 1 mV s^{-1} (b). The plot of cathodic and anodic peak currents corresponding to the NDI$^{0/\bullet-}$ redox couple, *i* vs. *v* (c) and *i* vs. $v^{-1/2}$ (d), discloses the transition scan rate (4 mV s^{-1}) between the two limiting regimes.

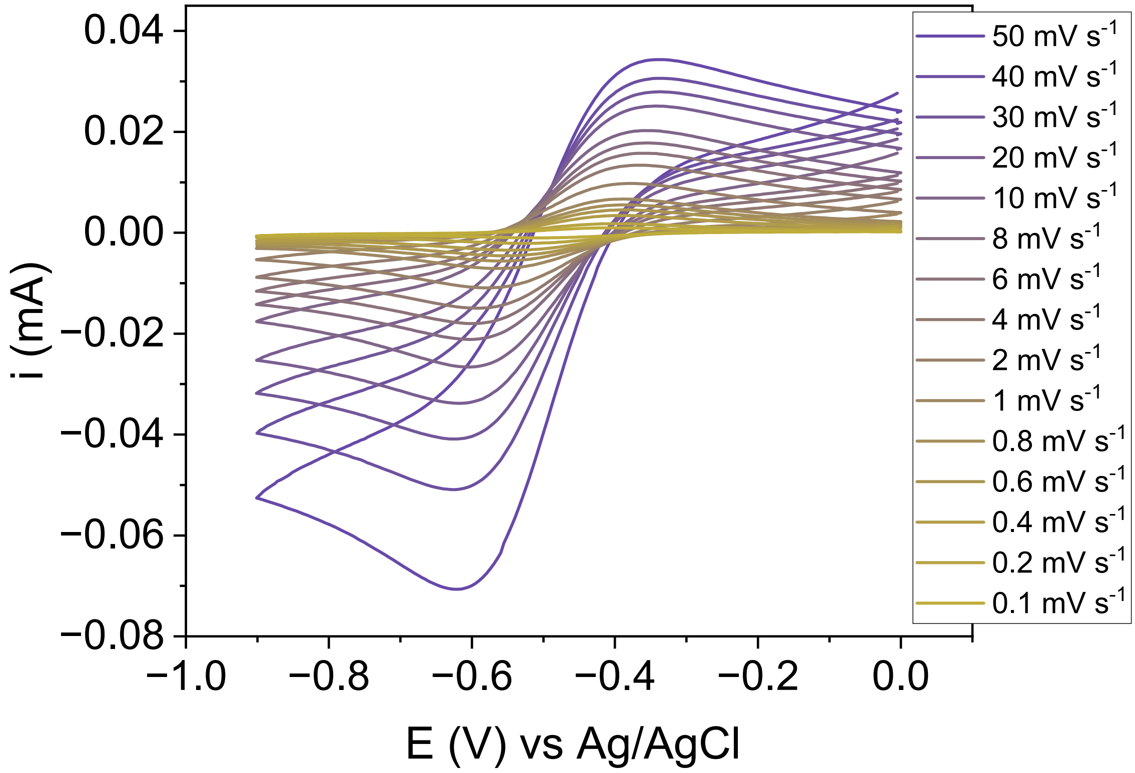

Figure S31. Cyclic voltammograms of TAPT-NDI COF@FTO in Ar-saturated water with 0.1 M KCl as supporting electrolyte at various scan rates from 0.1 to 50 mV s^{-1}.

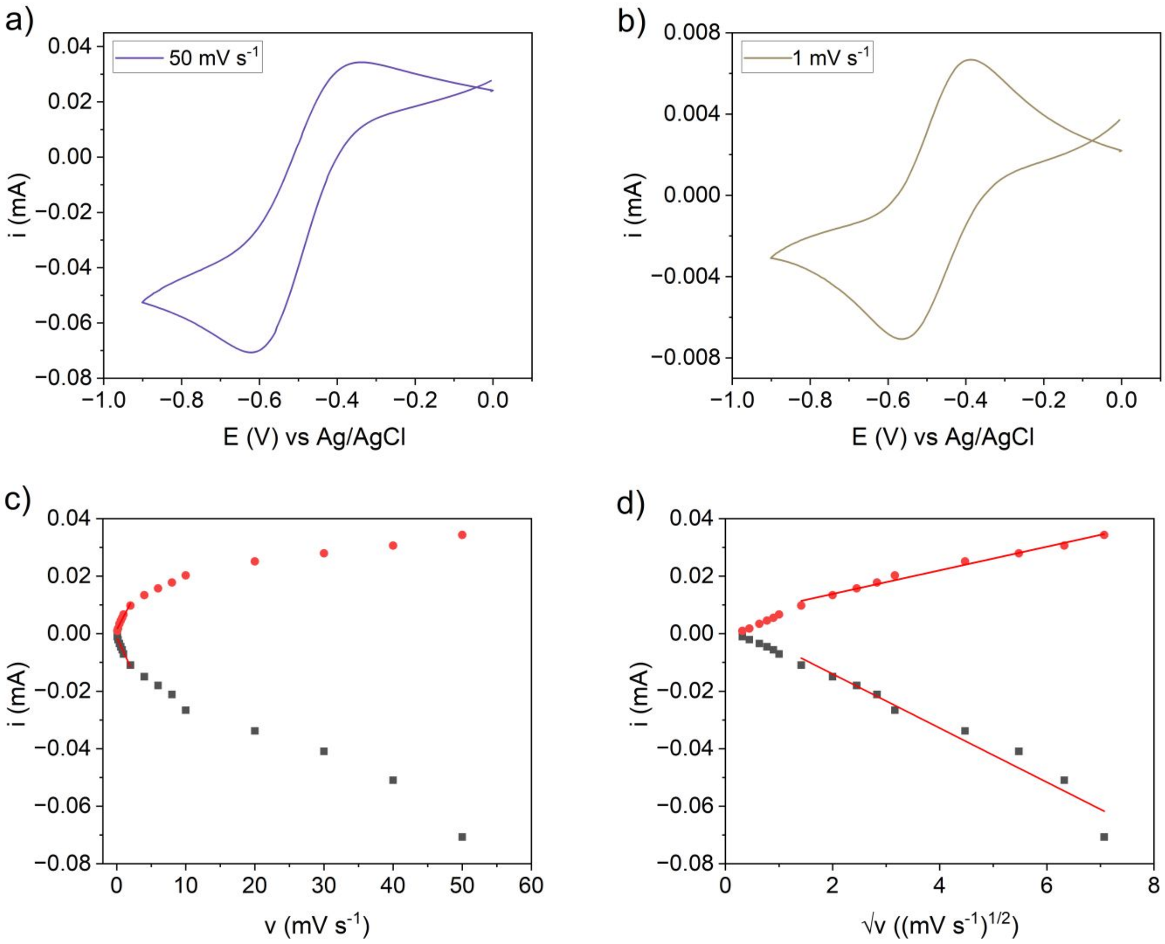

Figure S32. Cyclic voltammograms of TAPT-NDI COF@FTO in Ar-saturated water with 0.1 M KCl as supporting electrolyte at a scan rate of 50 mV s^{-1} (a) and 1 mV s^{-1} (b). The plot of cathodic and anodic peak currents corresponding to the $NDI^{0/\bullet-}$ redox couple, i vs. v (c) and i vs. $v^{-1/2}$ (d), discloses the transition scan rate (1 mV s^{-1}) between the two limiting regimes.

Table S1. Selected parameters for the solvents used electrochemical experiments.

Solvent	Relative polarity	Donor number (kcal/mol)	Dielectric constant	Dipole moment (D)
Acetonitrile	0.46	14.10	36.64	3.92
DMF	0.38	26.60	38.25	3.82
Ethanol	0.65	19.20	24.60	1.69
Water	1.00	18.00	78.54	1.85

Calculation of electroactive concentration (Γ_e) and apparent diffusion coefficient (D_e^{app})

Chronoamperometry was employed to calculate the electroactive concentration of NDI linkers in each COF film and the apparent diffusion coefficient (D_e^{app}) in the different electrolyte/solvent combinations. After applying a potential in the non-faradaic region for 120 s, the potential was stepped to a suitable value (more negative than $NDI^{0/\bullet-}$ peak chosen from the CV curve) and held for 900 s to reduce the linkers to the $NDI^{\bullet-}$ radical anion. To determine the total charge passed for the exhaustive reduction of the film, the current density (j) and recorded charge (Q) from this potential step were plotted vs. time. A residual background current was subtracted to estimate the total charge passed to reduce accessible NDI linkers.

The electroactive NDI concentration, Γ_e (mol cm^{-2}) was calculated according to the following equation:

$$\Gamma_e = \frac{|Q|}{n\,F\,S_A} \quad \text{(eq S1)}$$

where Q is the charge passed (in C) after exhaustive reduction of the film, n is the number of electrons transferred per redox-active species, F is Faraday's constant, and S_A (in cm^{-2}) is the geometric surface area of the TAPT-NDI COF@FTO electrode.

To determine D_e^{app} for electron-hopping diffusion, the time-dependent current density j(t) was fitted to the Cottrell equation:

$$j(t) = \frac{nF\Gamma_e\sqrt{D_e^{app}}}{d_f\sqrt{\pi t}} \quad \text{(eq S2)}$$

where d_f is the COF film thickness (in cm^2) and D_e^{app} is the diffusion coefficient (in cm^2 s^{-1}). During short time transients in the plot of j(t) vs. t$^{-1/2}$, a linear region was selected to calculate D_e^{app} according the following equation:

$$D_e^{app} = \left(\frac{Slope * d_f\sqrt{\pi}}{nF\Gamma_e}\right)^2 \quad \text{(eq S3)}$$

All parameters involved in the experimental calculation of Γ_e and D_e^{app} for individual films measured in different electrolyte-solvent combinations are tabulated in Tables S2–S12 below the respective chronoamperometry/chronocoulometry measurements and Cottrell analysis.

Estimation of TAPT-NDI COF film thickness

To determine the thickness of the film (d_f) correctly, the TAPT-NDI COF@FTO electrodes were cut into half after the electrochemical measurements and cross-sectional SEM images were taken.

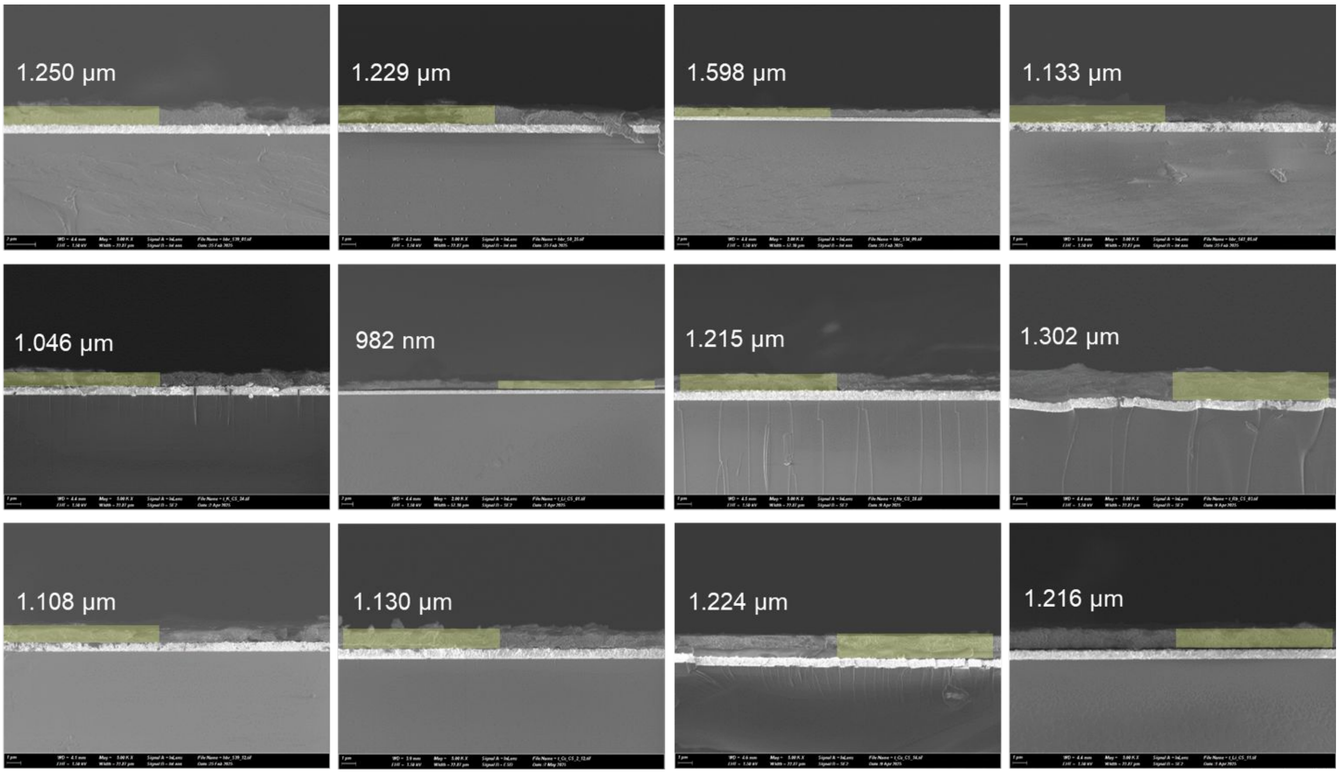

Figure S33. Representative cross-section SEM images of TAPT-NDI COF film on FTO after electrochemical measurements. The numbers indicate the film thickness.

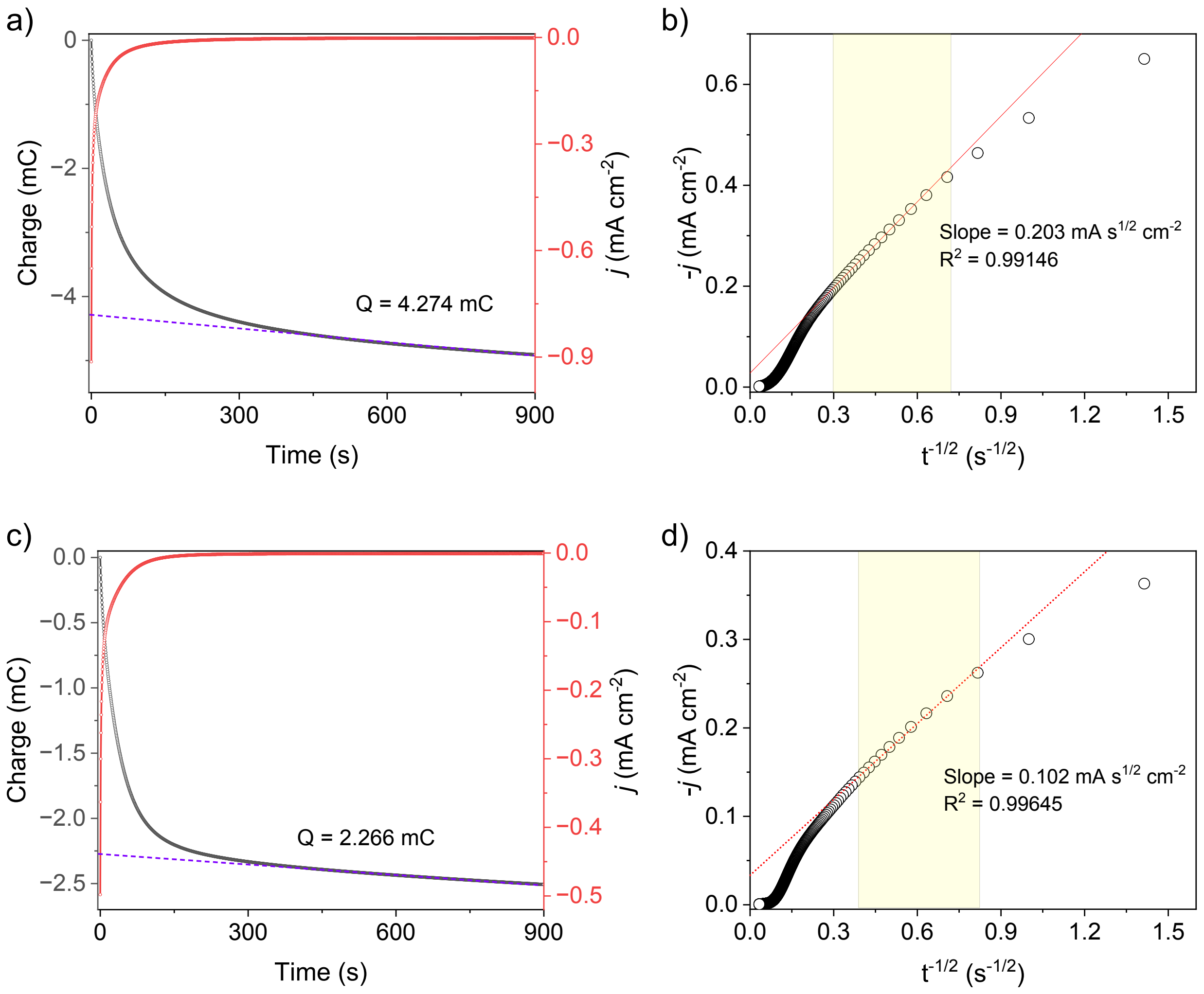

Figure S34. Representative electrochemical data for TAPT-NDI COF@FTO samples measured in 0.1 M $LiClO_4$ in acetonitrile. (a, c) Chronoamperometry (solid red line) and chronocoulometry (solid black line) after stepping the potential −0.3 V → −0.9 V vs Ag/$AgNO_3$ (selected from the CV) to isolate the $NDI^{0/\bullet-}$ redox couple. The total charge passed after complete reduction was estimated by subtracting a residual background current (blue dashed line). (b, d) Corresponding Cottrell plots of COF film samples after potential step. Yellow boxes show the data range selected for linear fits.

Table S2. Selected parameters and experimentally measured Γ_e and D_e^{app} for $LiClO_4$ in MeCN

Electrode	S_A (cm^{-2})	d_f (cm)	Q (C)	Cottrell slope	Γ_e (mol cm^{-2})	D_e^{app} (cm^2 s^{-1})
1	0.562	1.195E-4	0.00427	2.03E-4	7.88201E-8	3.19493E-11
2	0.567	1.208E-4	0.00227	1.02E-4	4.14205E-8	2.98477E-11
3	0.559	9.162E-5	0.00266	1.43E-4	4.92442E-8	2.38754E-11
4	0.586	9.810E-5	0.00369	1.06E-4	6.52631E-8	8.56292E-12

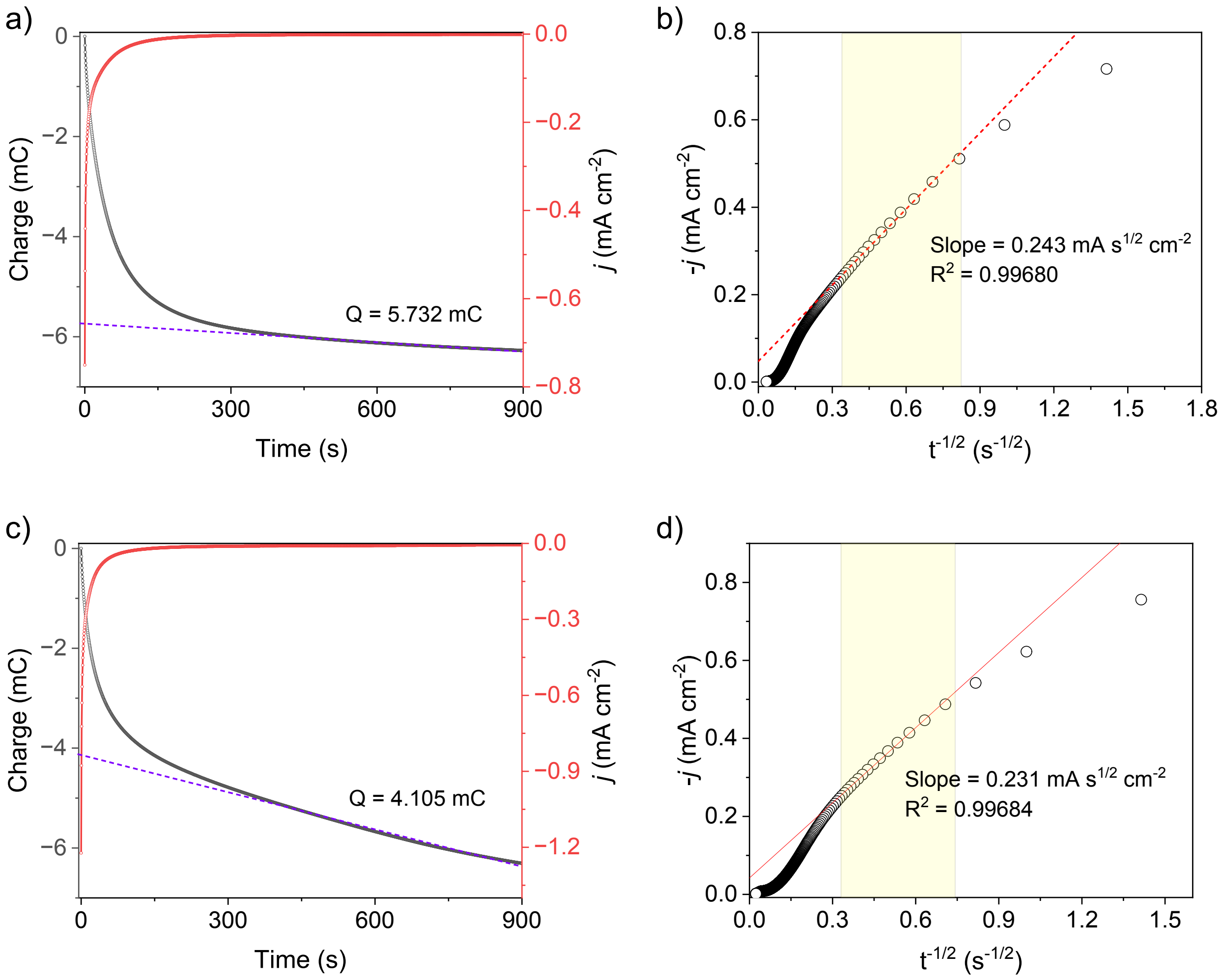

Figure S35. Representative electrochemical data for TAPT-NDI COF@FTO samples measured in 0.1 M $NaClO_4$ in acetonitrile. (a, c) Chronoamperometry (solid red line) and chronocoulometry (solid black line) after stepping the potential −0.3 V → −0.98 V vs Ag/$AgNO_3$ (selected from the CV) to isolate the $NDI^{0/\bullet-}$ redox couple. The total charge passed after complete reduction was estimated by subtracting a residual background current (blue dashed line). (b, d) Corresponding Cottrell plots of COF film samples after potential step. Yellow boxes show the data range selected for linear fits.

Table S3. Selected parameters and experimentally measured Γ_e and D_e^{app} for $NaClO_4$ in MeCN

Electrode	S_A (cm^{-2})	d_f (cm)	Q (C)	Cottrell slope	Γ_e (mol cm^{-2})	D_e^{app} (cm^2 s^{-1})
1	0.464	9.460E-5	0.00573	2.43E-4	1.28034E-7	1.0873E-11
2	0.562	1.302E-4	0.00411	2.31E-4	7.57034E-8	5.3238E-11
3	0.558	9.573E-5	0.00390	3.13E-4	7.23641E-8	5.7829E-11
4	0.486	1.182E-4	0.00341	2.36E-4	7.26992E-8	4.9660E-11

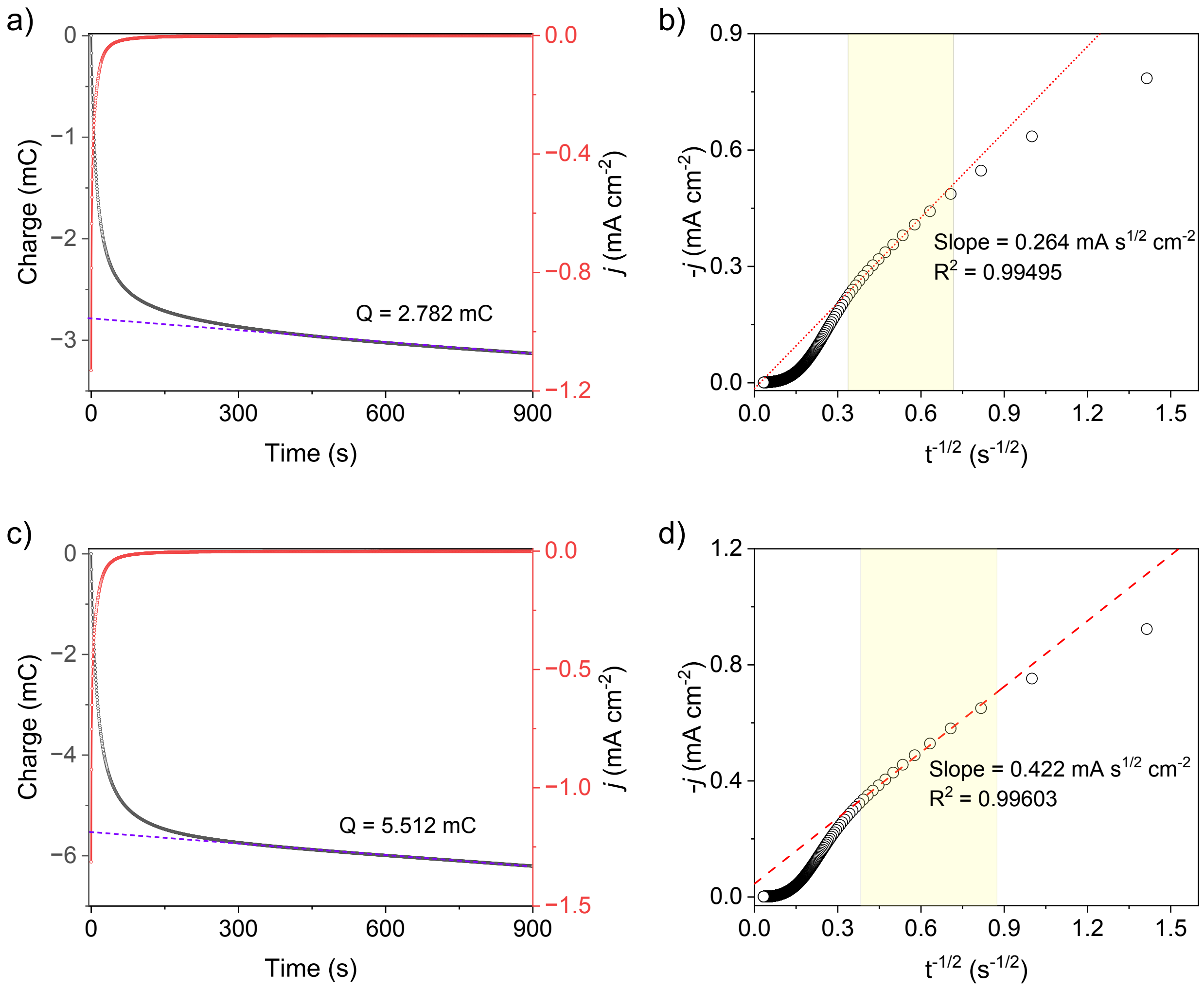

Figure S36. Representative electrochemical data for TAPT-NDI COF@FTO samples measured in 0.1 M KPF_6 in acetonitrile. (a, c) Chronoamperometry (solid red line) and chronocoulometry (solid black line) after stepping the potential −0.3 V → −1.05 V vs Ag/$AgNO_3$ (selected from the CV) to isolate the $NDI^{0/•−}$ redox couple. The total charge passed after complete reduction was estimated by subtracting a residual background current (blue dashed line). (b, d) Corresponding Cottrell plots of COF film samples after potential step. Yellow boxes show the data range selected for linear fits.

Table S4. Selected parameters and experimentally measured Γ_e and D_e^{app} for KPF_6 in MeCN

Electrode	S_A (cm^{-2})	d_f (cm)	Q (C)	Cottrell slope	Γ_e (mol cm^{-2})	D_e^{app} (cm^2 s^{-1})
1	0.578	1.086E-4	0.00278	2.64E-4	4.98848E-8	1.11414E-10
2	0.573	9.720E-5	0.00551	4.22E-4	9.96996E-8	5.70923E-11
3	0.586	1.127E-4	0.00335	2.93E-4	5.92320E-8	1.04828E-10
4	0.554	1.093E-4	0.00498	3.07E-4	9.31849E-8	4.37354E-11

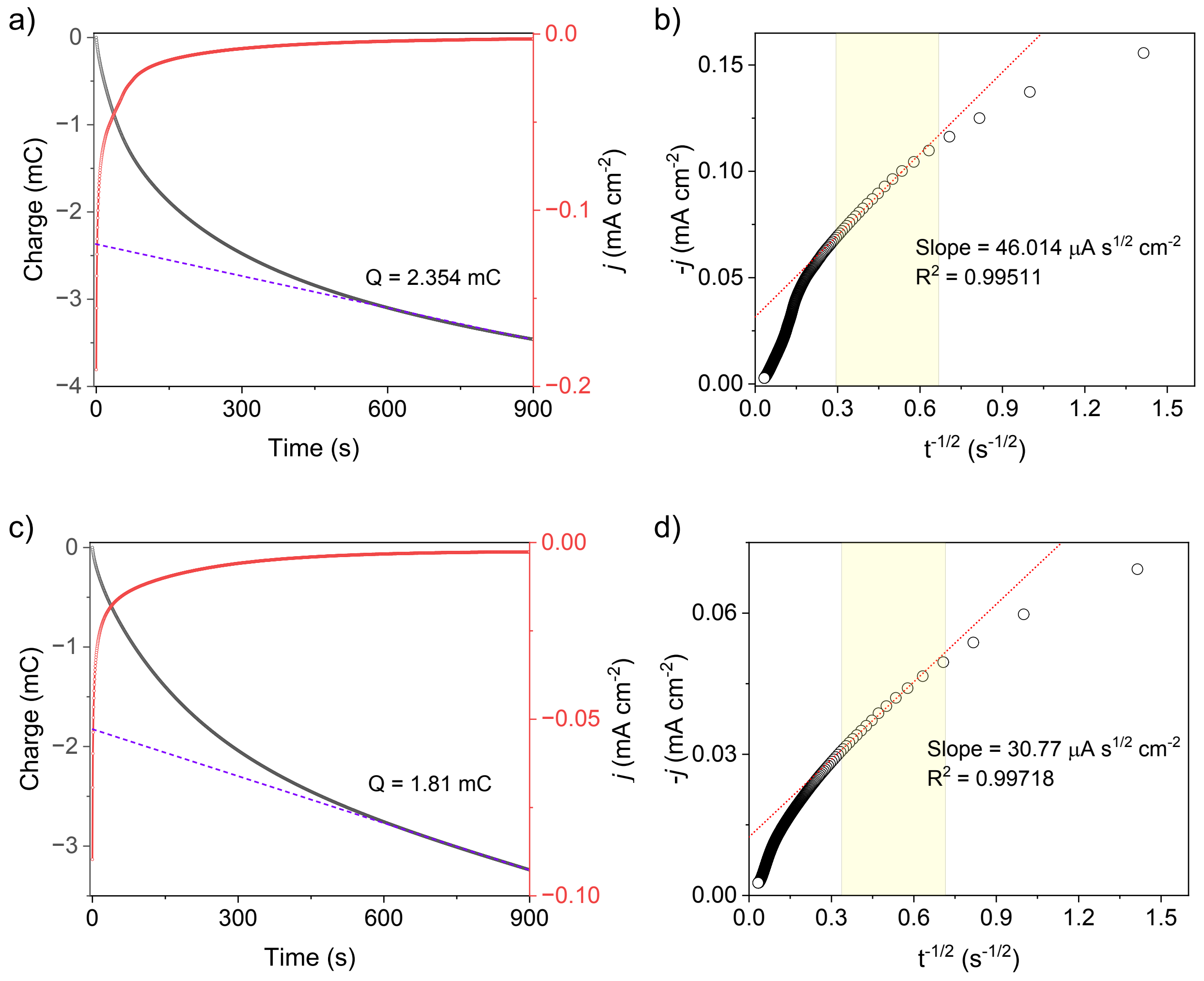

Figure S37. Representative electrochemical data for TAPT-NDI COF@FTO samples measured in 0.1 M $LiClO_4$ in DMF. (a, c) Chronoamperometry (solid red line) and chronocoulometry (solid black line) after stepping the potential −0.4 V → −1.18 V vs $Ag/AgNO_3$ (selected from the CV) to isolate the $NDI^{0/\bullet-}$ redox couple. The total charge passed after complete reduction was estimated by subtracting a residual background current (blue dashed line). (b, d) Corresponding Cottrell plots of COF film samples after potential step. Yellow boxes show the data range selected for linear fits.

Table S5. Selected parameters and experimentally measured Γ_e and D_e^{app} for $LiClO_4$ in DMF

Electrode	S_A (cm^{-2})	d_f (cm)	Q (C)	Cottrell slope	Γ_e (mol cm^{-2})	D_e^{app} (cm^2 s^{-1})
1	0.458	9.161E-5	0.00235	4.6E-5	5.32696E-8	2.11035E-12
2	0.556	9.720E-5	0.00181	3.1E-5	3.37771E-8	2.68422E-12
3	0.526	1.122E-4	0.00264	7.2E-5	5.20775E-8	8.11629E-12
4	0.528	1.021E-4	0.00197	5.2E-5	3.87090E-8	6.34515E-12

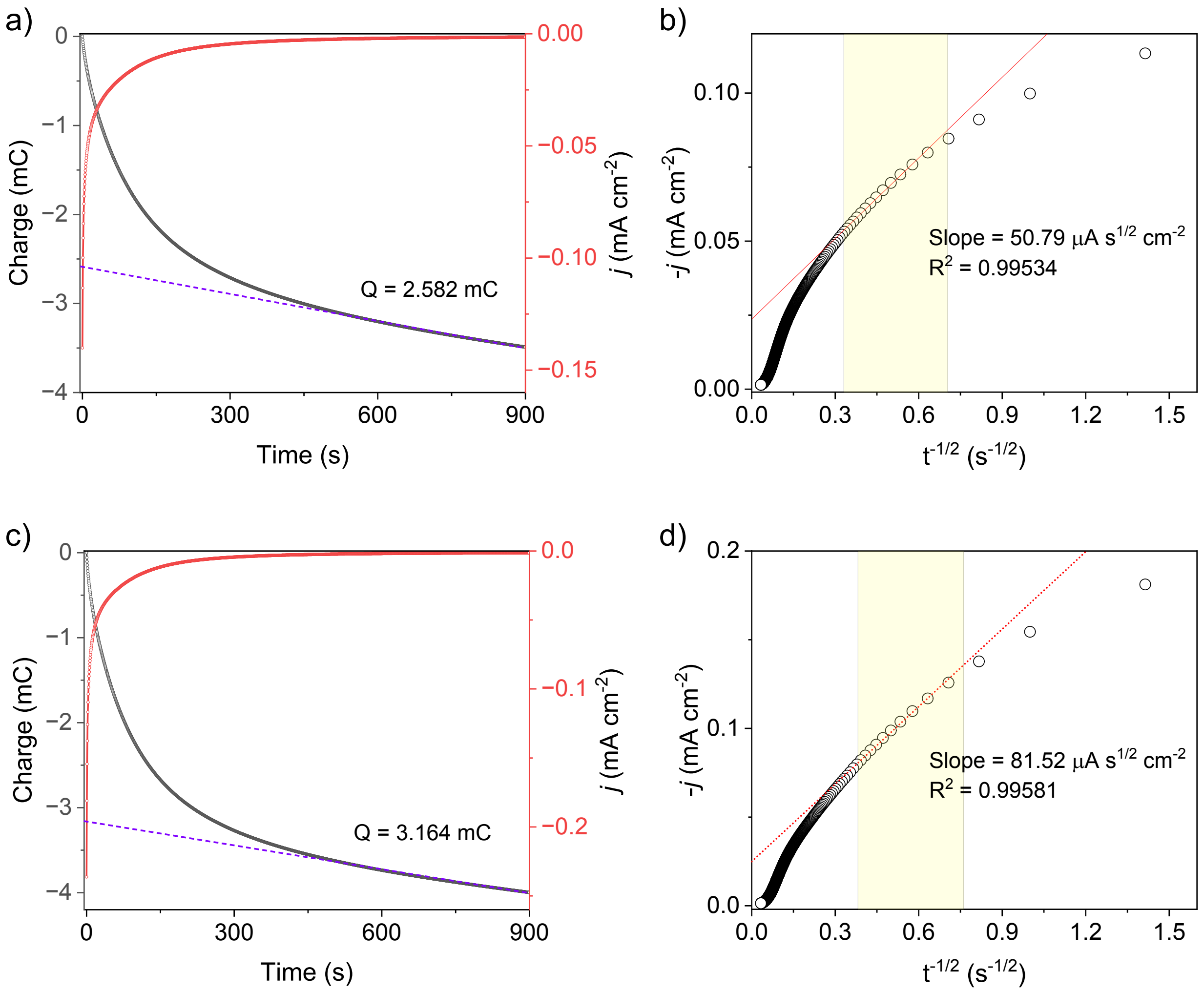

Figure S38. Representative electrochemical data for TAPT-NDI COF@FTO samples measured in 0.1 M $NaClO_4$ in DMF. (a, c) Chronoamperometry (solid red line) and chronocoulometry (solid black line) after stepping the potential −0.4 V → −1.21 V vs Ag/$AgNO_3$ (selected from the CV) to isolate the $NDI^{0/\bullet-}$ redox couple. The total charge passed after complete reduction was estimated by subtracting a residual background current (blue dashed line). (b, d) Corresponding Cottrell plots of COF film samples after potential step. Yellow boxes show the data range selected for linear fits.

Table S6. Selected parameters and experimentally measured Γ_e and D_e^{app} for $NaClO_4$ in DMF

Electrode	S_A (cm^{-2})	d_f (cm)	Q (C)	Cottrell slope	Γ_e (mol cm^{-2})	D_e^{app} (cm^2 s^{-1})
1	0.567	9.161E-5	0.00258	5.1E-5	4.71967E-8	3.30457E-12
2	0.556	9.722E-5	0.00316	8.1E-5	5.89794E-8	6.01048E-12
3	0.494	1.108E-4	0.00308	1.06E-4	6.46613E-8	1.11278E-11
4	0.524	1.229E-4	0.00401	1.12E-4	7.93737E-8	1.01436E-11

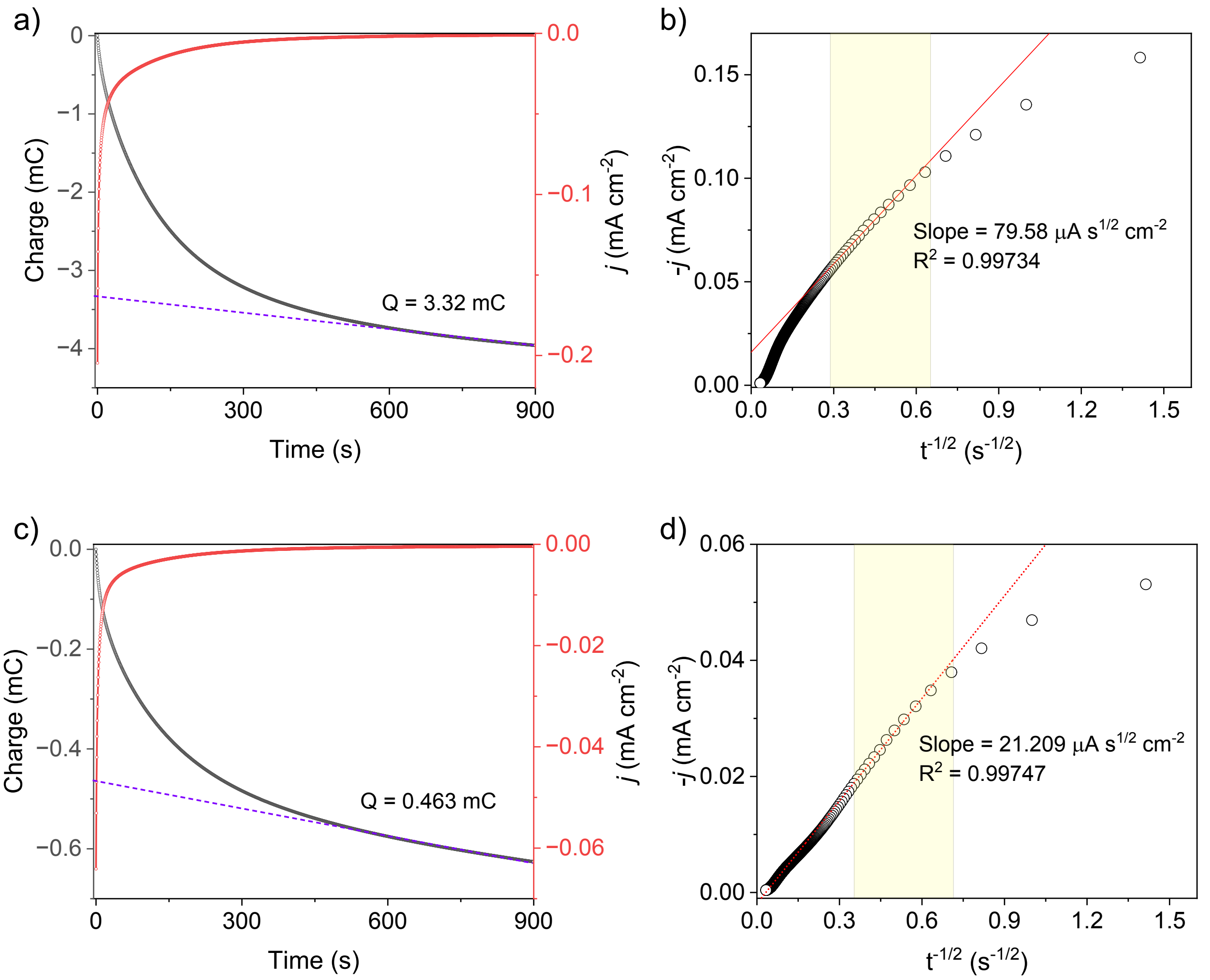

Figure S39. Representative electrochemical data for TAPT-NDI COF@FTO samples measured in 0.1 M $KClO_4$ in DMF. (a, c) Chronoamperometry (solid red line) and chronocoulometry (solid black line) after stepping the potential −0.4 V → −1.23 V vs Ag/$AgNO_3$ (selected from the CV) to isolate the $NDI^{0/\bullet-}$ redox couple. The total charge passed after complete reduction was estimated by subtracting a residual background current (blue dashed line). (b, d) Corresponding Cottrell plots of COF film samples after potential step. Yellow boxes show the data range selected for linear fits.

Table S7. Selected parameters and experimentally measured Γ_e and D_e^{app} for $KClO_4$ in DMF

Electrode	S_A (cm^{-2})	d_f (cm)	Q (C)	Cottrell slope	Γ_e (mol cm^{-2})	D_e^{app} (cm^2 s^{-1})
1	0.564	9.16E-5	0.00332	7.9E-5	6.10279E-8	4.74237E-12
2	0.468	9.72E-5	4.63E-4	2.1E-5	1.02535E-8	1.33669E-11
3	0.492	8.79E-5	0.00188	3.7E-5	3.9519E-8	2.28442E-12
4	0.474	9.72E-5	0.00207	8.9E-5	4.53273E-8	1.22857E-11

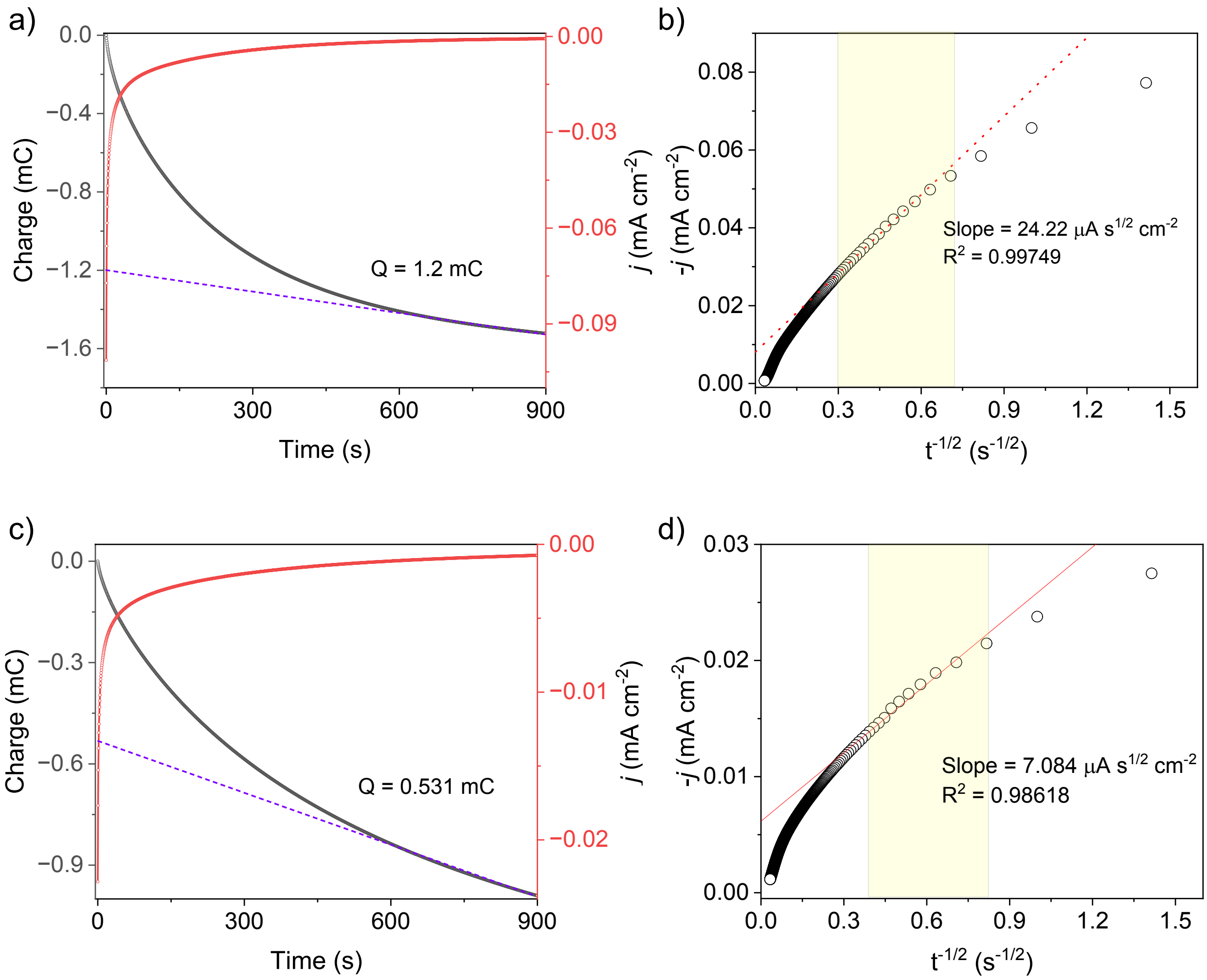

Figure S40. Representative electrochemical data for TAPT-NDI COF@FTO samples measured in 0.1 M $LiClO_4$ in EtOH. (a, c) Chronoamperometry (solid red line) and chronocoulometry (solid black line) after stepping the potential −0.4 V → −0.86 V vs $Ag/AgNO_3$ (selected from the CV) to isolate the $NDI^{0/\bullet-}$ redox couple. The total charge passed after complete reduction was estimated by subtracting a residual background current (blue dashed line). (b, d) Corresponding Cottrell plots of COF film samples after potential step. Yellow boxes show the data range selected for linear fits.

Table S8. Selected parameters and experimentally measured Γ_e and D_e^{app} for $LiClO_4$ in ethanol

Electrode	S_A (cm^{-2})	d_f (cm)	Q (C)	Cottrell slope	Γ_e (mol cm^{-2})	D_e^{app} (cm^2 s^{-1})
1	0.561	9.16E-5	0.00121	2.432E-5	2.23358E-8	3.35522E-12
2	0.568	9.72E-5	5.31E-4	7.21E-6	9.68913E-9	1.76458E-12
3	0.422	1.133E-4	0.00167	2.346E-5	4.11132E-8	1.40981E-12
4	0.538	9.84E-5	0.00112	3.045E-5	2.15184E-8	6.53962E-12

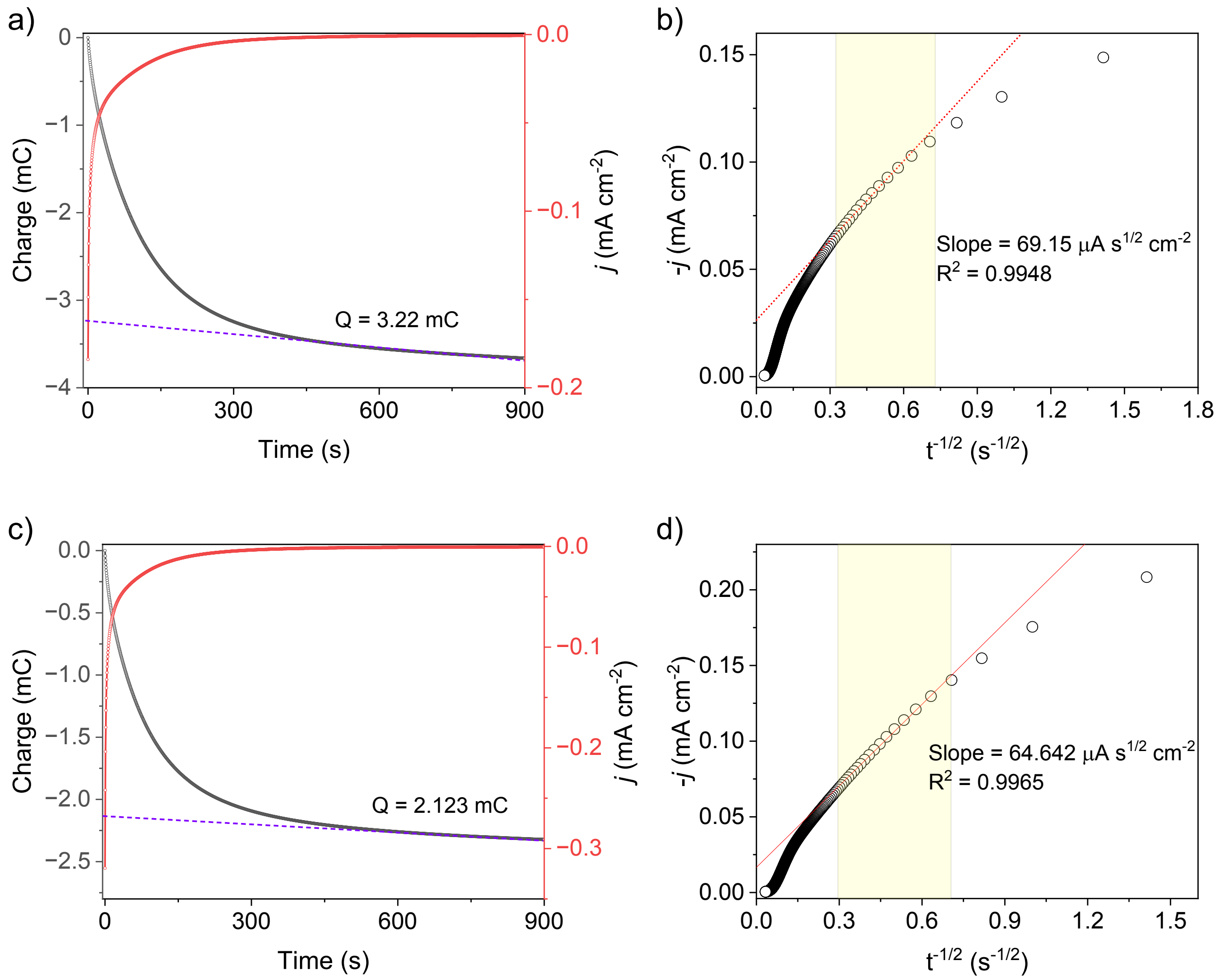

Figure S41. Representative electrochemical data for TAPT-NDI COF@FTO samples measured in 0.1 M $NaClO_4$ in EtOH. (a, c) Chronoamperometry (solid red line) and chronocoulometry (solid black line) after stepping the potential −0.4 V → −0.91 V vs Ag/$AgNO_3$ (selected from the CV) to isolate the $NDI^{0/\bullet-}$ redox couple. The total charge passed after complete reduction was estimated by subtracting a residual background current (blue dashed line). (b, d) Corresponding Cottrell plots of COF film samples after potential step. Yellow boxes show the data range selected for linear fits.

Table S9. Selected parameters and experimentally measured Γ_e and D_e^{app} for $NaClO_4$ in ethanol

Electrode	S_A (cm^{-2})	d_f (cm)	Q (C)	Cottrell slope	Γ_e (mol cm^{-2})	D_e^{app} (cm^2 s^{-1})
1	0.565	9.16E-5	0.00322	6.9192E-5	5.90855E-8	3.88104E-12
2	0.368	9.72E-5	0.00212	6.4512E-5	5.97917E-8	3.7097E-12
3	0.443	1.046E-4	0.00252	7.567E-5	5.90038E-8	6.06957E-12
4	0.515	9.69E-5	0.00305	9.1812E-5	6.13404E-8	7.09513E-12

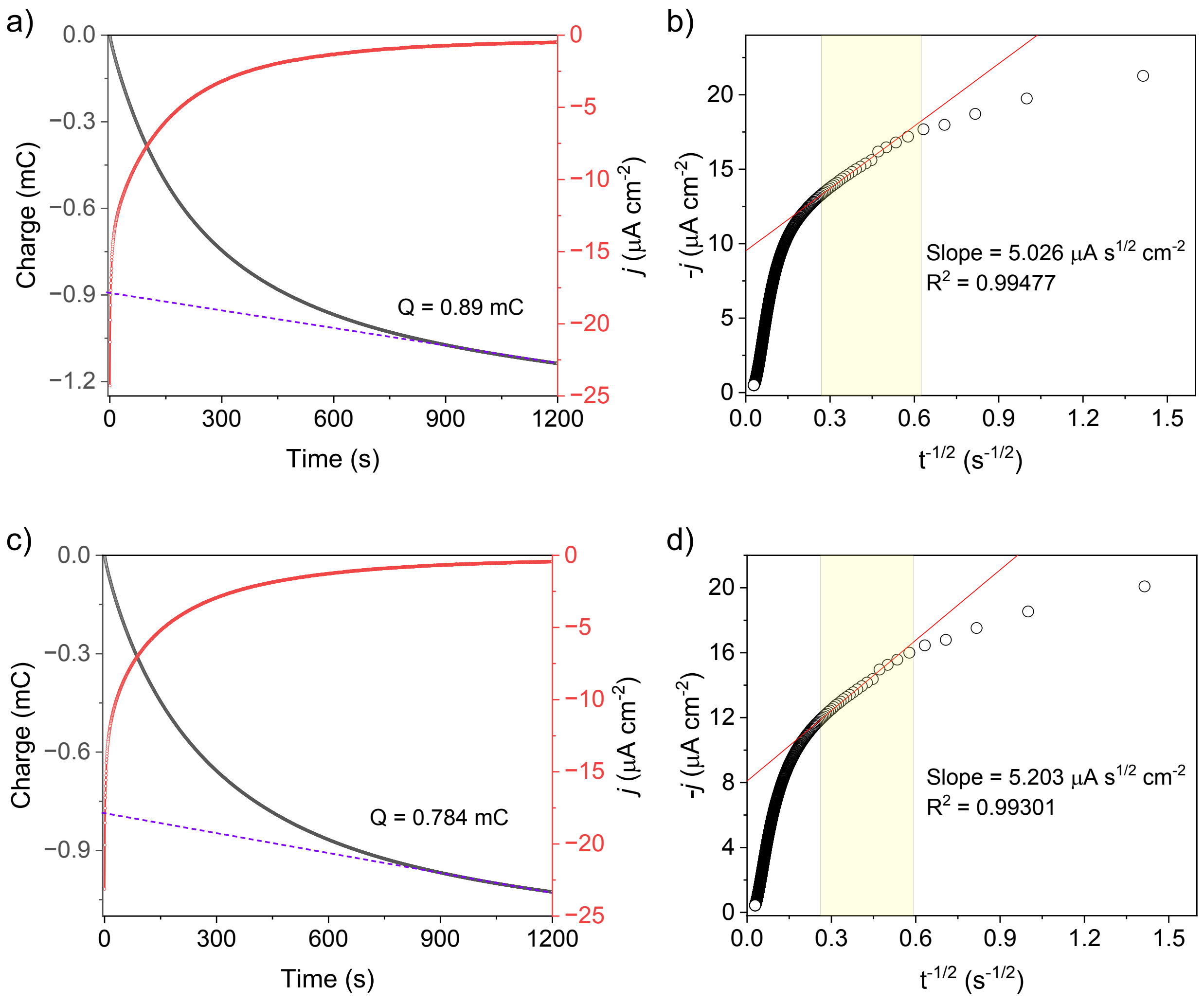

Figure S42. Representative electrochemical data for TAPT-NDI COF@FTO samples measured in 0.1 M LiCl in water. (a, c) Chronoamperometry (solid red line) and chronocoulometry (solid black line) after stepping the potential −0.2 V → −0.45 V vs Ag/AgCl (selected from the CV) to isolate the $NDI^{0/\bullet-}$ redox couple. The total charge passed after complete reduction was estimated by subtracting a residual background current (blue dashed line). (b, d) Corresponding Cottrell plots of COF film samples after potential step. Yellow boxes show the data range selected for linear fits.

Table S10. Selected parameters and experimentally measured Γ_e and D_e^{app} for LiCl in water

Electrode	S_A (cm^{-2})	d_f (cm)	Q (C)	Cottrell slope	Γ_e (mol cm^{-2})	D_e^{app} (cm^2 s^{-1})
1	0.414	1.216E-4	8.91E-4	5.026E-6	2.23057E-8	2.53214E-13
2	0.468	9.62E-5	7.84E-4	5.218E-6	1.73624E-8	2.81934E-13
3	0.452	1.107E-4	0.00113	1.004E-5	2.59336E-8	6.19504E-13
4	0.524	1.018E-4	0.00105	9.126E-6	2.08077E-8	6.72384E-13

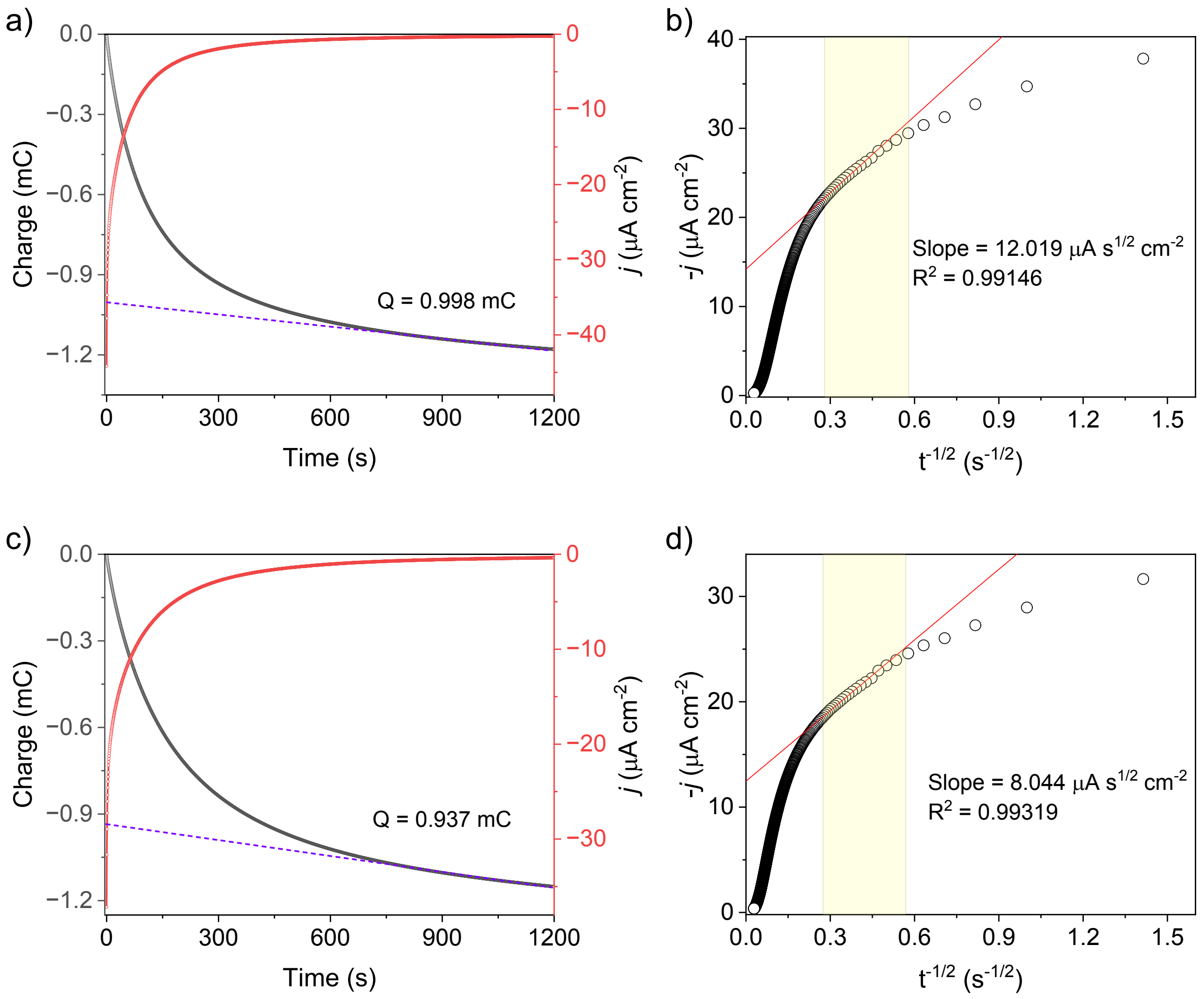

Figure S43. Representative electrochemical data for TAPT-NDI COF@FTO samples measured in 0.1 M NaCl in water. (a, c) Chronoamperometry (solid red line) and chronocoulometry (solid black line) after stepping the potential −0.2 V → −0.45 V vs Ag/AgCl (selected from the CV) to isolate the $NDI^{0/\bullet-}$ redox couple. The total charge passed after complete reduction was estimated by subtracting a residual background current (blue dashed line). (b, d) Corresponding Cottrell plots of COF film samples after potential step. Yellow boxes show the data range selected for linear fits.

Table S11. Selected parameters and experimentally measured Γ_e and D_e^{app} for NaCl in water

Electrode	S_A (cm^{-2})	d_f (cm)	Q (C)	Cottrell slope	Γ_e (mol cm^{-2})	D_e^{app} (cm^2 s^{-1})
1	0.424	1.212E-4	9.98E-4	1.2019E-5	2.43951E-8	1.20266E-12
2	0.367	9.822E-5	9.37E-4	8.044E-6	2.64614E-8	3.00695E-13
3	0.414	9.246E-5	6.126E-4	9.102E-6	1.53361E-8	1.01568E-12
4	0.438	8.64E-5	3.106E-4	6.02E-6	7.35011E-9	1.68904E-12

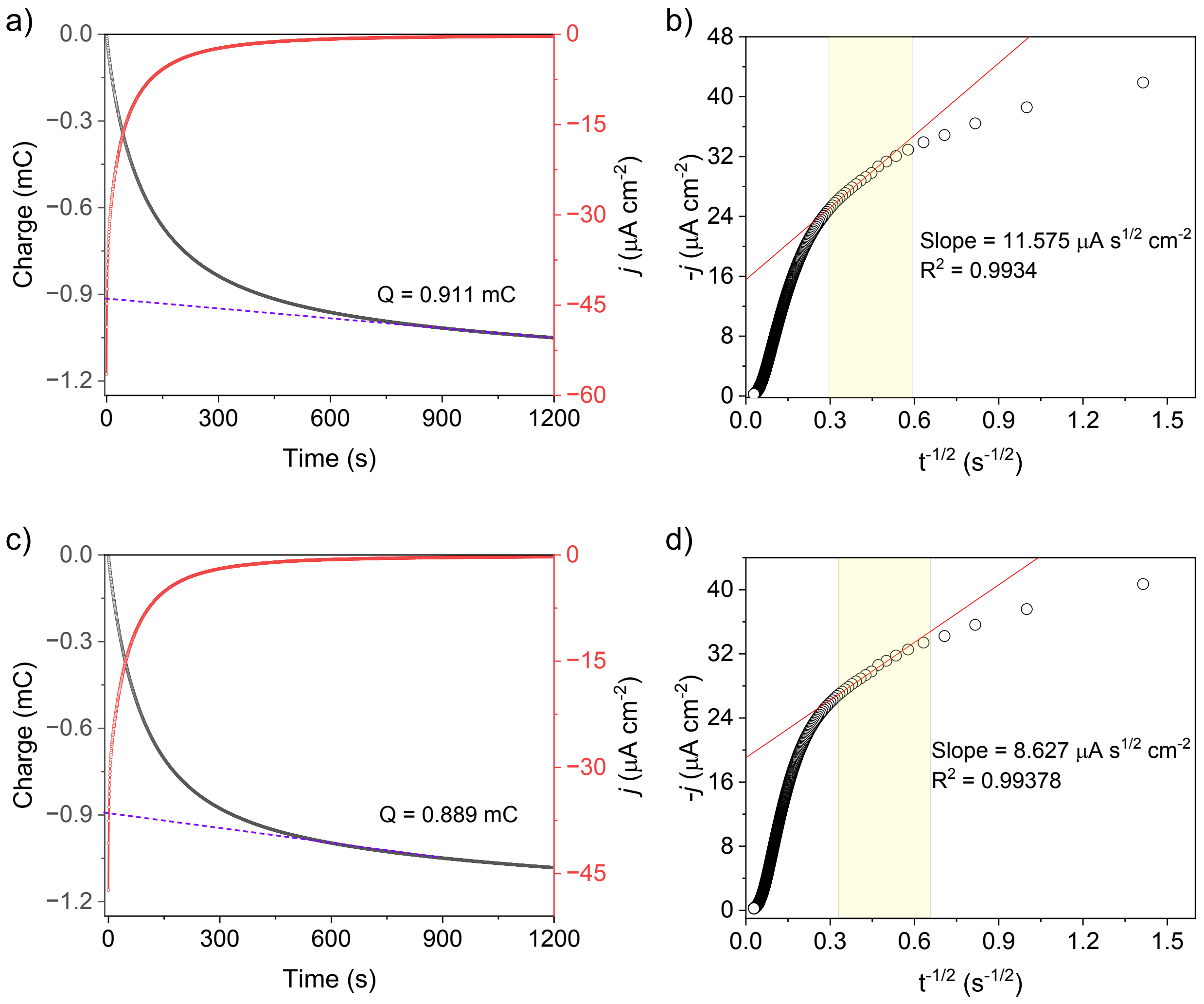

Figure S44. Representative electrochemical data for TAPT-NDI COF@FTO samples measured in 0.1 M KCl in water. (a, c) Chronoamperometry (solid red line) and chronocoulometry (solid black line) after stepping the potential −0.2 V → −0.45 V vs Ag/AgCl (selected from the CV) to isolate the NDI$^{0/\bullet-}$ redox couple. The total charge passed after complete reduction was estimated by subtracting a residual background current (blue dashed line). (b, d) Corresponding Cottrell plots of COF film samples after potential step. Yellow boxes show the data range selected for linear fits.

Table S12. Selected parameters and experimentally measured Γ_e and D_e^{app} for KCl in water

Electrode	S_A (cm^{-2})	d_f (cm)	Q (C)	Cottrell slope	Γ_e (mol cm^{-2})	D_e^{app} (cm^2 s^{-1})
1	0.462	1.216E-4	9.11E-4	1.1575E-5	2.04369E-8	1.59987E-12
2	0.464	1.129E-4	8.89E-4	8.627E-6	1.98574E-8	8.11465E-13
3	0.523	9.37E-5	0.00146	1.836E-5	1.98447E-8	2.53479E-12
4	0.416	9.56E-5	8.207E-4	1.294E-5	2.0447E-8	1.23462E-12

Apparent diffusion coefficient from spectroelectrochemistry

To further verify the D_e^{app} values calculated from chronoamperometry measurements, stepping potential was used in spectroelectrochemistry experiments. In a typical experiment, stepping the potential from non-faradaic region to a suitable potential above the $NDI^{0/\bullet-}$ redox couple, the absorption changes at characteristic wavelength of $NDI^{\bullet-}$ (610 nm or 492 nm) nm was monitored.

The observed change in the absorption during spectroelectrochemistry measurement was used in a modified version of the Cottrell equation to calculate the D_e^{app} values. The linear regions of the change in absorption (ΔA) versus the square root of the time ($t^{1/2}$) plot were used for the estimation of the D_e^{app} values using the following equation:

$$\Delta A = \frac{2A_{max}}{d_f}\sqrt{\frac{D_e^{app}t}{\pi}} \qquad \text{(eq S4)}$$

where ΔA is the change in absorbance, A_{max} is the absorbance maximum, t is time in seconds and d_f is the thickness of the film.

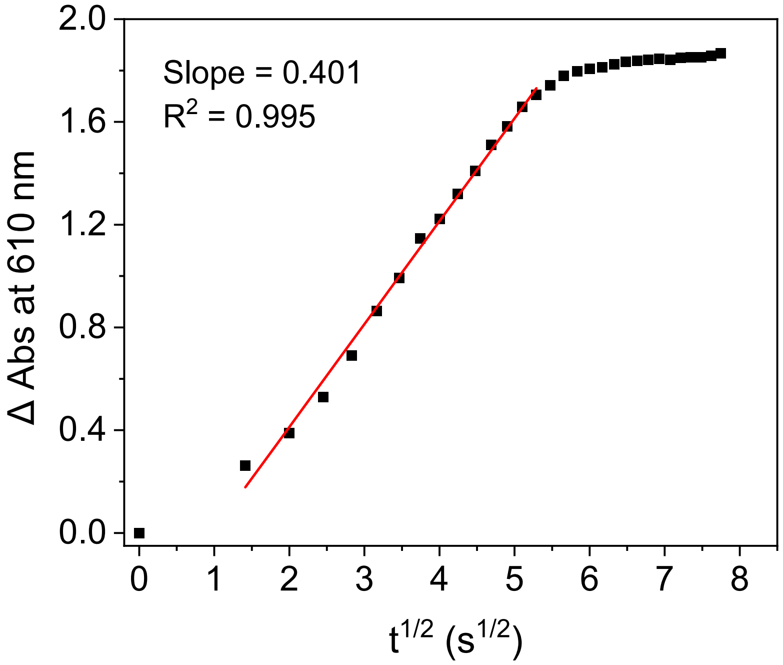

Figure S45. Plot of *ΔA* vs $t^{1/2}$ at a characteristic wavelength of $NDI^{\bullet-}$ (610 nm) in MeCN with $LiClO_4$ as supporting electrolyte.

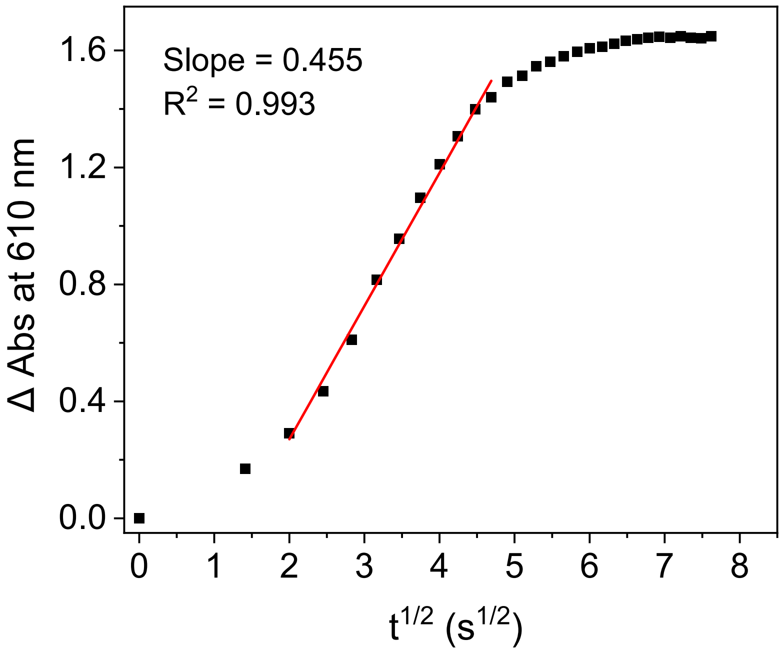

Figure S46. Plot of *ΔA* vs $t^{1/2}$ at a characteristic wavelength of $NDI^{\bullet-}$ (610 nm) in MeCN with $NaClO_4$ as supporting electrolyte.

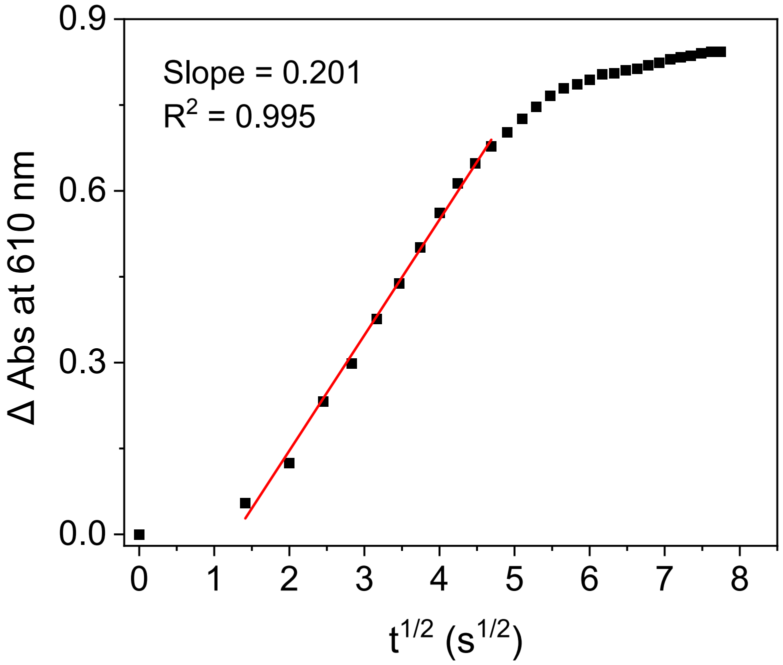

Figure S47. Plot of *ΔA* vs $t^{1/2}$ at a characteristic wavelength of $NDI^{\bullet-}$ (610 nm) in DMF with $LiClO_4$ as supporting electrolyte.

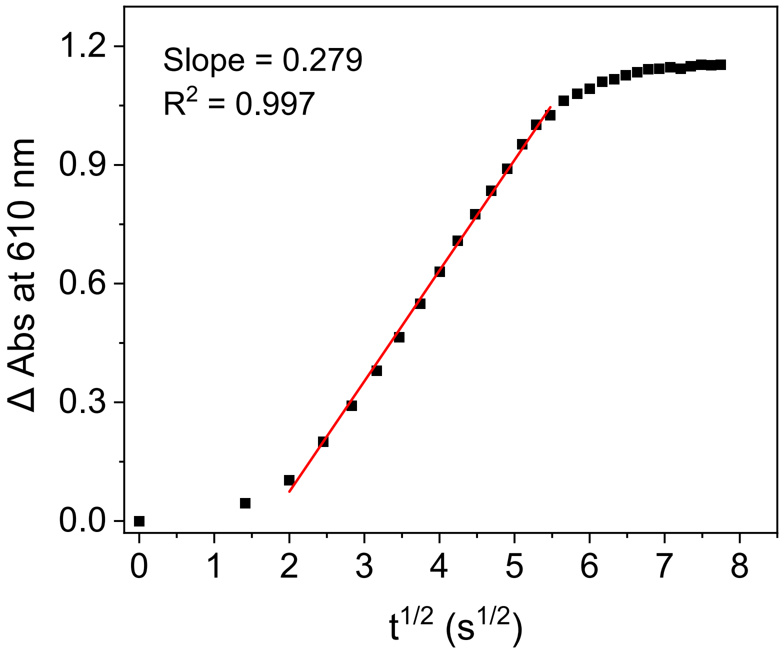

Figure S48. Plot of *ΔA* vs $t^{1/2}$ at a characteristic wavelength of $NDI^{\bullet-}$ (610 nm) in DMF with $NaClO_4$ as supporting electrolyte.

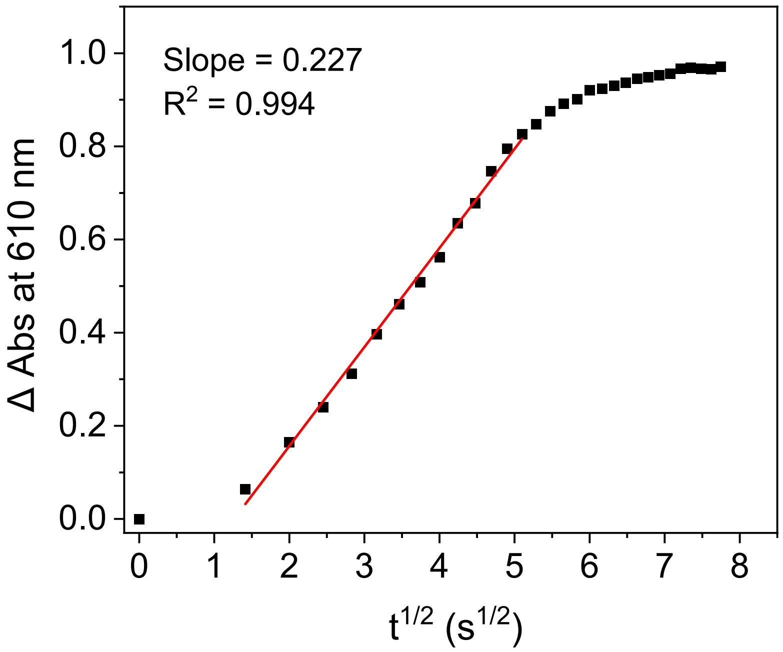

Figure S49. Plot of *ΔA* vs $t^{1/2}$ at a characteristic wavelength of $NDI^{\bullet-}$ (610 nm) in DMF with $KClO_4$ as supporting electrolyte.

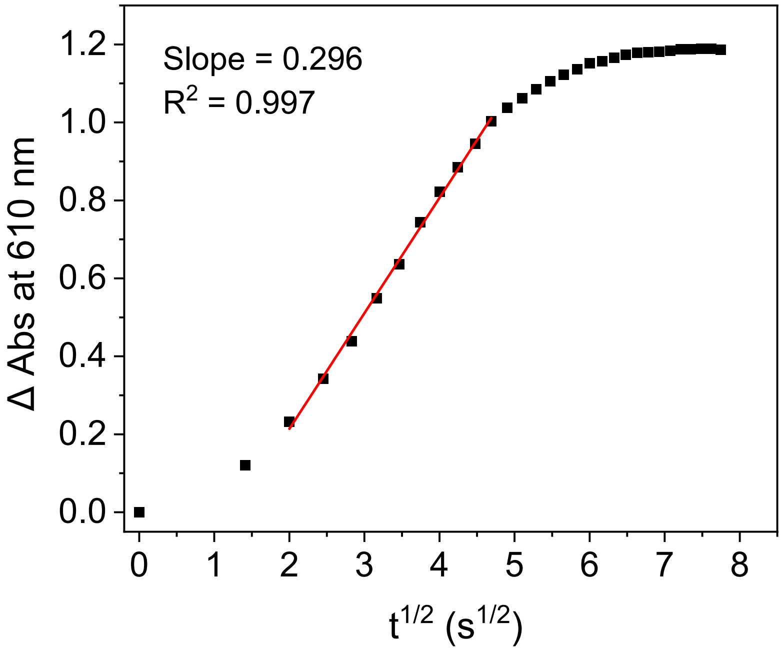

Figure S50. Plot of *ΔA* vs $t^{1/2}$ at a characteristic wavelength of $NDI^{\bullet-}$ (610 nm) in EtOH with $LiClO_4$ as supporting electrolyte.

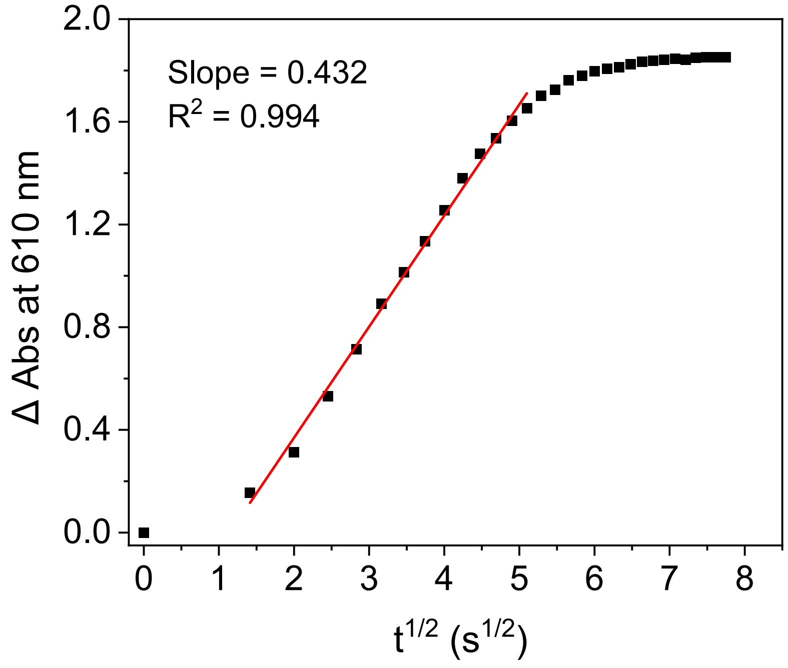

Figure S51. Plot of *ΔA* vs $t^{1/2}$ at a characteristic wavelength of $NDI^{•-}$ (610 nm) in EtOH with $NaClO_4$ as supporting electrolyte.

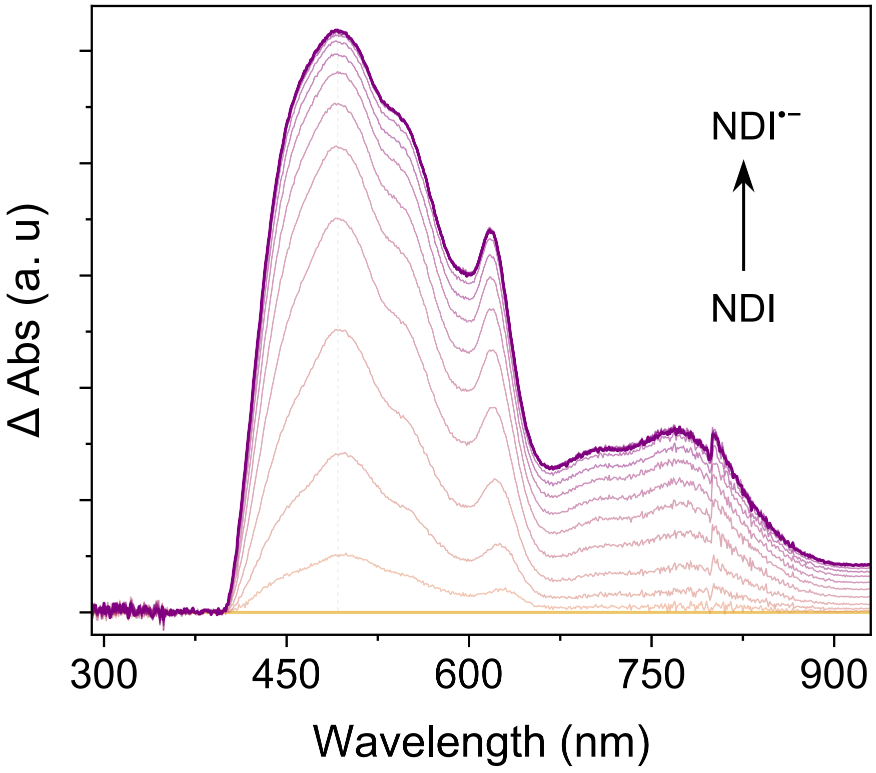

Figure S52. Change in absorbance of NDI$^{•-}$ in a step-potential chronoamperometry measurement in 0.1 M KCl aqueous electrolyte. The maximum change in absorbance occurs at 492 nm, which was used in the kinetic experiments.

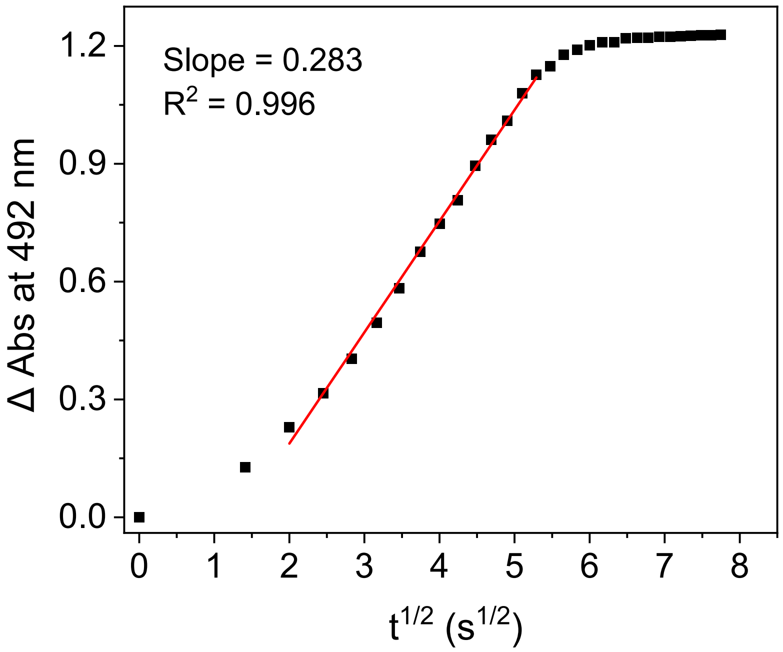

Figure S53. Plot of *ΔA* vs $t^{1/2}$ at a characteristic wavelength of $NDI^{\bullet-}$ (492 nm) in water with LiCl as supporting electrolyte.

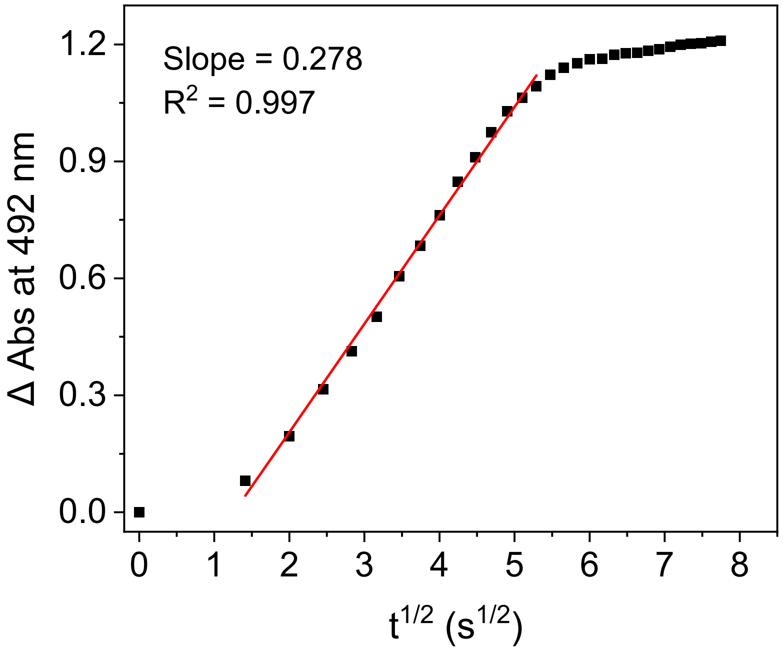

Figure S54. Plot of *ΔA* vs $t^{1/2}$ at a characteristic wavelength of $NDI^{\bullet -}$ (492 nm) in water with NaCl as supporting electrolyte.

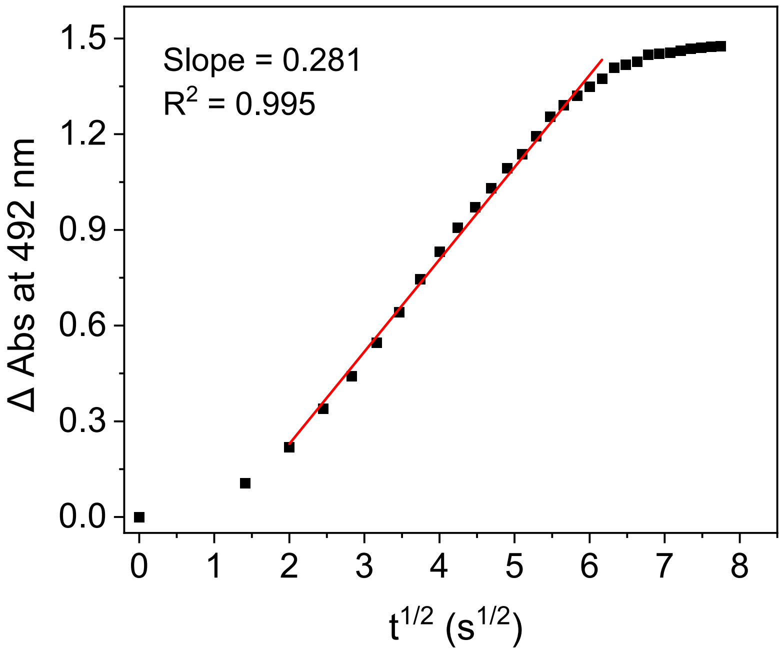

Figure S55. Plot of *ΔA* vs $t^{1/2}$ at a characteristic wavelength of $NDI^{•-}$ (492 nm) in water with KCl as supporting electrolyte.

Table S13. Average D_e^{app} estimated for the $NDI^{0/\bullet-}$ redox couple for TAPT-NDI@FTO COF films

	D_e^{app} from Chronoamperometry ($cm^2\ s^{-1}$)			D_e^{app} from Spectroeletrochemistry ($cm^2\ s^{-1}$)		
**Solvent**	**Li^+**	**Na^+**	**K^+**	**Li^+**	**Na^+**	**K^+**
MeCN	2.36 ± 1.06 E-11	4.29 ± 2.16 E-11	7.93 ± 3.39 E-11	2.53 ± 1.64 E-11	4.12 ± 2.00 E-11	5.63 ± 2.61 E-11
DMF	4.81 ± 2.89 E-12	7.65 ± 3.65 E-12	8.17 ± 5.49 E-12	5.18 ± 2.71 E-12	9.08 ± 5.97 E-12	1.13 ± 0.76 E-11
EtOH	3.27 ± 2.34 E-12	5.19 ± 1.66 E-12		4.28 ± 3.02 E-12	8.06 ± 5.97 E-12	
Water	4.94 ± 2.74 E-13	1.05 ± 0.57 E-12	1.55 ± 0.73 E-12	2.11 ± 1.08 E-12	2.96 ± 2.01 E-12	3.75 ± 2.14 E-12

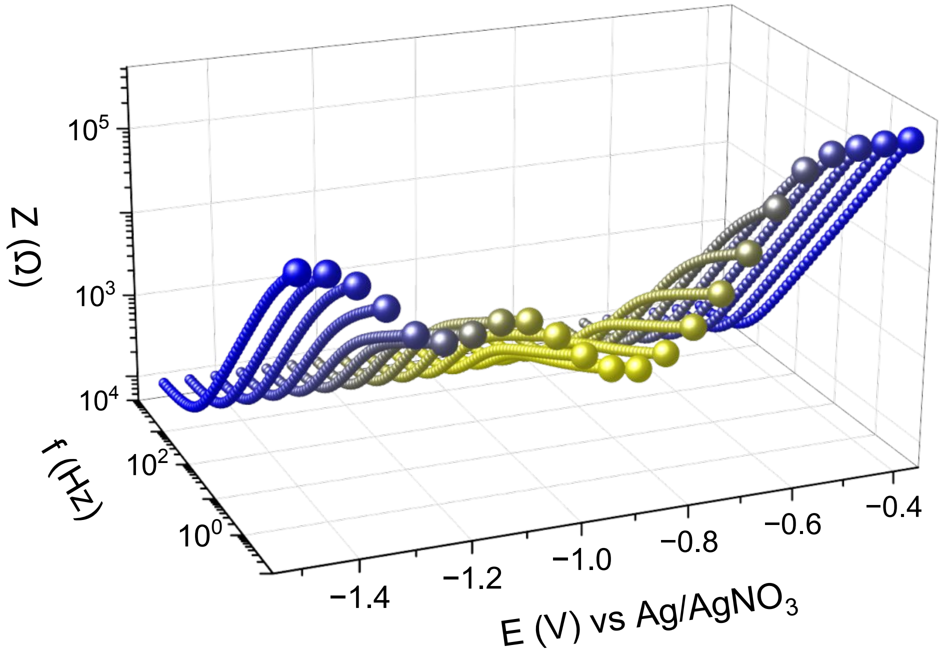

Figure S56. Bode plots of TAPT-NDI COF film at different applied potentials vs Ag/$AgNO_3$ measured in 0.1 M $LiClO_4$ in DMF. The impedance data point for each measurement is magnified at the frequency of 0.1 Hz for better visualization at different redox states. Each impedance measurement is preceded by a stabilization period of 120 s at the respective potentials, ensuring the redox state is achieved.

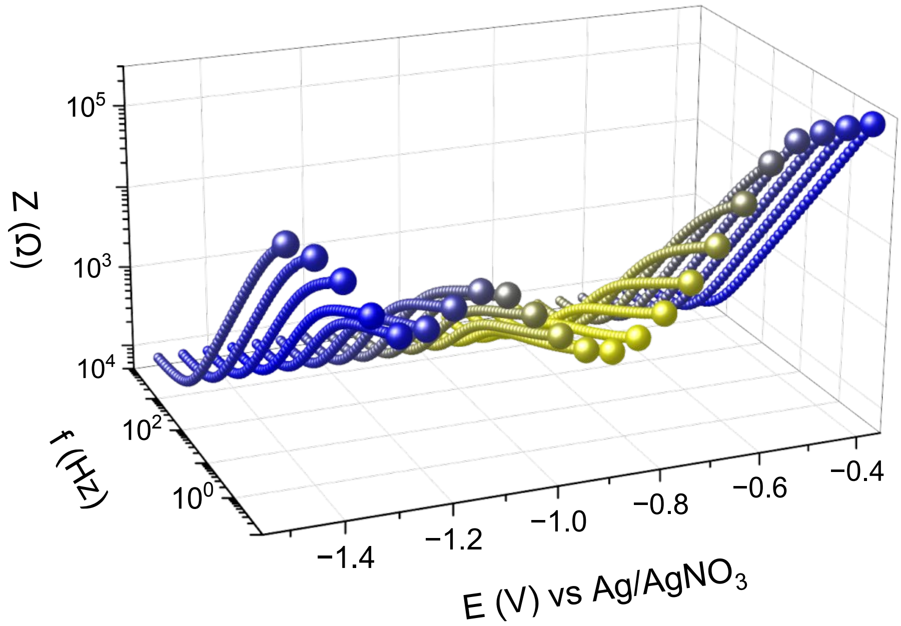

Figure S57. Bode plots of TAPT-NDI COF film at different applied potentials vs Ag/AgNO$_3$ measured in 0.1 M NaClO$_4$ in DMF. The impedance data point for each measurement is magnified at the frequency of 0.1 Hz for better visualization at different redox states. Each impedance measurement is preceded by a stabilization period of 120 s at the respective potentials, ensuring the redox state is achieved.

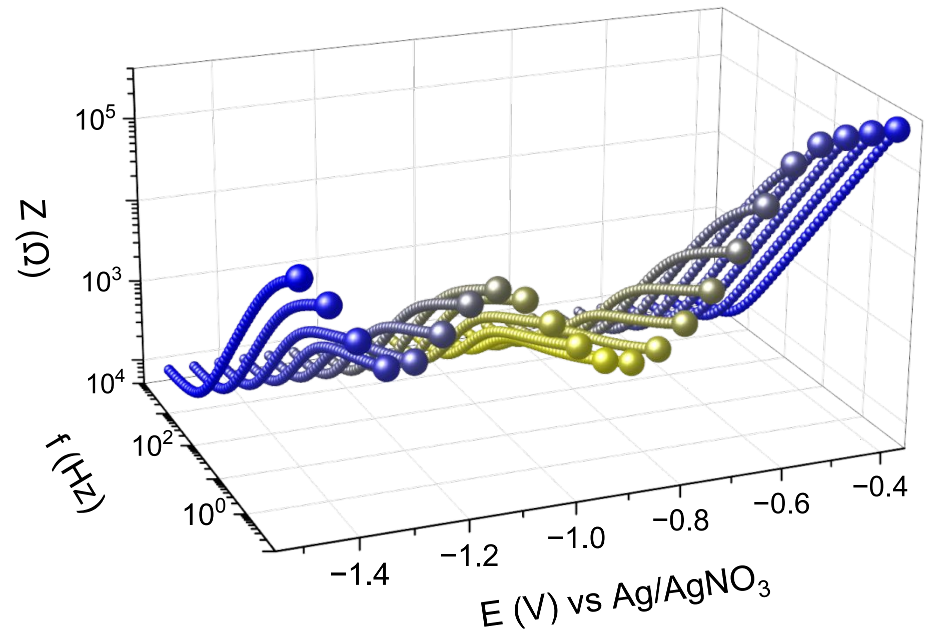

Figure S58. Bode plots of TAPT-NDI COF film at different applied potentials vs Ag/AgNO$_3$ measured in 0.1 M KClO$_4$ in DMF. The impedance data point for each measurement is magnified at the frequency of 0.1 Hz for better visualization at different redox states. Each impedance measurement is preceded by a stabilization period of 120 s at the respective potentials, ensuring the redox state is achieved.

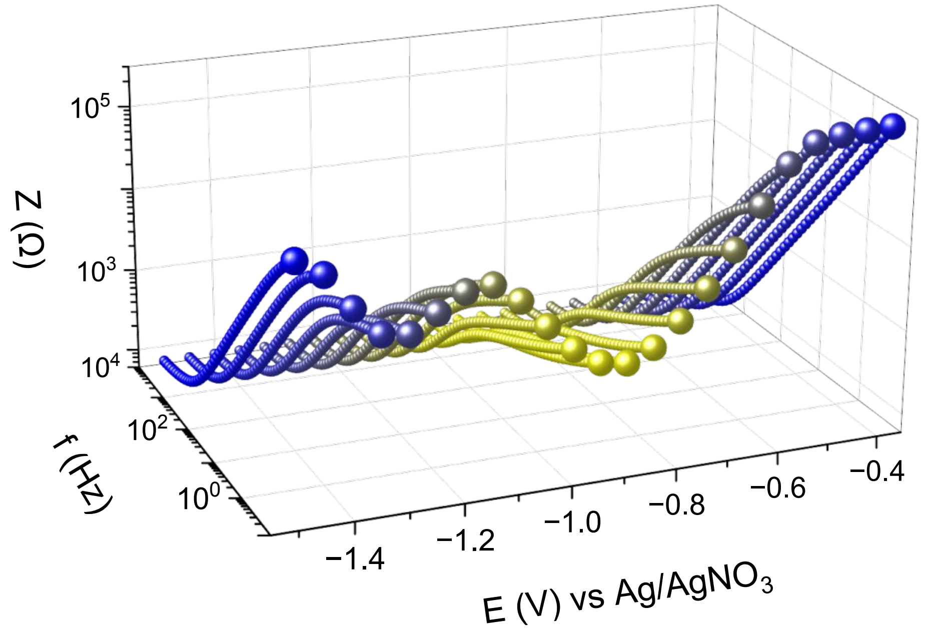

Figure S59. Bode plots of TAPT-NDI COF film at different applied potentials vs Ag/AgNO$_3$ measured in 0.1 M KPF$_6$ in DMF. The impedance data point for each measurement is magnified at the frequency of 0.1 Hz for better visualization at different redox states. Each impedance measurement is preceded by a stabilization period of 120 s at the respective potentials, ensuring the redox state is achieved.

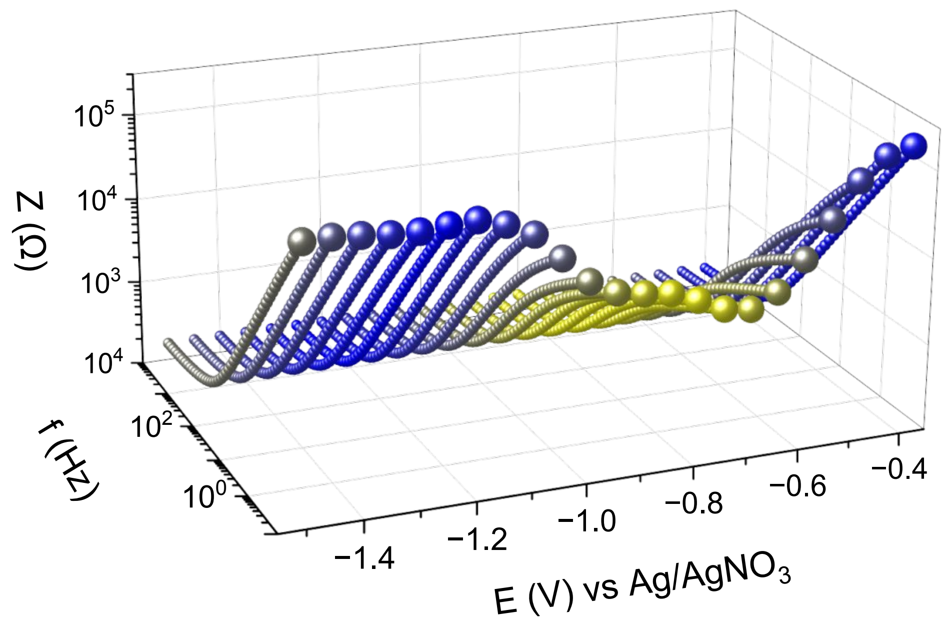

Figure S60. Bode plots of TAPT-NDI COF film at different applied potentials vs Ag/$AgNO_3$ measured in 0.1 M $LiClO_4$ in ethanol. The impedance data point for each measurement is magnified at the frequency of 0.1 Hz for better visualization at different redox states. Each impedance measurement is preceded by a stabilization period of 120 s at the respective potentials, ensuring the redox state is achieved.

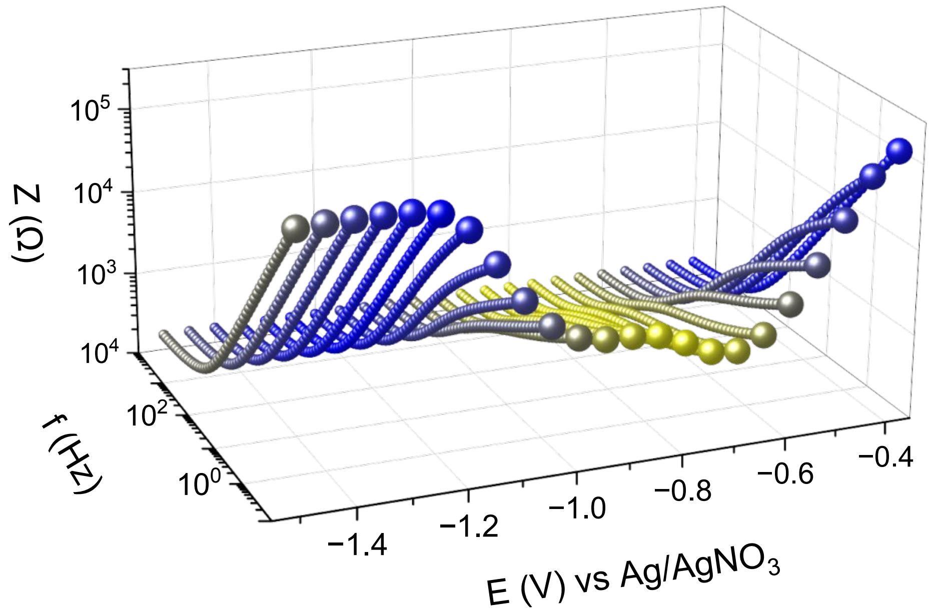

Figure S61. Bode plots of TAPT-NDI COF film at different applied potentials vs Ag/AgNO$_3$ measured in 0.1 M NaClO$_4$ in ethanol. The impedance data point for each measurement is magnified at the frequency of 0.1 Hz for better visualization at different redox states. Each impedance measurement is preceded by a stabilization period of 120 s at the respective potentials, ensuring the redox state is achieved.

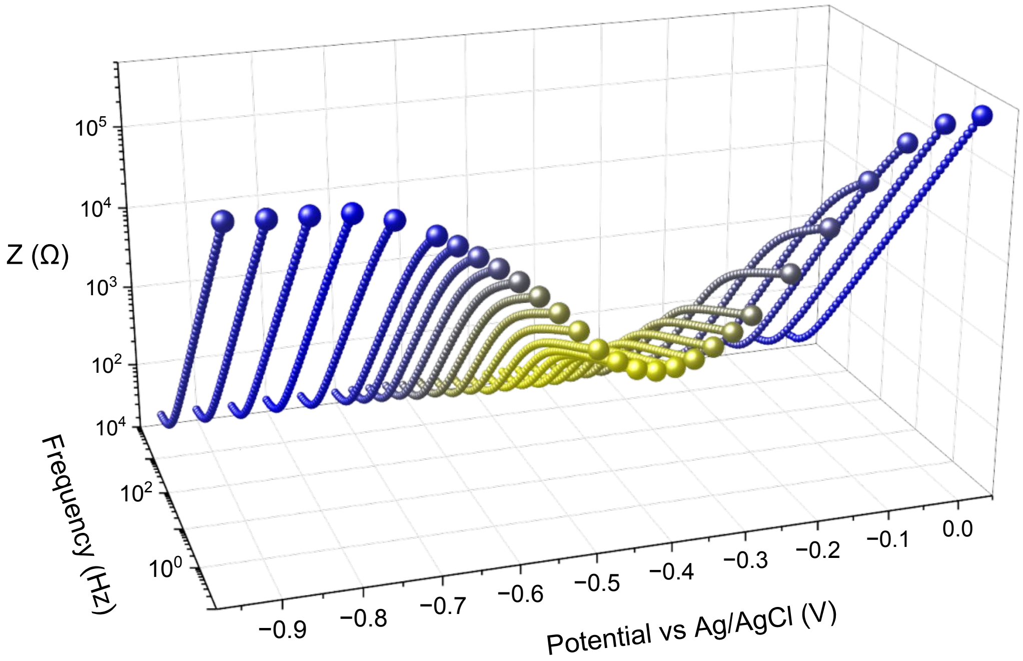

Figure S62. Bode plots of TAPT-NDI COF film at different applied potentials vs Ag/AgCl measured in 0.1 M LiCl aqueous electrolyte. The impedance data point for each measurement is magnified at the frequency of 0.1 Hz for better visualization at different redox states. Each impedance measurement is preceded by a stabilization period of 120 s at the respective potentials, ensuring the redox state is achieved.

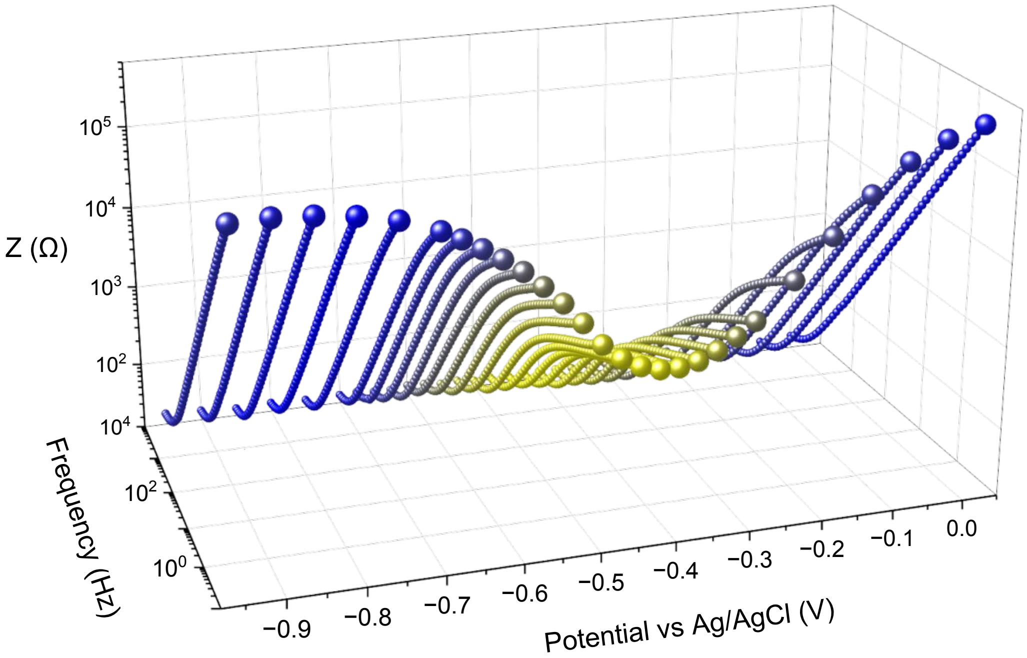

Figure S63. Bode plots of TAPT-NDI COF film at different applied potentials vs Ag/AgCl measured in 0.1 M NaCl aqueous electrolyte. The impedance data point for each measurement is magnified at the frequency of 0.1 Hz for better visualization at different redox states. Each impedance measurement is preceded by a stabilization period of 120 s at the respective potentials, ensuring the redox state is achieved.

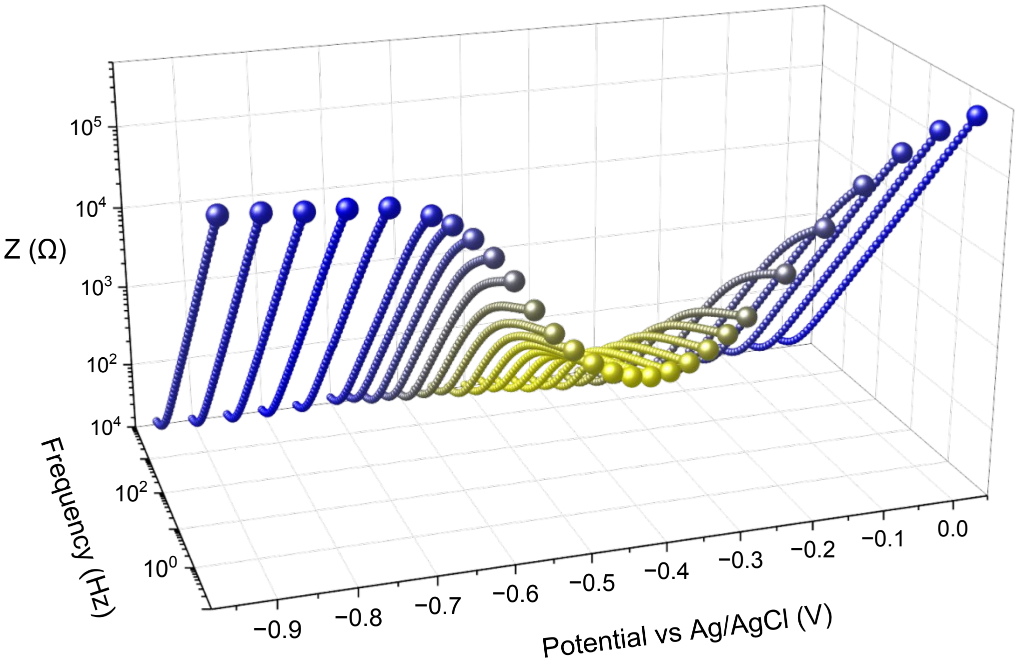

Figure S64. Bode plots of TAPT-NDI COF film at different applied potentials vs Ag/AgCl measured in 0.1 M KCl aqueous electrolyte. The impedance data point for each measurement is magnified at the frequency of 0.1 Hz for better visualization at different redox states. Each impedance measurement is preceded by a stabilization period of 120 s at the respective potentials, ensuring the redox state is achieved.

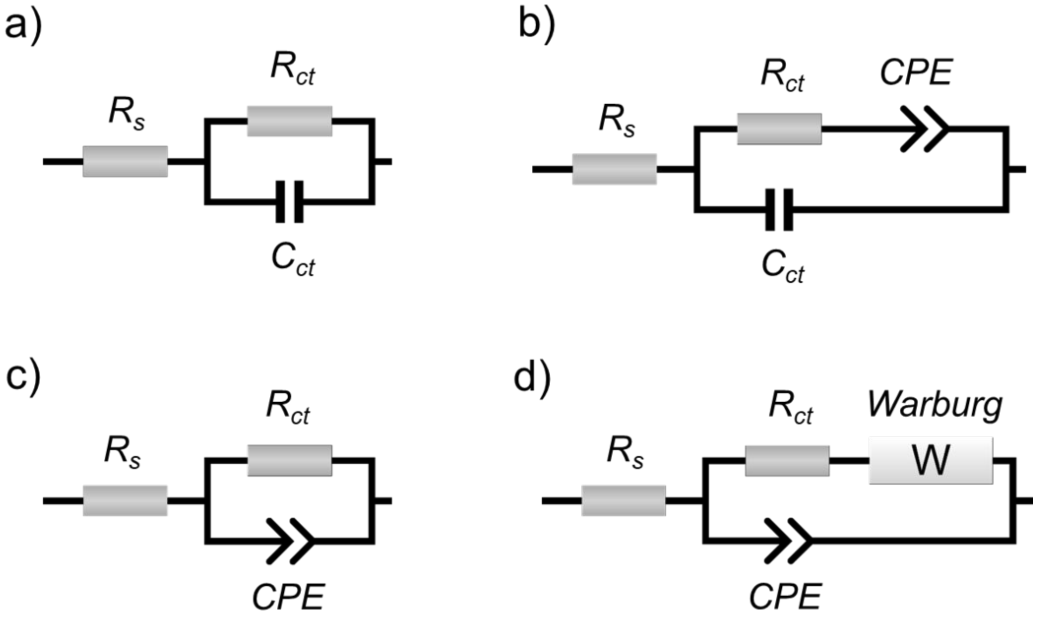

Figure S65. Simulated equivalent circuits (EC) used for fitting the experimental impedance data. (a) Simplified RC circuit, where R_{ct} and C_{ct} stand for resistance and capacitance related to the inter-site cation coupled electron hopping. (b) Modified RC circuit by adding a constant phase element (CPE) in series to the electronic resistance component, where CPE is primarily defined by its phase, n ($0 \leq n \leq 1$), when n equals 1, 0.5, or 0, the CPE represent an ideal capacitor, a semi-infinite diffusional Warburg element, and an ideal resistor, respectively. (c) Modified RC circuit by replacing C_{ct} with a CPE. (d) Modified RC circuit by adding the serial Warburg element to the electronic resistance component. In all circuits, R_s stands for the resistance related to the electrolyte.

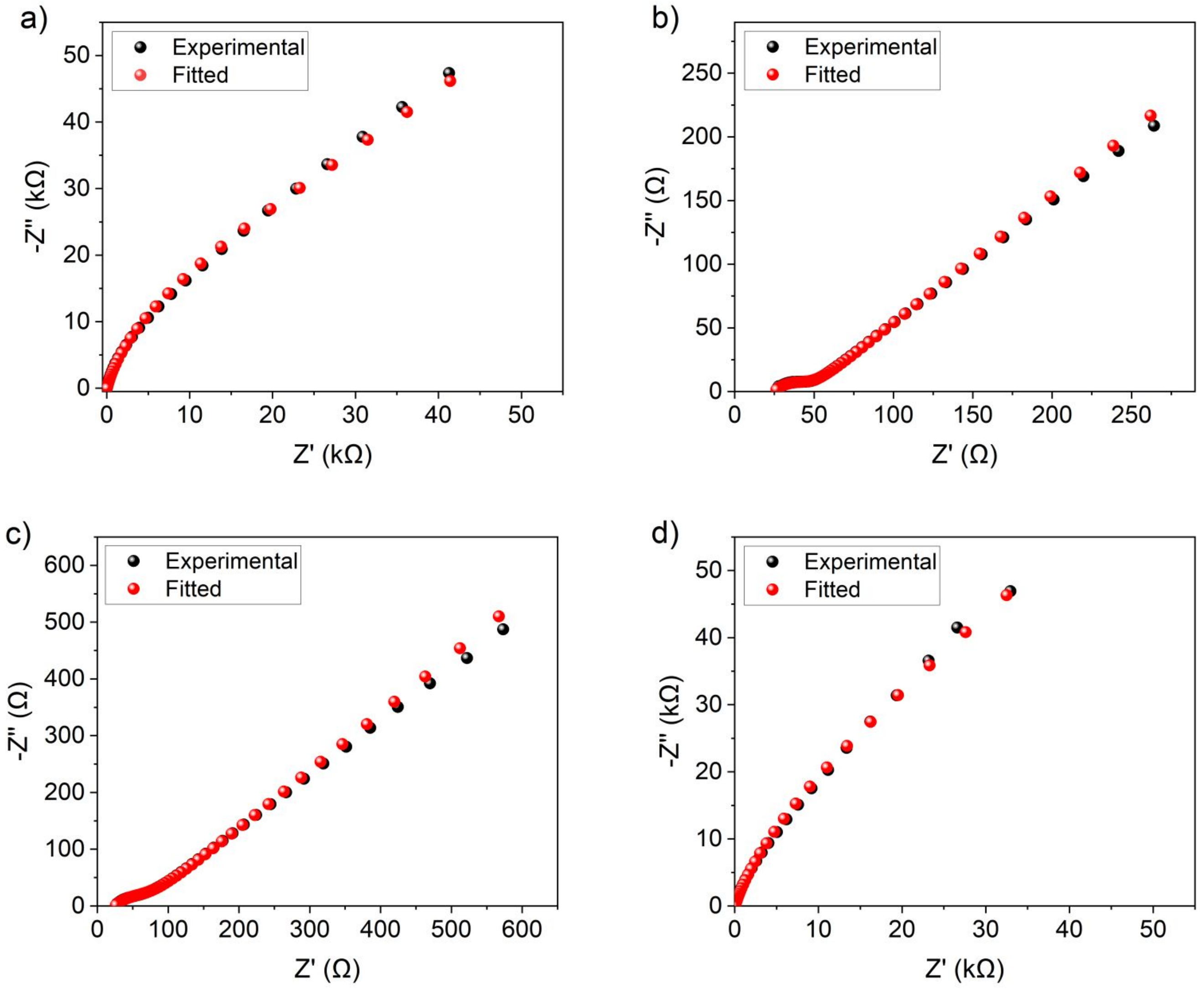

Figure S66. Representative Nyquist plots of experimental and fitted (circuit in Figure S65b) electrochemical impedance data for (a) 0.0-(TAPT-NDI), (b) 0.5-(TAPT-NDI), (c) 1.5-(TAPT-NDI), and (d) 2.0-(TAPT-NDI) films measured in 0.1 M KPF_6 MeCN electrolyte.

Table S14. Fitting results of the EIS measurement for Figure S66a 0.0-(TAPT-NDI)

Parameters	Value	Fit Error	Fit Error (%)
R_S (Ω)	41.6	3.2	7.8
R_{ct} (Ω)	3643.02	376.29	10
CPE Q 1	1.766E-05	6.361E-08	0.36
CPE Alpha 1	0.46	0.0018	0.39
C_{ct} (F)	5.18E-06	4.77E-08	0.92

Table S15. Fitting results of the EIS measurement for Figure S66b 0.5-(TAPT-NDI)

Parameters	Value	Fit Error	Fit Error (%)
R_S (Ω)	27.9	0.23	0.83
R_{ct} (Ω)	15.7	0.43	2.7
CPE Q 1	0.00337	1.7E-05	0.51
CPE Alpha 1	0.478	0.0027	0.58
C_{ct} (F)	3.67E-06	1.8E-07	5

Table S16. Fitting results of the EIS measurement for Figure S66c 1.5-(TAPT-NDI)

Parameters	Value	Fit Error	Fit Error (%)
R_S (Ω)	28.4	0.3	1.1
R_{ct} (Ω)	24.07	0.73	3
CPE Q 1	0.00162	5.7E-06	0.35
CPE Alpha 1	0.483	0.002	0.41
C_{ct} (F)	4.28E-06	1.95E-07	4.6

Table S17. Fitting results of the EIS measurement for Figure S66d 2.0-(TAPT-NDI)

Parameters	Value	Fit Error	Fit Error (%)
R_S (Ω)	19.2	8.5	44
R_{ct} (Ω)	3.54E+06	9.24E+04	2.6
CPE Q 1	6.78E-06	4.5E-09	0.066
CPE Alpha 1	0.929	0.00067	0.072

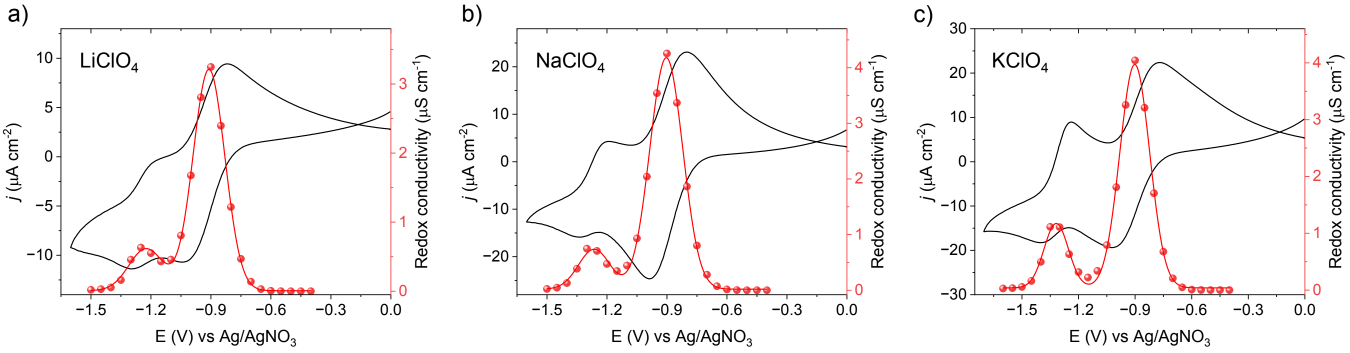

Figure S67. Evolution of the steady-state thin-film conductivity in (a) Li^{+}, (b) Na^{+}, (c) K^{+} electrolyte in DMF as a function of applied electrochemical potential, which determines the mole fraction of electron reduction, x-TAPT-NDI, $0.0 \leq x \leq 2.0$. Gaussian fit was performed for both NDI/$NDI^{\bullet-}$ and $NDI^{\bullet-}$/NDI^{2-} bell-shaped redox conductivity.

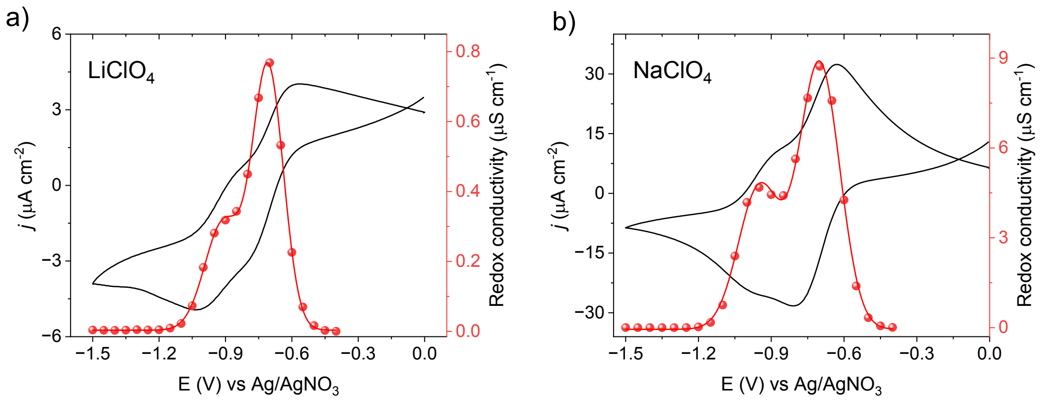

Figure S68. Evolution of the steady-state thin-film conductivity in (a) Li^+, (b) Na^+ electrolyte in EtOH as a function of applied electrochemical potential, which determines the mole fraction of electron reduction, *x*-TAPT-NDI, $0.0 \leq x \leq 2.0$. Gaussian fit was performed for both NDI/$NDI^{\bullet-}$ and $NDI^{\bullet-}$/NDI^{2-} bell-shaped redox conductivity.

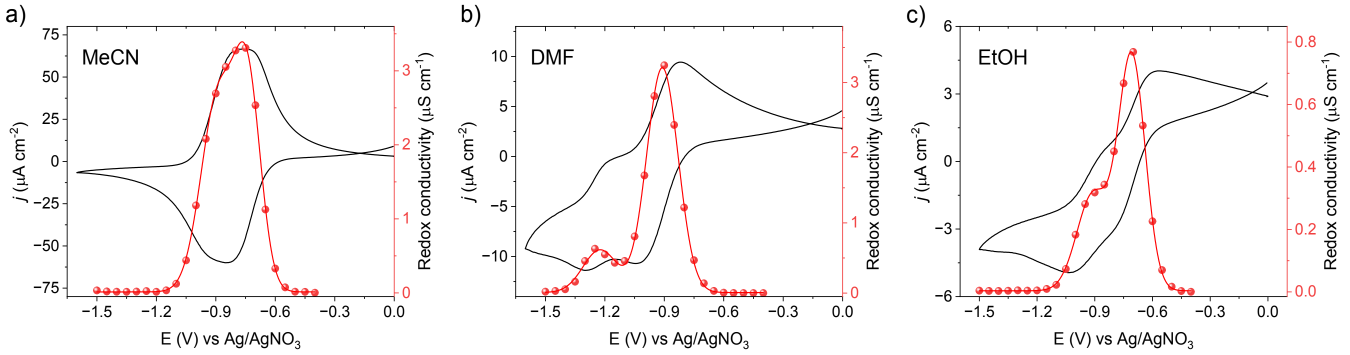

Figure S69. Evolution of the steady-state thin-film conductivity in Li^+ electrolyte (a) MeCN, (b) DMF, (c) EtOH as a function of applied electrochemical potential, which determines the mole fraction of electron reduction, *x*-TAPT-NDI, $0.0 \leq x \leq 2.0$. Gaussian fit was performed for both NDI/$NDI^{\bullet-}$ and $NDI^{\bullet-}$/NDI^{2-} bell-shaped redox conductivity.

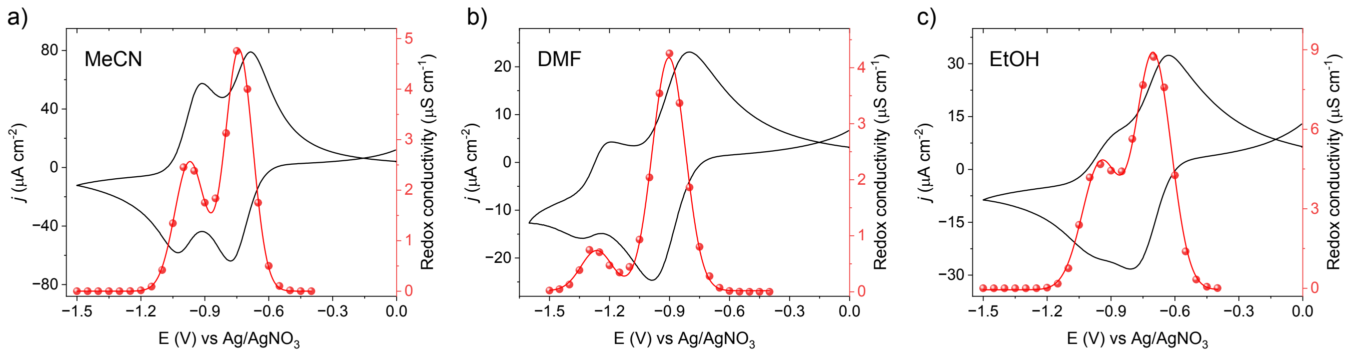

Figure S70. Evolution of the steady-state thin-film conductivity in Na^+ electrolyte (a) MeCN, (b) DMF, (c) EtOH as a function of applied electrochemical potential, which determines the mole fraction of electron reduction, *x*-TAPT-NDI, $0.0 \leq x \leq 2.0$. A Gaussian fit was performed for both NDI/NDI$^{\bullet-}$ and NDI$^{\bullet-}$/NDI^{2-} bell-shaped redox conductivity.

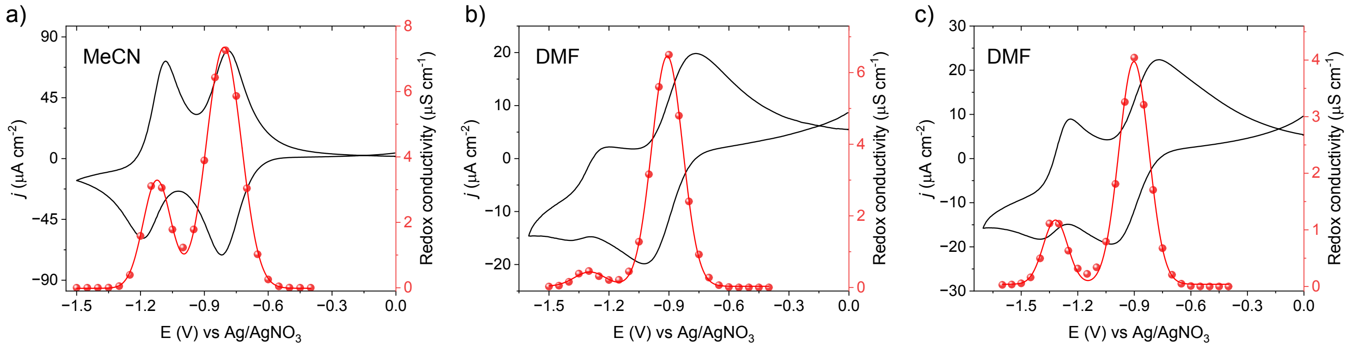

Figure S71. Evolution of the steady-state thin-film conductivity (a) KPF_6 in MeCN, (b) KPF_6 in DMF, (c) $KClO_4$ in DMF as a function of applied electrochemical potential, which determines the mole fraction of electron reduction, x-TAPT-NDI, $0.0 \leq x \leq 2.0$. A Gaussian fit was performed for both NDI/NDI$^{\bullet-}$ and NDI$^{\bullet-}$/NDI^{2-} bell-shaped redox conductivity.

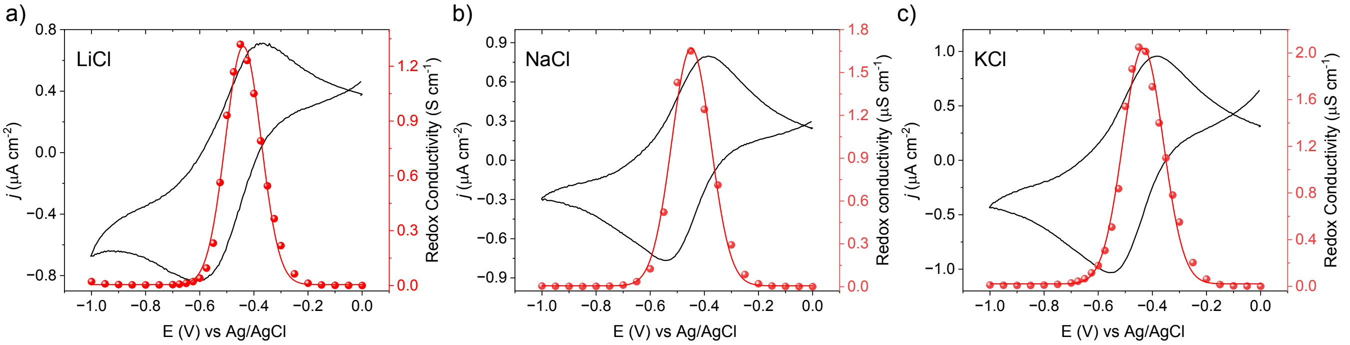

Figure S72. Evolution of the steady-state thin-film conductivity in (a) Li^+, (b) Na^+, (c) K^+ electrolyte in water as a function of applied electrochemical potential, which determines the mole fraction of electron reduction, x-TAPT-NDI, $0.0 \leq x \leq 2.0$. A Gaussian fit was performed for bell-shaped redox conductivity.

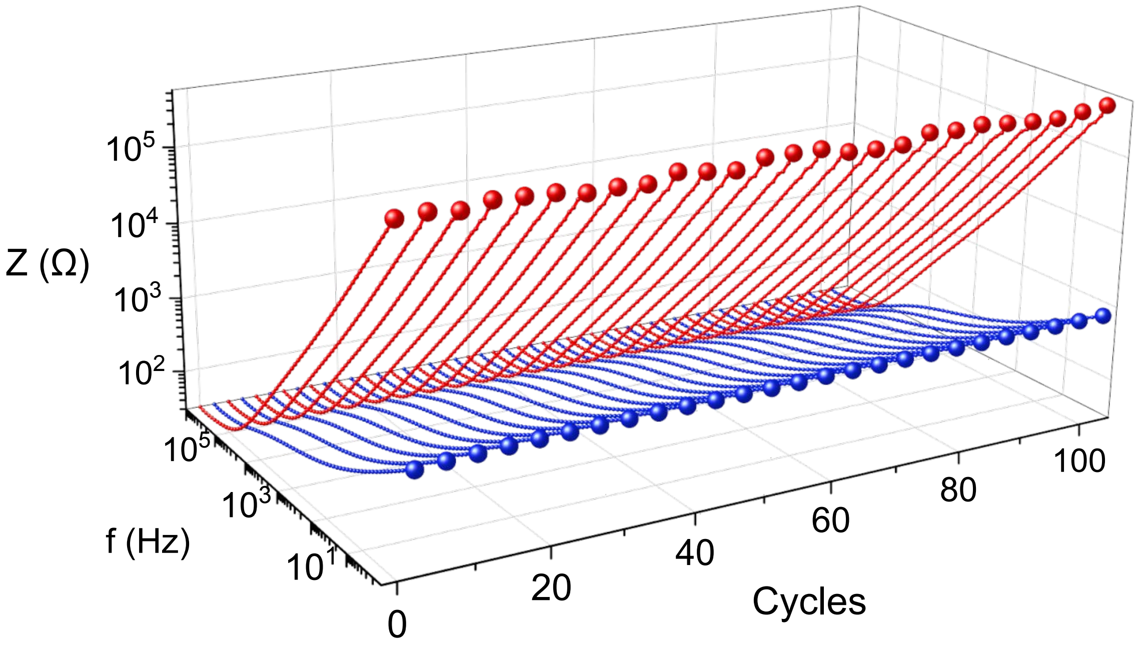

Figure S73. Insulator-semiconductor switching behavior between 0.0-(NDI) (red) and 0.5-(NDI) (blue) for 100 cycles (~20 h operation). Bode plots of TAPT-NDI COF film at 0 V (red) and −0.85 V (blue) vs $Ag/AgNO_3$ measured in 0.1 M KPF_6 in MeCN. The impedance data point for each measurement is magnified at the frequency of 0.1 Hz for better visualization. Each impedance measurement is preceded by a stabilization period of 180 s at 0 V (red) and −0.85 V (blue) vs $Ag/AgNO_3$ ensuring the redox state is achieved.

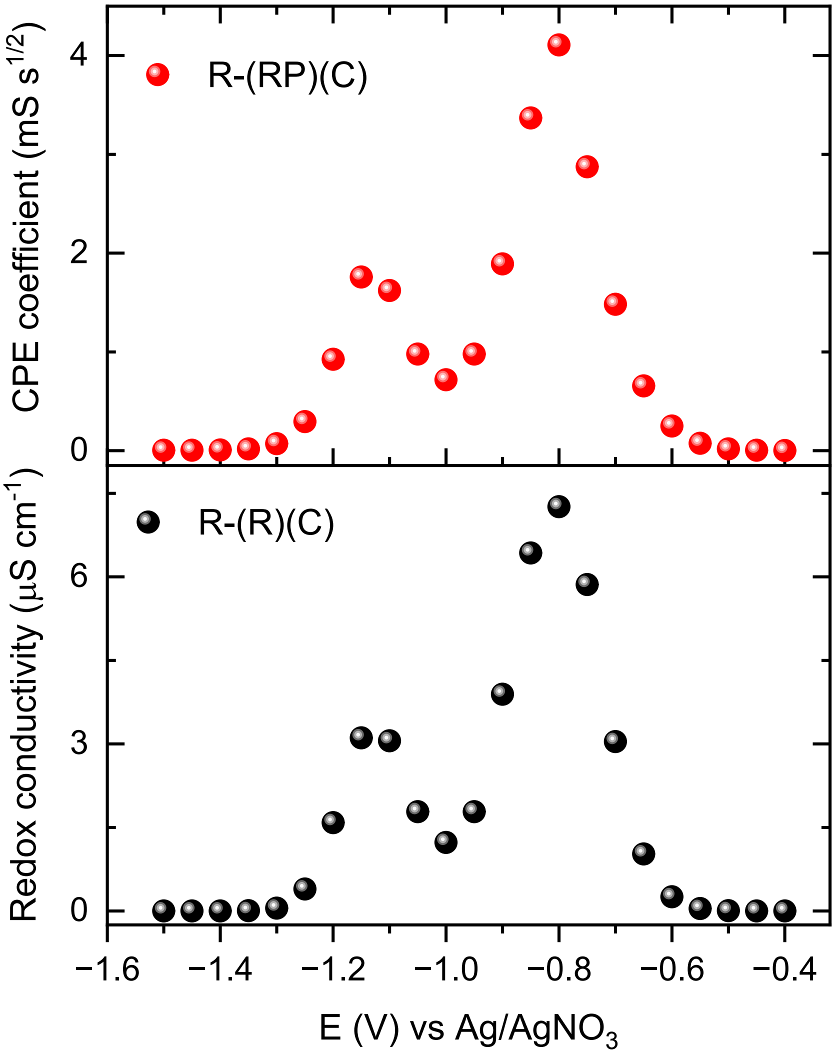

Figure S74. Evolution of redox conductivity (lower panel, simulated from Figure S65a), and CPE coefficient (upper panel, simulated from Figure S65b) as the function of applied electrochemical potential (different redox state of the film) measured in 0.1 M KPF_6 MeCN electrolyte, which is determined by the mole fraction of electron reduction, *x*-(TAPT-NDI), $0.0 \leq x \leq 2.0$. Note that the unit of the Y-axes for upper panel is changing upon the phase, *n*, to facilitate comparison, *n* was assumed to be 0.5 here.

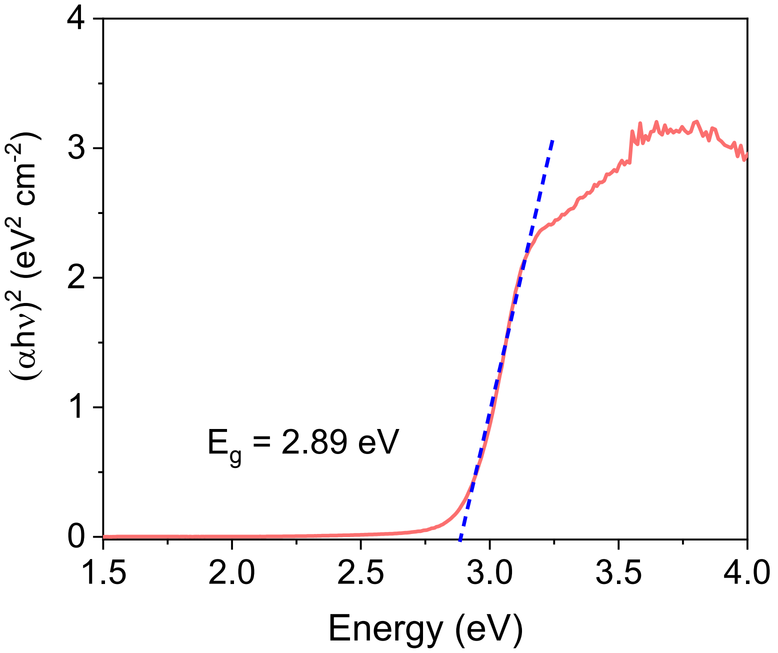

Figure S75. The Tauc plot of TAPT-NDI COF obtained from UV-vis spectrum and extracted optical band gaps.

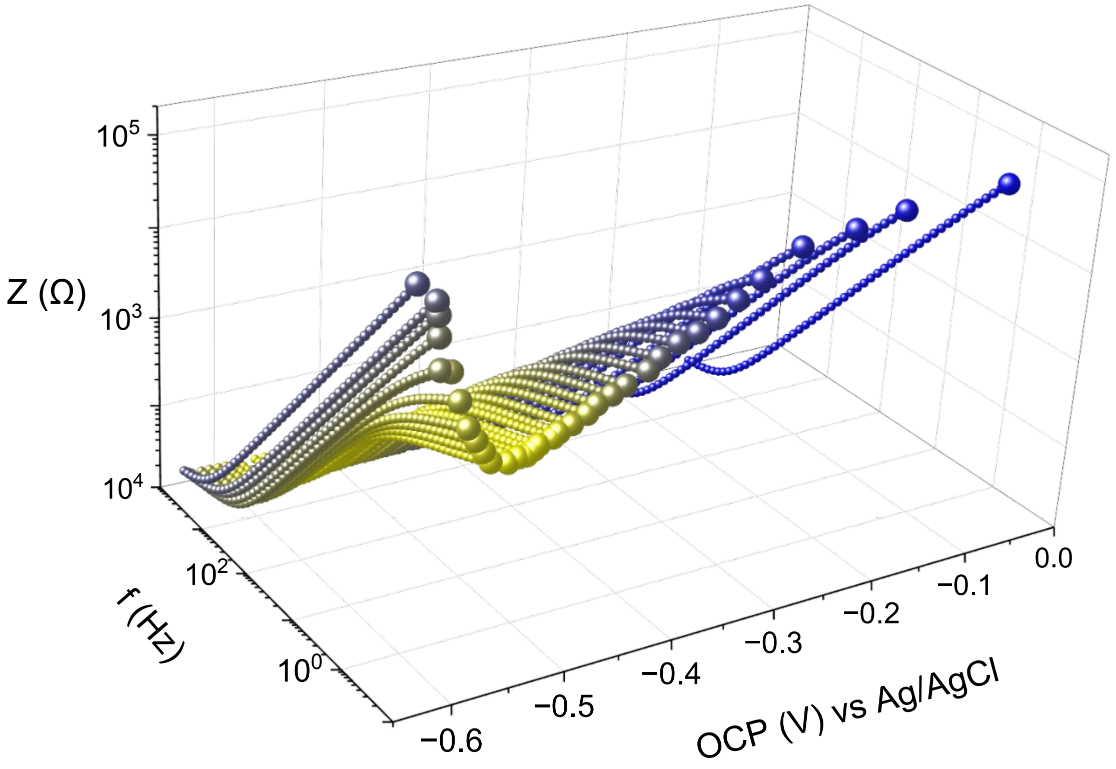

Figure S76. Bode plots of TAPT-NDI COF film at different OCP vs Ag/AgCl induced by 365 nm UV illumination at various time intervals. The measurements were performed in oxygen-free 0.1 M LiCl aqueous electrolyte in the presence of 10 mM sacrificial electron donor 4-MBA. The impedance data point for each measurement is magnified at the frequency of 0.1 Hz for better visualization of different redox states.

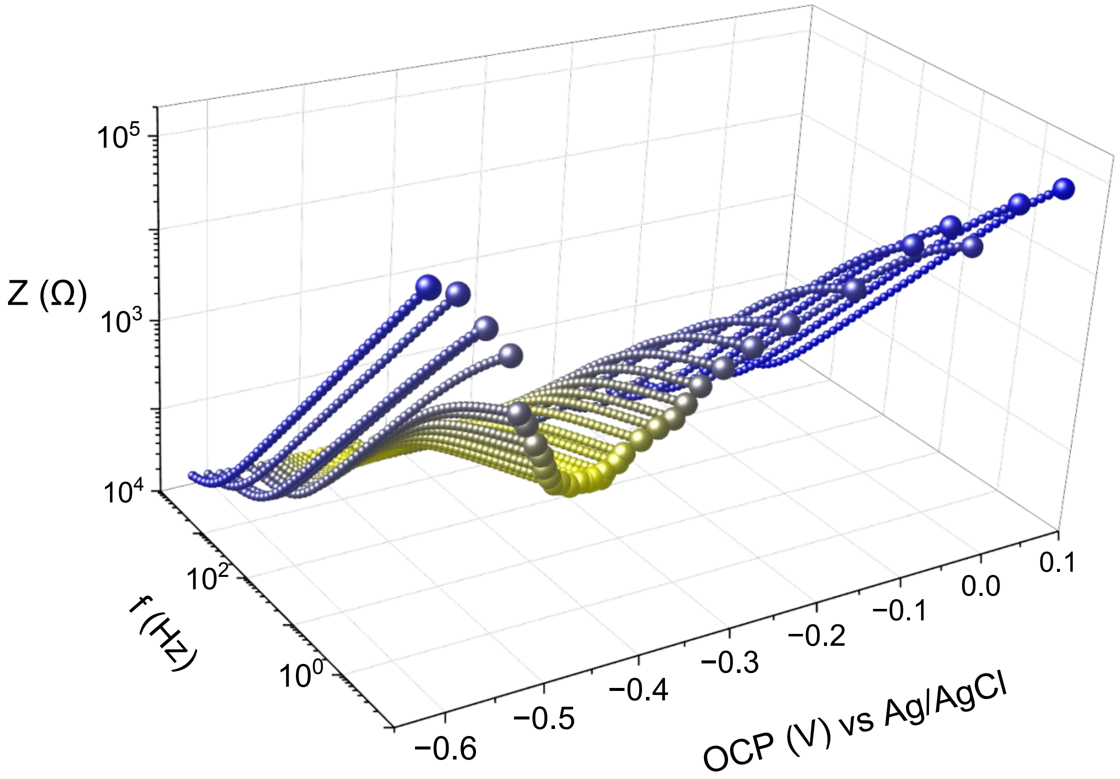

Figure S77. Bode plots of TAPT-NDI COF film at different OCP vs Ag/AgCl induced by 365 nm UV illumination at various time intervals. The measurements were performed in oxygen-free 0.1 M NaCl aqueous electrolyte in the presence of 10 mM sacrificial electron donor 4-MBA. The impedance data point for each measurement is magnified at the frequency of 0.1 Hz for better visualization of different redox states.

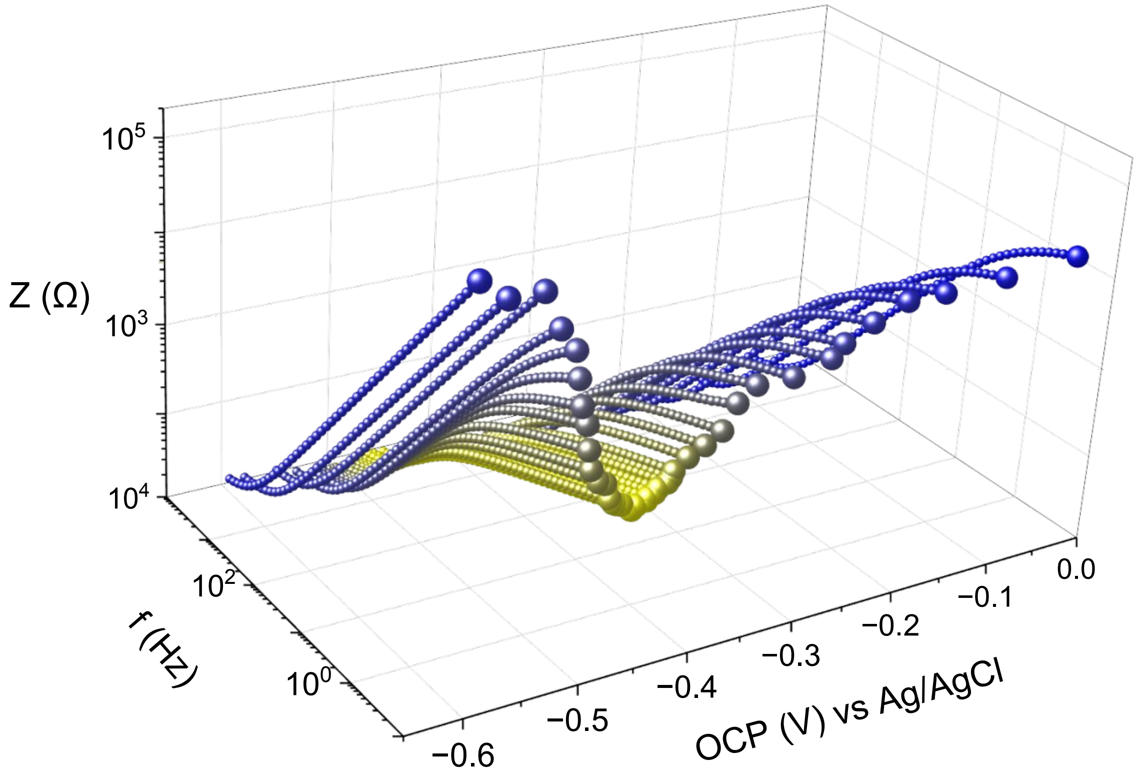

Figure S78. Bode plots of TAPT-NDI COF film at different OCP vs Ag/AgCl induced by 365 nm UV illumination at various time intervals. The measurements were performed in oxygen-free 0.1 M RbCl aqueous electrolyte in the presence of 10 mM sacrificial electron donor 4-MBA. The impedance data point for each measurement is magnified at the frequency of 0.1 Hz for better visualization of different redox states.

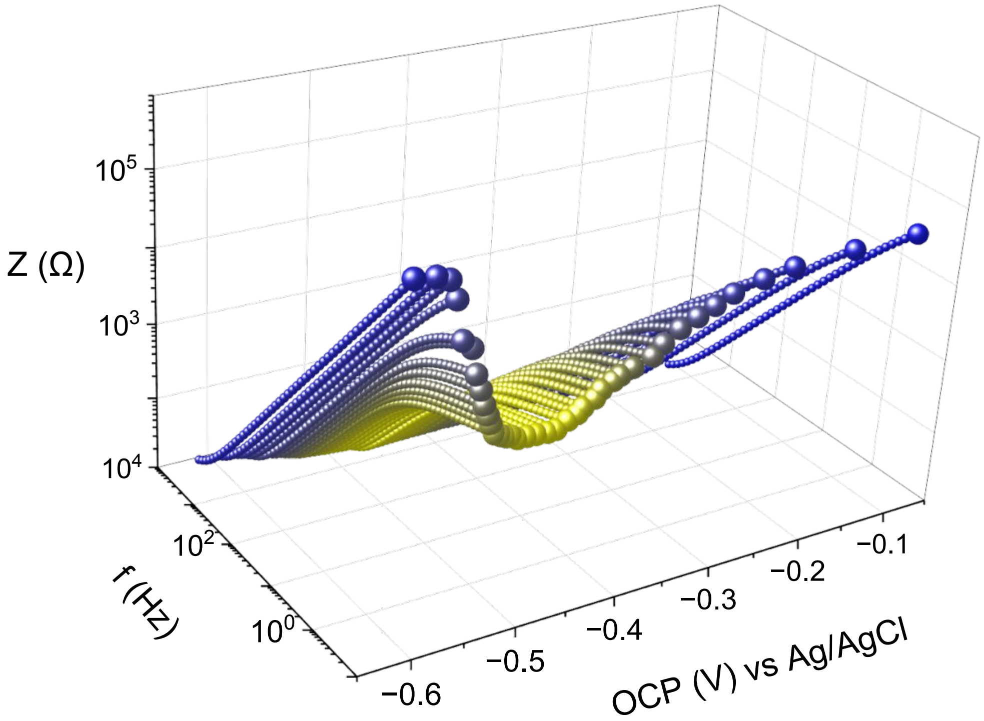

Figure S79. Bode plots of TAPT-NDI COF film at different OCP vs Ag/AgCl induced by 365 nm UV illumination at various time intervals. The measurements were performed in oxygen-free 0.1 M CsCl aqueous electrolyte in the presence of 10 mM sacrificial electron donor 4-MBA. The impedance data point for each measurement is magnified at the frequency of 0.1 Hz for better visualization of different redox states.

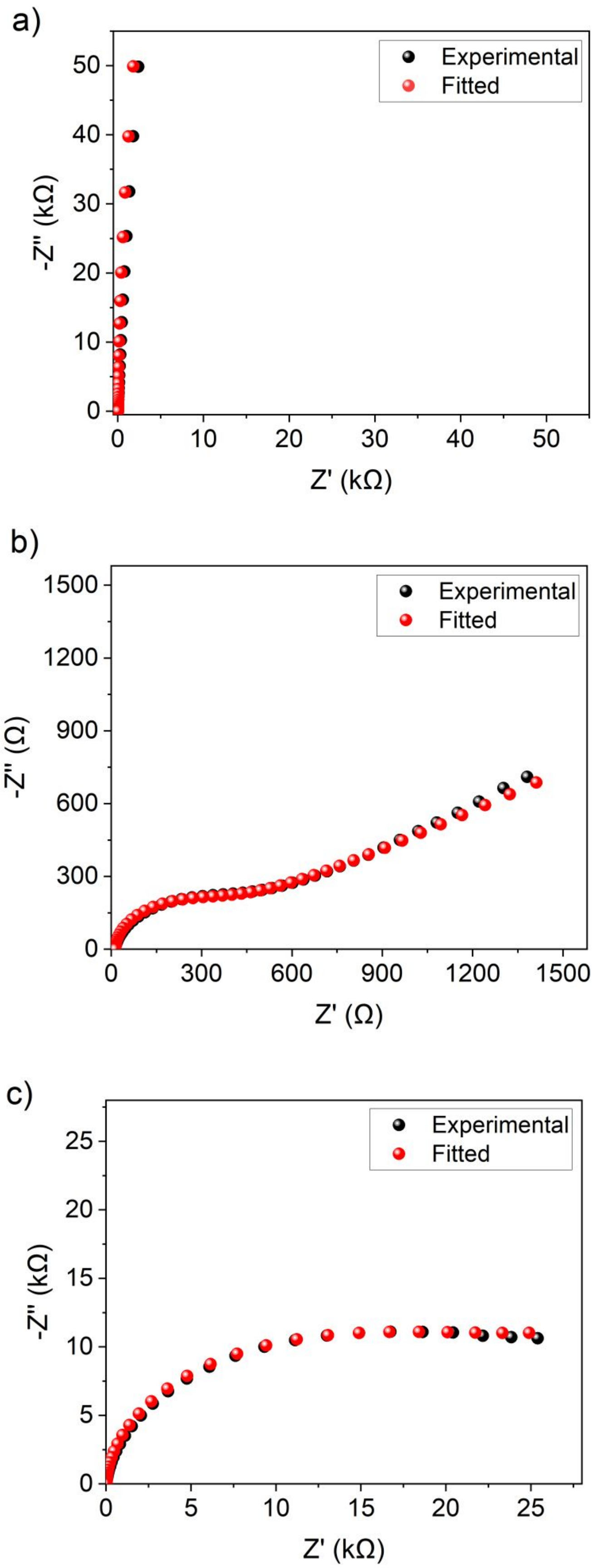

Figure S80. Representative Nyquist plots of experimental and fitted (circuit in Figure S65b) electrochemical impedance data for (a) 0.0-(TAPT-NDI), (b) 0.5-(TAPT-NDI), and (c) 1.0-(TAPT-NDI) films measured in 0.1 M KCl water electrolyte in the presence of 10 mM 4-MBA under UV irradiation.

Table S18. Fitting results of the EIS measurement for Figure S80a 0.0-(TAPT-NDI)

Parameters	Value	Fit Error	Fit Error (%)
R_S (Ω)	15.99	1.2	7.2
R_{ct} (Ω)	1.89E+7	7.4E+4	0.39
CPE Q 1	3.14E-06	1.25E-10	0.004
CPE Alpha 1	0.983	4.26E-05	0.043

Table S19. Fitting results of the EIS measurement for Figure S80b 0.5-(TAPT-NDI)

Parameters	Value	Fit Error	Fit Error (%)
R_S (Ω)	13.06	0.37	2.9
R_{ct} (Ω)	157.41	5.501	3.5
CPE Q 1	0.00076	3.025E-06	0.4
CPE Alpha 1	0.327	0.002	0.74
C_{ct} (F)	8.378E-06	8.78E-08	1

Table S20. Fitting results of the EIS measurement for Figure S80c 1.0-(TAPT-NDI)

Parameters	Value	Fit Error	Fit Error (%)
R_S (Ω)	16.76	1.049	18
R_{ct} (Ω)	6.507E+03	349.64	5.37
CPE Q 1	3.9061E-05	3.63E-07	0.24
CPE Alpha 1	0.206	0.0047	0.84
C_{ct} (F)	6.953E-06	9.53E-08	0.63

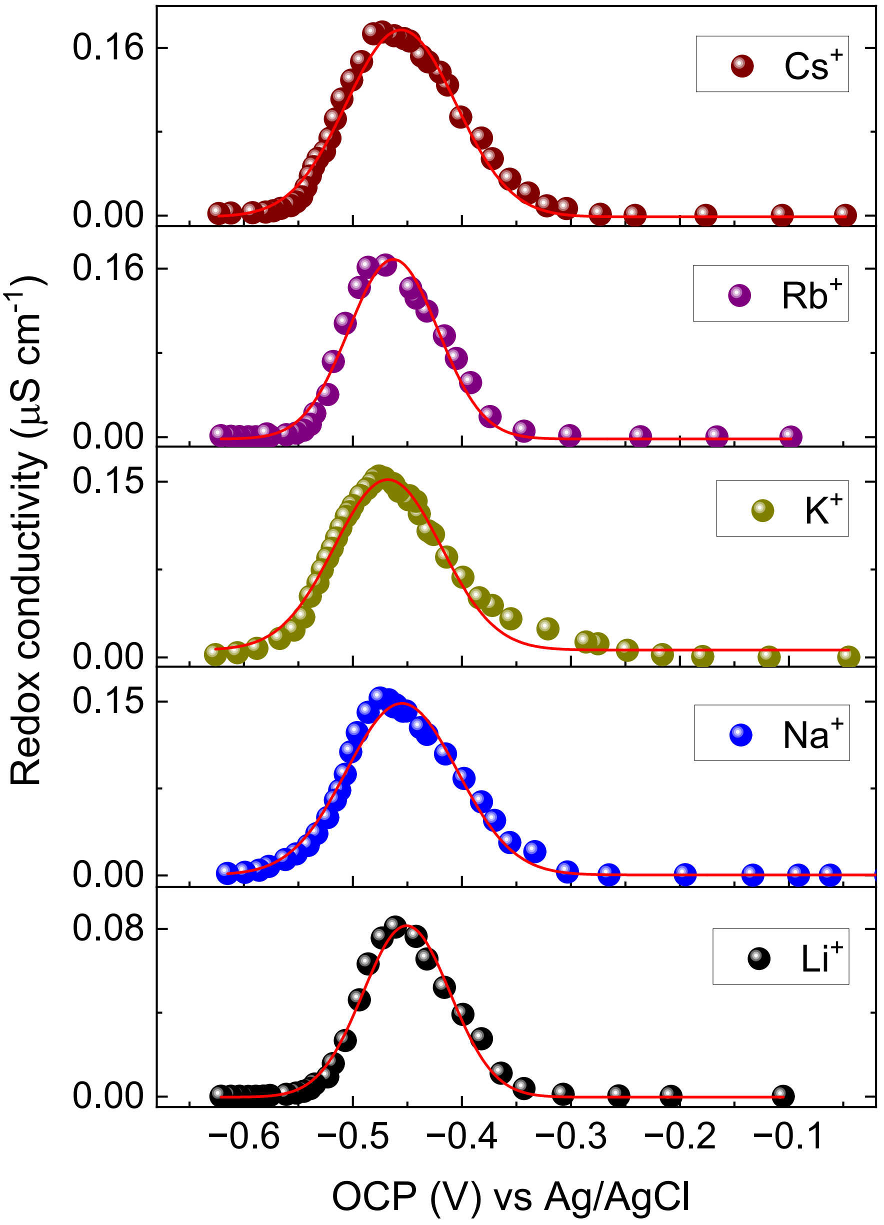

Figure S81. Evolution of the steady-state thin-film conductivity in aqueous solvent as a function of photoinduced open circuit potential in different supporting electrolytes. Steady-state photocharging experiments were performed in O_2 free 10 mM 4-MBA (sacrificial electron donor) aqueous solution with the supporting electrolytes.
